# Comparative efficacy of advanced treatments in biologic-naïve or biologic-experienced patients with ulcerative colitis: a systematic review and network meta-analysis

**DOI:** 10.1007/s11096-022-01509-1

**Published:** 2022-12-09

**Authors:** Xiaoyan Lu, James Jarrett, Susannah Sadler, Min Tan, James Dennis, Vipul Jairath

**Affiliations:** 1 Value and Market Access, Galapagos NV, Romainville, France; 2 HEOR Global Value and Access, Gilead Sciences Inc, Foster City, CA USA; 3 Health Economics and Outcomes Research Ltd, Cardiff, UK; 4 Value Communication, Health Economics and Outcomes Research Ltd, Cardiff, UK; 5 Department of Medicine, Division of Gastroenterology, Western University, London, ON Canada; 6 Department of Epidemiology and Biostatistics, Western University, London, ON Canada

**Keywords:** Comparative efficacy, Inflammatory bowel disease, Meta-analysis, Ulcerative colitis

## Abstract

**Background:**

Only one head-to-head comparison of advanced treatments in moderately to severely active ulcerative colitis (UC) has been published; therefore, there remains a need for further comparisons.

**Aim:**

The relative treatment effects of filgotinib and adalimumab, golimumab, infliximab, tofacitinib, ustekinumab and vedolizumab were estimated using a network meta-analysis (NMA).

**Method:**

Systematically identified studies (MEDLINE, Embase and Cochrane Library; searched: inception–May 2019, updated November 2020) investigating treatments for moderately to severely active UC were re-evaluated for inclusion in a Bayesian NMA (fixed-effects model). Relative treatment effects were estimated using different permutations of patient population (biologic-naïve or biologic-experienced), treatment phase (induction or maintenance) and outcomes (MCS response/remission or endoscopic mucosal healing).

**Results:**

Seventeen trials (13 induction; 9 maintenance) were included in the NMA; 8 treatment networks were constructed. Most targeted therapies were superior to placebo in terms of MCS response/remission and endoscopic mucosal healing; filgotinib 200 mg was similar to most other treatments. Infliximab 5 mg/kg was superior to filgotinib 200 mg (biologic-naïve; induction) for MCS response/remission (mean relative effect, 0.34 [95% credible interval: 0.05, 0.62]). Filgotinib 200 mg was superior to adalimumab 160/80/40 mg for MCS response/remission (biologic-experienced; induction; – 0.75 [– 1.16, – 0.35]), and endoscopic mucosal healing (biologic-naïve; maintenance; – 0.90 [– 1.89, – 0.01]); and to golimumab 50 mg every 4 weeks (biologic-naïve; maintenance; – 0.46 [– 0.94, 0]) for MCS response/remission.

**Conclusion:**

The current treatment landscape benefits patients with moderately to severely active UC, improving key outcomes; filgotinib 200 mg was similar to current standard of care in most outcomes.

**Supplementary Information:**

The online version contains supplementary material available at 10.1007/s11096-022-01509-1.

## Impact Statements

- Only one head-to-head comparison of advanced treatments for ulcerative colitis (UC) has been conducted. As the number of advanced therapies for UC increases, existing NMAs require continual updates to inform treatment decisions.
- This analysis confirms that the current treatment landscape benefits patients with moderately to severely active UC, and suggests that filgotinib 200 mg was similar to standard-of-care induction and maintenance treatment in terms of Mayo Clinic score response/remission and endoscopic mucosal healing, irrespective of previous biologic use.
- There is an unmet need for advanced treatments for UC with durable effectiveness. These analyses provide new evidence for a sub-class of a Janus kinase (JAK) inhibitors that preferentially inhibit JAK1 (i.e. filgotinib), which may help to inform clinical decision-making and treatment guidelines.

## Introduction

Ulcerative colitis (UC) is a chronic relapsing and remitting immune-mediated inflammatory bowel disease, characterised by diffuse inflammation of the colon and rectum [1]. Typical symptoms include rectal urgency, incontinence, fatigue, increased frequency of bowel movements, mucus discharge and abdominal discomfort [1]. Individuals with UC have impaired health-related quality of life [2], and the disease imposes a substantial direct and indirect economic burden [3, 4].

Conventional therapies for UC include corticosteroids and 5-aminosalicylates [5, 6]. For patients with inadequate response to conventional therapies, there are 7 advanced (biologic/targeted) therapies approved by the European Medicines Agency (EMA) for moderately to severely active UC: 5 biologics (the tumour necrosis factor antagonists infliximab [7], adalimumab [8] and golimumab [9]; the anti-integrin antibody vedolizumab [10]; and the interleukin (IL)-12/IL-23 antagonist ustekinumab [11]), the non-selective small molecule Janus kinase (JAK) inhibitor tofacitinib, and the once-daily, oral JAK1 preferential inhibitor filgotinib [12].

Only one head-to-head comparison of advanced treatments in UC has been conducted [13]; hence, several network meta-analyses (NMAs) have assessed indirect comparative efficacy across trials with different methods, populations and placebo responses [14–21]. NMAs can be used to estimate the relative effects of three or more interventions thus yielding more precise estimates than a single direct or indirect estimate, as well as allowing for ranking and hierarchy of the interventions compared [22]. Therefore, NMAs can be a useful tool to help inform treatment decisions as the number of advanced therapies for UC increases.

### Aim

In the absence of head-to-head trials, we estimated the relative treatment effects of filgotinib and key comparators (adalimumab, golimumab, infliximab, tofacitinib, ustekinumab and vedolizumab) in moderately to severely active UC using an NMA of data from randomised controlled trials (RCTs) of induction and maintenance phase treatments in patients who had not previously received biologics (biologic-naïve) and those who had (biologic-experienced).

## Method

### Protocol

This systematic review and NMA was performed in line with Preferred Reporting Items for Systematic Reviews and Meta-Analyses (PRISMA) and National Institute for Health and Care Excellence (NICE) guidelines [23, 24]. This review was not prospectively registered; protocol is available in Supplementary Material 1.

### Systematic literature review

#### Systematic and supplementary searches

Electronic databases (MEDLINE, Embase and the Cochrane Library) were interrogated from database inception to 8 May 2019, and subsequently updated to 2 November 2020. Searches (Supplementary Material 2) were designed to identify evidence (RCTs, open-label extensions and post hoc trial analyses) on advanced treatments with EMA approval in adult patients with moderately to severely active UC who cannot tolerate conventional therapy, or who have had an inadequate response or are refractory to conventional (biologic-naïve) or biologic (biologic-experienced) therapy. An additional search identified relevant evidence for ustekinumab, which received EMA approval after development of the initial search strategy (Supplementary Material 2). Additional supplementary searches (congresses [2016–2020], health technology and regulatory databases and reference lists) are outlined in Supplementary Material 2.

#### Study selection and data collection

Titles and abstracts were assessed for inclusion by two reviewers independently, against predefined eligibility criteria (Supplementary Material 2), and confirmed by two independent reviewers through evaluation of the full-text articles. Discrepancies were resolved by consensus or a third reviewer [24]. Study and patient characteristics, and efficacy and safety outcomes were extracted by a single reviewer into pre-specified data extraction tables; all records were checked against the source by a second reviewer. Studies included in the NMA were appraised for potential bias using Cochrane Collaboration’s tool [25].

### Network meta-analysis

#### Study and data selection

Trials were re-evaluated for inclusion in the NMA, and were restricted to phase 2/3 or phase 3 RCTs that used licensed doses of treatments with EMA approval for UC (November 2020) that reported clinical response, remission or mucosal healing during induction (6–16 weeks) or maintenance (48–56 weeks). Data on filgotinib were obtained from the study sponsor (Galapagos) [26]; two doses of filgotinib (200 mg and 100 mg) were considered in line with the registrational trial; however, only the results for filgotinib 200 mg, the licensed dose, are discussed further.

#### Outcomes

MCS is frequently used to classify UC and has previously been as the primary measure of efficacy to inform economic analysis in HTA submissions, supported by endoscopic mucosal healing [27–29]. Therefore, efficacy outcomes of interest were clinical response (MCS ≤ 2 points with no individual subscore of > 1), clinical remission (≥ 30% and ≥ 3-point decrease from baseline MCS and rectal bleeding subscore of 0–1, or ≥ 1-point decrease in baseline rectal bleeding subscore), and endoscopic mucosal healing (MCS endoscopic subscore of 0–1) during the induction or maintenance phases, as defined in the studies. The proportion of patients meeting each component of the efficacy outcome was extracted.

#### Network analyses and geometries

Networks were constructed based on patient population (biologic-naïve or biologic-experienced), treatment phase (induction or maintenance) and outcome (MCS response/remission or endoscopic mucosal healing).

#### Methods of analysis

A Bayesian NMA using a generalised linear model framework was adopted in line with NICE technical guidance. Analyses were conducted using both fixed and random effects, and the absolute model fit considered through examination of the total residual deviance; the fixed-effects model was preferred as the base case owing to the number of trials [23].

The primary endpoints of response and remission are binary variables; however, both are categorised based on MCS, a continuous score meaning response and remission can be analysed separately or together in a single model. In line with a recent NICE HTA submission, we used a single model using a multinomial likelihood with a probit link allowing for correlation between response and remission outcomes [29]. Endoscopic mucosal healing was analysed using a binomial likelihood with a logit link. Markov Chain Monte Carlo (MCMC) simulation was used to estimate posterior densities for treatment effects for all comparisons in the networks. Each analysis consisted of three MCMC chains with different starting values; inferences were derived from posterior distributions of the safety between treatments for outcomes of interest. The number of iterations for burn-in was 25 000 unless additional iterations were required to ensure convergence. Analyses were performed using R version 4.0.2 and WinBUGS version 1.4.3 with established NICE WinBUGS codes for NMA models.

#### Summary measures

Statistics for the posterior distribution of relative effects on the probit or logit scale and modelled probabilities of the outcomes were reported with 95% credible intervals (CrI), and treatments were ranked based on the predicted probability of the outcome.

#### Bias and heterogeneity assessments

Studies were assessed for heterogeneity in key effect modifiers (age, sex, weight, duration of disease, prior treatment, baseline Inflammatory Bowel Disease Questionnaire [IBDQ] score, disease severity, baseline MCS score and baseline concomitant treatment). Key areas of heterogeneity included: trial design (treat-through vs re-randomisation); definition of biologic failure; between-trial differences in treatment period; differential definitions of response or remission; and variation in placebo response in re-randomisation trials. To address these identified areas of heterogeneity, efficacy endpoints were evaluated separately in the induction and maintenance phases and treat-through trials were re-weighted so that maintenance phase response and remission outcomes were restricted to induction phase responders. A summary of potential bias including direction, magnitude and approach and details of the re-weighting calculations are outlined in Supplementary Material 2.

#### Sensitivity analyses

Sensitivity analyses were performed to test the impact of uncertainty on MCS response/remission; these included analyses of the following: definition of biologic failure (induction phase); differences in study design (treat-through vs re-randomised; maintenance phase); reporting differences (maintenance phase); and re-weighting methodology (maintenance phase).

## Results

### Identified evidence

**Fig. 1:**
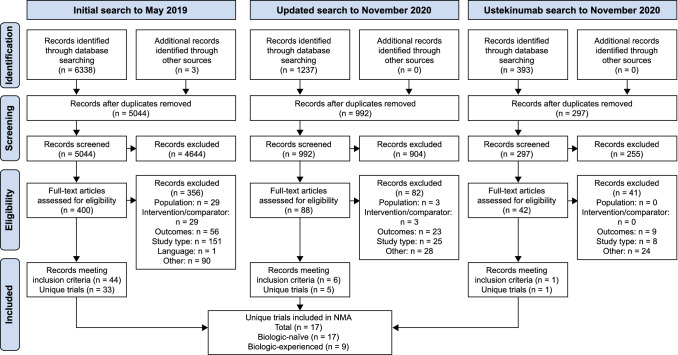
PRISMA diagram describing the process of identifying eligible trials for inclusion in the NMA. NMA, network meta-analysis; PRISMA, Preferred Reporting Items for Systematic Reviews and Meta-Analyses

Seventeen systematically identified trials were included in the NMA: ACT1 [30], ACT2 [30], GEMINI 1 [31], Jiang 2015 [32], Kobayashi 2016 [33], NCT01551290 [34], OCTANE SUSTAIN [35], OCTAVE 1 [36], OCTAVE 2 [36], PURSUIT-SC Induction [37], PURSUIT-SC Maintenance [37], SELECTION [26], ULTRA 1 [38], ULTRA 2 [39, 40], UNIFI [41], VARSITY [13] and VISIBLE 1 [42]. Thirteen trials reported outcomes in the induction phase [13, 26, 30–34, 36–41] (all included patients who were biologic-naïve and 7 included biologic-experienced patients [13, 26, 31, 36, 39–41]), and 9 [13, 26, 35, 37, 39–42] reported outcomes in the maintenance phase (all included patients who were biologic-naïve and 7 [13, 26, 31, 35, 39–42] included biologic-experienced patients). A PRISMA diagram is shown in Fig. 1, and trials that were identified by the systematic review but were not eligible for inclusion in the NMA are included in the Supplementary Material 2 alongside study characteristics and efficacy outcomes for included studies. The Cochrane assessment of within-trial bias revealed 12 studies with a low risk of bias, 5 studies with some risk and zero trials with a high risk (Supplementary Material 2).

### Patient characteristics

Baseline characteristics were similar across trials in terms of age, sex and weight. Owing to between-trial heterogeneity, separate analyses were conducted for biologic-naïve and biologic-experienced patients. IBDQ score and disease severity were poorly reported (no studies and two studies, respectively); therefore, the effect of these modifiers is unclear. The re-weighted maintenance phase MCS response/remission outcomes of treat-through trials (ULTRA 2 and VARSITY) are given in the Supplementary Material 2.

### Induction phase analyses

League tables of all NMA results by treatment phase, endpoint and population are included in Supplementary Material 2.

#### MCS response/remission – biologic-naïve

**Fig. 2:**
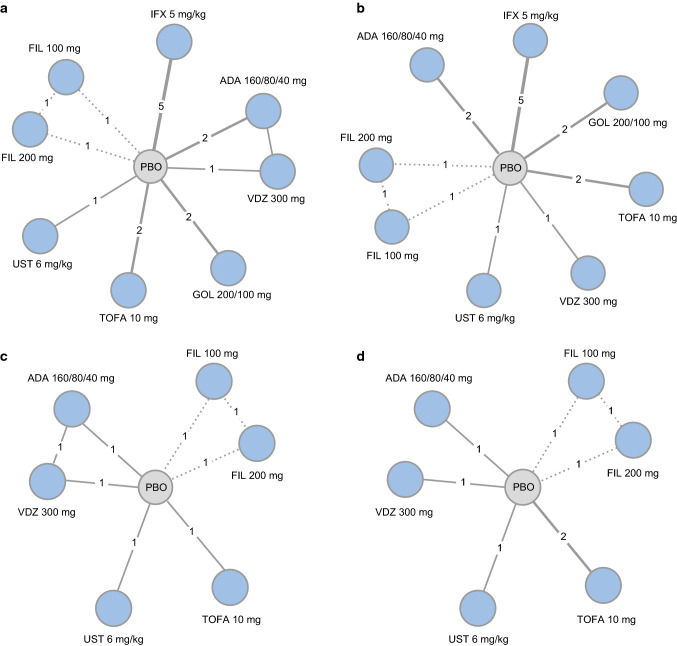
Network geometries for biologic-naïve (MCS response/remission [**a**] and endoscopic mucosal healing [**b**]) and biologic-experienced (MCS response/remission [**c**] and endoscopic mucosal healing [**d**]) patients in the induction phase. ADA, adalimumab; BID, twice daily; FIL, filgotinib; GOL, golimumab; IFX, infliximab; IL, interleukin; JAK, Janus kinase; MCS, Mayo Clinic Score; PBO, placebo; QD, once daily; QXW, every X weeks; SC, subcutaneous; TNF, tumour necrosis factor; TOFA, tofacitinib; UST, ustekinumab; VDZ, vedolizumab

**Fig. 3:**
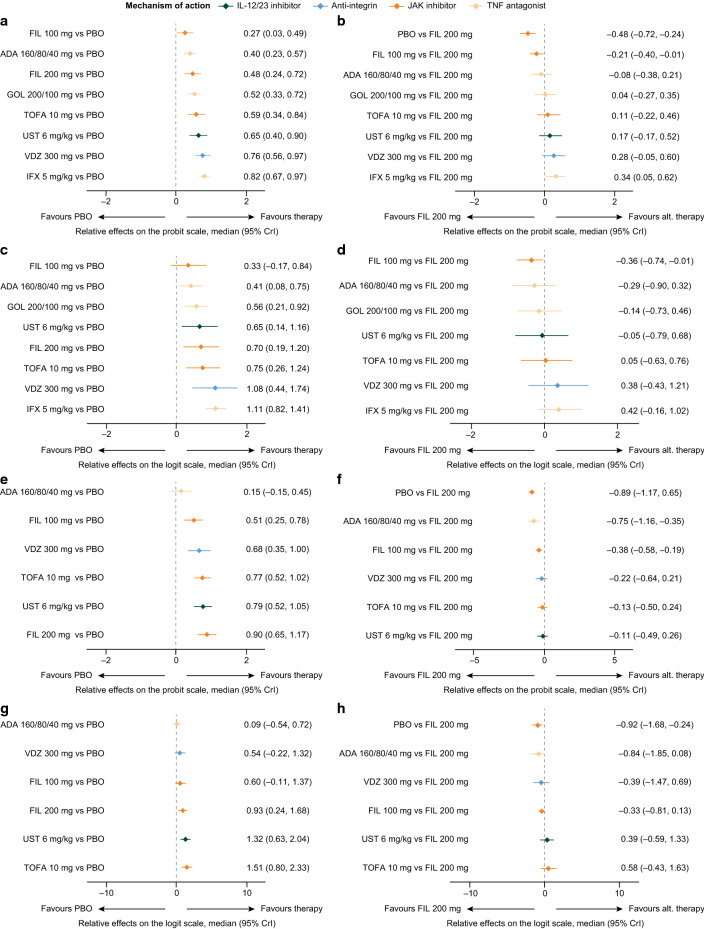
Relative effects of induction phase treatments. MCS response/remission (versus placebo [**a**] and versus filgotinib 200 mg [**b**]) and endoscopic mucosal healing (versus placebo [**c**] and versus filgotinib 200 mg [**d**]) in biologic-naïve patients and MCS response/remission (versus placebo [**e**] and versus filgotinib 200 mg [**f**]) and endoscopic mucosal healing (versus placebo [**g**] and versus filgotinib 200 mg [**h**]) in biologic-experienced patients. ADA, adalimumab; alt., alternative; BID, twice daily; CrI, credible interval; FIL, filgotinib; GOL, golimumab; IFX, infliximab; IL, interleukin; JAK, Janus kinase; MCS, Mayo Clinic score; PBO, placebo; QD, once daily; QXW, every X weeks; SC, subcutaneous; TNF, tumour necrosis factor; TOFA, tofacitinib; UST, ustekinumab; VDZ, vedolizumab

The analysis network for MCS response/remission (Fig. 2a) and endoscopic mucosal healing (Fig. 2b) in biologic-naïve patients comprised 9 treatment groups (adalimumab 160/80/40 mg, filgotinib 100 mg, filgotinib 200 mg, golimumab 200/100 mg, infliximab 5 mg/kg, placebo, tofacitinib 10 mg, ustekinumab 6 mg/kg and vedolizumab 300 mg) across 13 studies [26, 30–34, 36–41]. All interventions were statistically superior to placebo (Fig. 3a). The effects on MCS response/remission were similar between filgotinib 200 mg and other interventions with the exception of infliximab 5 mg/kg, which demonstrated statistical superiority over filgotinib 200 mg (mean relative effect, filgotinib 200 mg vs infliximab 5 mg/kg [95% CrI], 0.34 [0.05, 0.62]; Fig. 3b).

#### Endoscopic mucosal healing: biologic-naïve

All interventions were statistically superior to placebo with regard to endoscopic mucosal healing (Fig. 3c). Treatment effects were similar between filgotinib 200 mg and all comparators (Fig. 3d).

#### MCS response/remission: biologic-experienced

The analysis network for MCS response/remission in biologic-experienced patients comprised seven treatment groups (adalimumab 160/80/40 mg, filgotinib 100 mg, filgotinib 200 mg, placebo, tofacitinib 10 mg, ustekinumab 6 mg/kg and vedolizumab 300 mg) across seven studies (Fig. 2c) [13, 26, 31, 36, 39–41]. All interventions were statistically superior to placebo, with the exception of adalimumab 160/80/40 mg (Fig. 3e). Treatment effects were similar between filgotinib 200 mg and all other comparators except for adalimumab 160/80/40 mg, over which filgotinib 200 mg was statistically superior (mean relative effect filgotinib 200 mg vs adalimumab 160/80/40 mg [95% CrI], – 0.75 [– 1.16, – 0.35]; Fig. 3f).

#### Endoscopic mucosal healing: biologic-experienced

In the biologic-experienced population, the analysis network for endoscopic mucosal healing comprised seven treatment groups (adalimumab 160/80/40 mg, filgotinib 100 mg, filgotinib 200 mg, placebo, tofacitinib 10 mg, ustekinumab 6 mg/kg and vedolizumab 300 mg) across 6 studies (Fig. 2d) [26, 31, 36, 39–41]. Adalimumab 160/80/40 mg and vedolizumab 300 mg were similar to placebo, and filgotinib 200 mg, tofacitinib 10 mg and ustekinumab 6 mg/kg were statistically superior to placebo (Fig. 3g). Treatment effects were similar between filgotinib 200 mg and all other interventions (Fig. 3h).

### Maintenance phase analyses

League tables of all NMA results by treatment phase, endpoint and population are included in Supplementary Material 2.

#### MCS response/remission: biologic-naïve

**Fig. 4:**
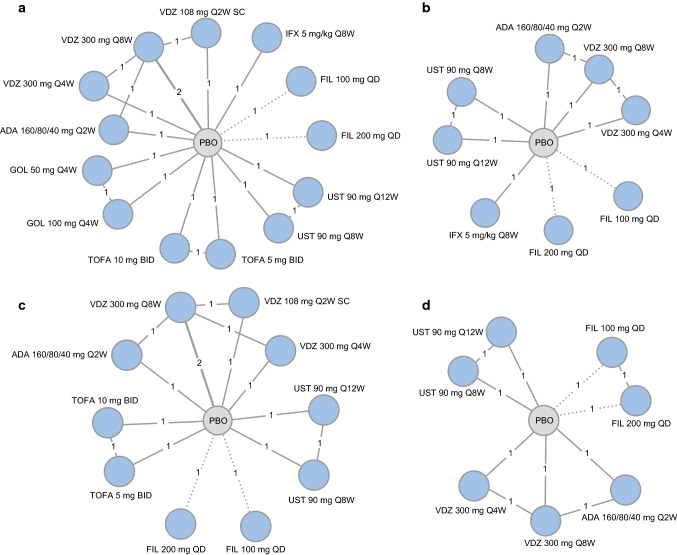
Network geometries for biologic-naïve (MCS response/remission [**a**] and endoscopic mucosal healing [**b**]) and biologic-experienced (MCS response/remission [**c**] and endoscopic mucosal healing [**d**]) patients in the maintenance phase. ADA, adalimumab; BID, twice daily; FIL, filgotinib; GOL, golimumab; IFX, infliximab; IL, interleukin; JAK, Janus kinase; MCS, Mayo Clinic Score; PBO, placebo; QD, once daily; QXW, every X weeks; SC, subcutaneous; TNF, tumour necrosis factor; TOFA, tofacitinib; UST, ustekinumab; VDZ, vedolizumab

**Fig. 5:**
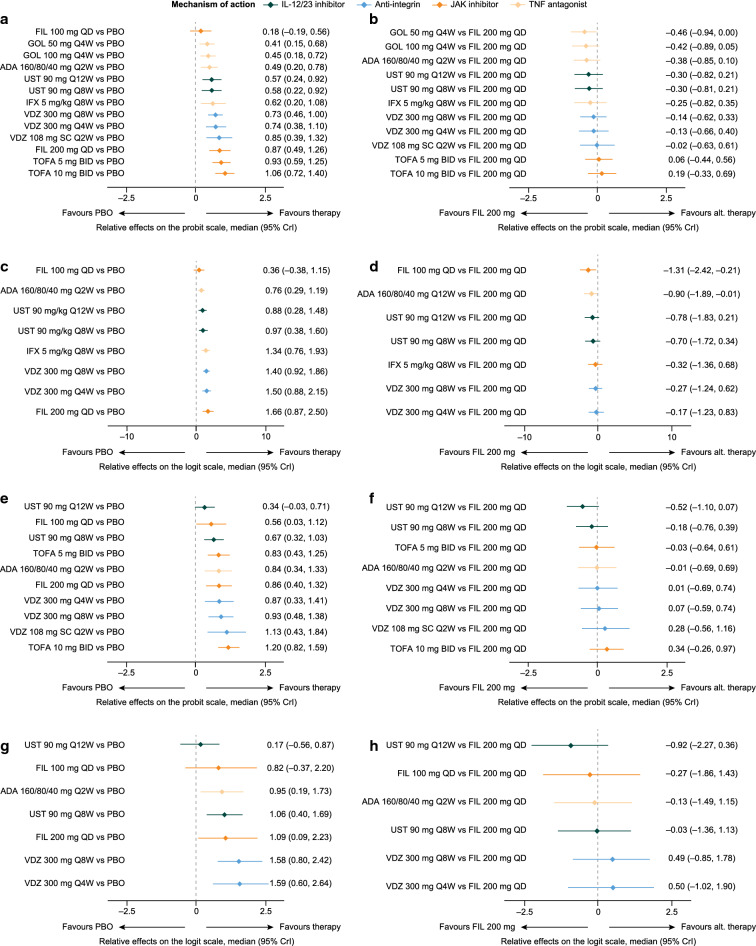
Relative effects of maintenance phase treatments. MCS response/remission (versus placebo [**a**] and versus filgotinib 200 mg [**b**]) and endoscopic mucosal healing (versus placebo [**c**] and versus filgotinib 200 mg [**d**]) in biologic-naïve patients and MCS response/remission (versus placebo [**e**] and versus filgotinib 200 mg [**f**]) and endoscopic mucosal healing (versus placebo [**g**] and versus filgotinib 200 mg [**h**]) in biologic-experienced patients. ADA, adalimumab; alt., alternative; BID, twice daily; CrI, credible interval; FIL, filgotinib; GOL, golimumab; IFX, infliximab; IL, interleukin; JAK, Janus kinase; MCS, Mayo Clinic score; PBO, placebo; QD, once daily; QXW, every X weeks; SC, subcutaneous; TNF, tumour necrosis factor; TOFA, tofacitinib; UST, ustekinumab; VDZ, vedolizumab

The analysis network for MCS response/remission in biologic-naïve patients comprised 14 treatment groups (adalimumab 160/80/40 mg, filgotinib 100 mg, filgotinib 200 mg, golimumab 50 mg, golimumab 100 mg, infliximab 5 mg/kg, placebo, tofacitinib 5 mg, tofacitinib 10 mg, ustekinumab 90 mg every 8 weeks [Q8W], ustekinumab 90 mg every 12 weeks [Q12W], vedolizumab 108 mg subcutaneous [SC], vedolizumab 300 mg every 4 weeks [Q4W] and vedolizumab 300 mg Q8W) across 9 studies (Fig. 4a) [13, 26, 30, 31, 35, 37, 39–42]. All interventions were statistically superior to placebo (Fig. 5a). Treatment effects were similar between filgotinib 200 mg and all comparators except for golimumab 50 mg Q4W, to which filgotinib 200 mg was statistically superior (mean relative effect filgotinib 200 mg golimumab 50 mg [95% CrI], – 0.46 [– 0.94, 0.00]; Fig. 5b).

#### Endoscopic mucosal healing: biologic-naïve

In the biologic-naïve population, the analysis network for endoscopic mucosal healing comprised 9 treatment groups (adalimumab 160/80/40 mg, filgotinib 100 mg, filgotinib 200 mg, infliximab 5 mg/kg, placebo, ustekinumab 90 mg Q8W, ustekinumab 90 mg Q12W, vedolizumab 300 mg Q4W and vedolizumab 300 mg Q8W) across 6 studies (Fig. 4b) [13, 26, 30, 31, 39–41]. All interventions were statistically superior to placebo (Fig. 5c). Treatment effects were similar between filgotinib 200 mg and all other interventions except for adalimumab 160/80/40 mg, over which filgotinib 200 mg was statistically superior (mean relative effect vs adalimumab 160/80/40 mg vs filgotinib 200 mg [95% CrI], – 0.90 [– 1.89, – 0.01]; Fig. 5d).

#### MCS response/remission: biologic-experienced

The analysis network for MCS response/remission in biologic-experienced patients comprised 11 treatment groups (adalimumab 160/80/40 mg, filgotinib 100 mg, filgotinib 200 mg, placebo, tofacitinib 5 mg, tofacitinib 10 mg, ustekinumab 90 mg Q8W, ustekinumab 90 mg Q12W, vedolizumab 108 mg SC, vedolizumab 300 mg Q4W and vedolizumab 300 mg Q8W) across 7 studies (Fig. 4c) [13, 26, 31, 35, 39–42]. All interventions were statistically superior to placebo, except for ustekinumab 90 mg Q12W (Fig. 5e). Treatment effects were similar between filgotinib 200 mg and all interventions (Fig. 5f).

#### Endoscopic mucosal healing: biologic-experienced

In the biologic-experienced population, the analysis network for endoscopic mucosal healing comprised 8 treatment groups (adalimumab 160/80/40 mg, filgotinib 100 mg, filgotinib 200 mg, placebo, ustekinumab 90 mg Q8W, ustekinumab 90 mg Q12W, vedolizumab 300 mg Q4W and vedolizumab 300 mg Q8W) across five studies (Fig. 4d) [13, 26, 31, 39–41].

All comparators were statistically superior to placebo with the exception of ustekinumab 90 mg Q12W (Fig. 5a). Treatment effects were similar between filgotinib 200 mg and all other interventions (Fig. 5b).

### Sensitivity analyses

Variations in key areas of uncertainty (definition of biologic failure, differences between studies and re-weighting methodology) had limited impact on the relative probability of patients achieving MCS response/remission in the induction and maintenance phases in both the biologic-naïve and biologic-experienced populations (Supplementary Material 2).

## Discussion

Only one head-to-head comparison of advanced treatments in moderately to severely active UC has been published; hence, there remains a need for methods that provide indirect comparison, such as NMA. We present the outcomes of, to our knowledge, the first NMA of approved therapies stratified by treatment phase (induction and maintenance) and previous biologic treatment that also included filgotinib.

This analysis demonstrated that most targeted therapies were superior to placebo and that filgotinib 200 mg was equally effective as other advanced therapies (adalimumab, golimumab, tofacitinib, ustekinumab and vedolizumab) in terms of MCS response/remission and endoscopic mucosal healing was broadly similar to other interventions including adalimumab, golimumab, tofacitinib, ustekinumab and vedolizumab, irrespective of treatment setting and biologic status. Statistical superiority for MCS response/remission in the induction phase was estimated for infliximab 5 mg/kg over filgotinib 200 mg (biologic-naïve), and filgotinib 200 mg over adalimumab 160/80/40 mg (biologic-experienced). In the maintenance phase, filgotinib 200 mg was statistically significant superior to golimumab 50 mg Q4W and adalimumab 160/80/40 mg (both biologic-naïve) in MCS response/remission and endoscopic mucosal healing, respectively.

The differences estimated between filgotinib 200 mg and infliximab, adalimumab and golimumab, may, in part, reflect changes in the disease landscape between the development of these drugs. Infliximab represents the first targeted EMA-approved treatment for use in UC (2006) [43] based on the treat-through ACT1 and ACT 2 trials [30]. Although the sensitivity analyses suggest that the inclusion of treat-through trials has minimal impact on MCS response/remission outcomes, differences arising from local versus central assessment of endoscopies could not be estimated. Local assessment carries a greater probability of bias than central assessment; therefore, the exclusive use of local assessment in trials of infliximab [30] and golimumab [37] (the only trials included in this analysis to do so), may have introduced uncertainty surrounding outcomes. Furthermore, patients in the earlier ACT1 and ACT2 trials had not previously received biologics [30], and therefore may represent a population less refractory to treatment than in later trials.

### Strength and limitations

NMAs aid clinicians and patients who are faced with more complex decision-making processes as the number of available treatments increases, and are particularly valuable given that head-to-head trials are likely to continue to be a rarity. This analysis employed well-established methods in line with NICE technical guidance. The networks constructed were generally large (up to 14 interventions from 9 trials), allowing comparison of filgotinib with treatments licensed for use in UC at the time of analysis. Furthermore, the use of a single model approach to estimate response and remission, which was supported by the Evidence Review Group as part of a NICE appraisal [29], takes into account correlation between response and remission preventing the occurrence of incompatible results (e.g. proportion of remitters > proportion of responders).

As with all estimates of treatment efficacy, there are inherent limitations. While areas of between-trial heterogeneity were identified and addressed, not all heterogeneity could be controlled for or tested owing to the size of the networks (generally one comparison per drug). The inclusion of a mixture of treat-through and re-randomisation trials was addressed through recalculation of the maintenance phase outcomes to reflect the treat-through design but ensure randomisation is maintained. Exclusion of treat-through trials (ACT1, ULTRA 2 and VARSITY) suggested that including these trials in the base case had minimal impact. However, because some re-randomisation trials had ‘true’ placebo–placebo arms (i.e. no carryover) whereas others had treatment–placebo arms with some potential carryover of effect, potential bias may have arisen, the extent of which cannot be estimated. Any impact may be greater for drugs with longer dosing intervals and half-lives.

Interestingly, any discrepancies in the definitions of response and remission between trials, as previously described in the literature [44], had a low impact on the effect on relative remission versus placebo, and there was sufficient similarity between the measures to allow comparison. However, the extent of bias introduced through differences in methodologies of central reading of endoscopies could not be estimated. As discussed previously, approximately half of the included trials used local reading of endoscopies, which is more likely to introduce bias than central reading, and thus raises the possibility of residual confounding factors.

The S1P receptor agonist ozanimod and the JAK inhibitors peficitinib and upadacitinib were not included in the analysis because, at the time of the analysis, they were not approved by the EMA for use in UC; however, future NMAs in this area may incorporate data from these compounds as they become available. Head-to-head clinical trial data are needed to provide the most robust evidence of comparative efficacy, but in the absence of these, further research could be performed using advanced methodology such as matching-adjusted indirect comparisons using individual patient data from trials.

## Conclusion

This NMA suggests that the current treatment landscape benefits patients with moderately to severely active UC, improving key outcomes such as MCS response/remission and endoscopic mucosal healing. Filgotinib, which was approved by the EMA for use in UC in January 2022, was generally associated with similar outcomes as current standard of care.

## Supplementary Information

Below is the link to the electronic supplementary material.

Supplementary file1 (DOCX 63 KB)

Supplementary file2 (DOCX 374 KB)
