# Correction to: Comparative efficacy of advanced treatments in biologic-naïve or biologic-experienced patients with ulcerative colitis: a systematic review and network meta-analysis

**DOI:** 10.1007/s11096-023-01544-6

**Published:** 2023-03-19

**Authors:** Xiaoyan Lu, James Jarrett, Susannah Sadler, Min Tan, James Dennis, Vipul Jairath

**Affiliations:** 1 Value and Market Access, Galapagos NV, Romainville, France; 2 HEOR Global Value and Access, Gilead Sciences Inc, Foster City, CA USA; 3 Health Economics and Outcomes Research Ltd, Cardiff, UK; 4 Value Communication, Health Economics and Outcomes Research Ltd, Cardiff, UK; 5 Department of Medicine, Division of Gastroenterology, Western University, London, ON Canada; 6 Department of Epidemiology and Biostatistics, Western University, London, ON Canada

**Correction to: International Journal of Clinical Pharmacy** 10.1007/s11096-022-01509-1

In the original publication of the article, the figures 3 and 5 are incorrectly swapped. 

The article Comparative efficacy of advanced treatments in biologic-naïve or biologic-experienced patients with ulcerative colitis: a systematic review and network meta-analysis, written by Xiaoyan Lu, James Jarrett, Susannah Sadler, Min Tan, James Dennis, Vipul Jairath, was originally published electronically on the publisher’s internet portal on 9th December 2022 without open access.

With the author(s)’ decision to opt for Open Choice the copyright of the article changed on 16th January 2023 to © The Authors 2022 and the article is forthwith distributed under a Creative Commons Attribution 4.0 International License, which permits use, sharing, adaptation, [50] distribution and reproduction in any medium or format, as long as you give appropriate credit to the original author(s) and the source, provide a link to the Creative Commons licence, and indicate if changes were made. The images or other third party material in this article are included in the article’s Creative Commons licence, unless indicated otherwise in a credit line to the material. If material is not included in the article’s Creative Commons licence and your intended use is not permitted by statutory regulation or exceeds the permitted use, you will need to obtain permission directly from the copyright holder. To view a copy of this licence, visit http://creativecommons.org/licenses/by/4.0.

The original article has been updated.

