# Supplementary material for: Comparative efficacy of advanced treatments in biologic-naïve or biologic-experienced patients with ulcerative colitis: a systematic review and network meta-analysis
Source: Int J Clin Pharm. 2022 Dec 9;45(2):330–41. doi: 10.1007/s11096-022-01509-1 (PMC10147762; doi:10.1007/s11096-022-01509-1)
Supplement: Supplementary file 1 — Supplementary file1 (DOCX 63 KB) [file 11096_2022_1509_MOESM1_ESM.docx]

**Comparative efficacy of advanced treatments in biologic-naïve or biologic-experienced patients with ulcerative colitis: a systematic review and network meta-analysis – Supplementary Material 1**

##

# Protocol – systematic literature review (original and update)

## Objective

The specific research question of this review is:

- What is the clinical efficacy and safety of interventions for the induction and maintenance treatment of moderately to severely active UC in adults when conventional therapy cannot be tolerated or the disease has responded inadequately or lost response to conventional (biologic-naïve) or biologic (biologic experienced/failed) treatment?

The objective of this specific protocol is:

- To conduct a systematic literature review update to identify recently published literature (between August 2019 and present) describing efficacy and safety of interventions for the induction and maintenance treatment of moderately to severely active UC.

## Methods

This protocol has been designed according to the Preferred Reporting Items for Systematic review and Meta-Analysis Protocols (PRISMA-P) checklist [[1](#_ENREF_1)].

#### Eligibility criteria

Eligibility criteria will be applied following the Population-Intervention-Comparators-Outcomes-Study (PICOS) framework, in line with PRISMA-P guidance. Table 1 shows PICOS eligibility criteria for the review.

The PICOS aligns with the PICOS in TA547 (Tofacitinib for moderate to severe UC) [[2](#_ENREF_2)]. TA342 (Vedolizumab for moderate to severe UC)[[3](#_ENREF_3)] and TA329, a multiple technology assessment infliximab, adalimumab and golimumab for UC in adults whose disease has responded inadequately, or are contraindicated to conventional therapy [[4](#_ENREF_4)].

In these HTAs, except for TA547, study design of included studies was limited to randomised controlled trial (RCT) evidence only. All three studies conducted a network meta-analysis (NMA) to derive comparative efficacy and therefore studies that did not have an intervention of interest in more than 1 arm and single arm studies were excluded.

It was assumed that the study populations would align to the study populations of the SELECTION1 phase 2b/3 clinical trial programmes, which investigated the clinical efficacy and safety of filgotinib induction and maintenance treatment of moderately to severely active ulcerative colitis (UC) in participants who are biologic-naive and biologic-experienced.

### Changes from the original SLR

- During the course of the original SLR in 2019, post-hoc analyses were excluded. It has since been agreed that these studies are to be included in the review in order to gather data for analysis on the biologic-naïve and biologic experienced/failed subgroups. Therefore, the studies that were excluded in the original SLR will be re-assessed using the PICOS criteria below and any post-hoc analyses meeting inclusion criteria will be included in this update.
- The original SLR did not include ustekinumab in the list of interventions, therefore additional searches are carried out specifically for ustekinumab, without date limits (see Table 7 to 10 in Appendix A).

Table 1. Eligibility criteria for studies describing the clinical efficacy and safety of interventions for the induction and maintenance treatment of moderately to severely active ulcerative colitis (UC)

|  | **Inclusion criteria** | **Exclusion criteria** |
| --- | --- | --- |
| **Population** | - Adult (≥18 years of age) patients with moderately to severely active ulcerative colitis in adults when conventional therapy cannot be tolerated or the disease has responded inadequately or lost response to conventional^§^ (biologic-naïve) or biologic (biologic experienced/failed) treatment*. | - Juvenile or paediatric ulcerative colitis - Presence of Crohn's disease, indeterminate colitis, ischemic colitis, fulminant colitis, ulcerative proctitis, or toxic mega-colon - Patients with mild UC; if the study population is mixed (i.e., mild to severe), exclude those studies in which data are not reported separately for moderate or severely active UC - Patients without UC |
| **Interventions** | - Biologic drugs, including: - Adalimumab (Humira, Trudexa, ABP 501, BI695501, CHS-1420, GP2017, M923, PF-06410293) - Apremilast (Otezla) - Golimumab (Simponi) - Infliximab (Remicade) - Ustekinumab (Stelara) - Vedolizumab (Entyvio) - Biosimilars, including: - Adalimumab biosimilars (Amjevita/ABP-501; Cyltezo/BI 695501; SB5) - Infliximab biosimilars (Remsima; Inflectra; Flixabi; Renflexis/SB2, CT-P13; PF-06438179; PF-06438179; ABP501) - Targeted synthetic drugs including: - Baricitinib (Oluminant) - Tofacitinib (Xeljanz) - Filgotinib (GLPG0634, GS-6034) - Peficitinib (ASP015K) - Upacitinib (ABT-494) - PF-06651600 - PF-06700841 - TD-1473 - Surgical procedures for managing moderate to severe ulcerative colitis | - Studies that do not have an intervention of interest in more than 1 arm - Non-pharmacological studies, e.g., exercise, Chinese medicine, etc. - Studies only comparing conventional therapies including aminosalicylates and corticosteroids |
| **Comparators** | - Any comparison between any of the listed interventions and each other or placebo | - Studies not reporting on at least one of the interventions of interest |
| **Outcomes** | †To be included in the review, a study must report at least 1 of the following outcomes of interest:   - †Efficacy measurements: - Mayo Clinic Score (MCS). - Partial Mayo Score - Ulcerative colitis symptom score. - Clinical response - Histologic remission - Clinical Remission - Corticosteroid-free remission - Endoscopic/Mucosal healing - Surgery - †Safety outcomes reported at study endpoint: - Overall rate of AEs - Overall rate of serious AEs - Discontinuations due to adverse events - Lack of efficacy - AEs - Individual AEs, such as the following: - Arthralgia - Infections, including herpes zoster - Nasopharyngitis - Intestinal perforation - Death - Initial or prolonged inpatient hospitalisation | - Outcomes of interest not reported |
| **Study design** | - Randomised, controlled, prospective clinical trials (above phase 1) - RCTs in which patients are re-randomised at the end of induction - RCTs in which patients are stay in their randomised groups at the end of induction (treat-through) - Long-term follow-up studies (e.g. open-label follow-up studies with continuation of treatments in their respective randomised group) - Post hoc analyses of patient sub-groups of interest (biologic-naïve/biologic experienced/biologic failed) | - Phase 1 studies - Non-randomised clinical trials - Single-arm studies - Long-term follow-up or extension studies of RCTs of over 1 year (post maintenance phase data) - Maintenance studies and step-down treatment studies - Preclinical studies - Prognostic studies - Retrospective observational studies - Prospective observational studies - Case report - Case series - Animal models |
| **Publication type** | - Peer-reviewed publications - Clinical trial records - Conference proceedings | - Commentaries and letters (publication type) - Pooled analyses - Non-systematic reviews - Systematic reviews (including meta-analyses)† - Consensus reports |
| **Language restrictions** | - English language only | - Studies published in languages other than English |
| **Date restrictions** | - SLR update: after 07.05.2019^¶^ | - SLR update: Studies published before 07.05.2019^¶^ |
| AE: adverse event; UC: ulcerative colitis; SLR: systematic literature review; VAS: visual analogue scale.  *Disease severity is defined according to the Truelove and Witts’ severity index in line with NICE clinical guidance [[5](#_ENREF_5)]. Moderate to severely active ulcerative colitis: total Mayo score of 6 to 12.  §Conventional therapy considered to include topical or oral aminosalicylate, corticosteroids, mercaptopurine, azathioprine or prednisolone  †Systematic reviews and meta-analyses will be used for identification of primary studies that may have been missed in the electronic searches  ^¶^ Cut-off date based on the date the searches for the original SLR were conducted; additional searches for ustekinumab do not have date restrictions. | | |

#### Information sources

Searches will be conducted to May 2019 (original SLR) and from May 2019 (SLR update), and will be conducted across the following electronic databases:

- PubMed (Medline and Medline In-Process)
- Embase
- The Cochrane Library
  - Cochrane Database of Systematic Reviews (CDSR)
  - Cochrane Central Register of Controlled Trials (CENTRAL)
  - Cochrane Clinical Answers (CCA)
- The University of York Centre for Reviews and Dissemination (CRD)
  - Health Technology Assessment database (HTA)
  - Abstracts of Reviews of Effects (DARE)
  - NHS Economic Evaluation database (NHS-EED)

In addition to the searching of databases, the following conference proceedings (2016 to present [original SLR] and from May 2019 to present [SLR update]) will be searched for any ongoing or completed clinical trials for filgotinib and its comparators that may not have been identified from the searches of relevant databases:

- American College of Gastroenterology (ACG)
- Crohn's and Colitis UK
- European Crohn’s and Colitis Organisation (ECCO)
- British Society of Gastroenterology
- International Society for Pharmacoeconomics and Outcomes Research (ISPOR)

To identify clinical trials of filgotinib and its comparators, the following websites will be searched:

- ClinicalTrials.gov:
  - <http://clinicaltrials.gov/>
- International Clinical Trials Registry Platform:
  - <http://www.who.int/ictrp/en/>
- European Union’s Clinical Trials Register:
  - <http://www.clinicaltrialsregister.eu/>
- Klinische Prüfungen PharmNet.Bund:
  - <http://www.pharmnet-bund.de/dynamic/de/klinische-pruefungen/>

Reference lists of any relevant studies, recent systematic reviews, and meta-analyses will be searched for further studies of interest. In addition, reference lists of relevant articles identified from the following sources will be searched:

- Scottish Medicine Consortium advice
- NICE’s multiple technology appraisal and single technology appraisal documents
- United States Food and Drug Administration register
- European public assessment reports for human medicines, published by the European Medicines Agency

Supplemental to the formal SLR, additional manufacturer data on file available for filgotinib will be used.

#### Search strategy

Comprehensive multi-string search strategies will be designed to retrieve studies from published literature. Search strings for the study population were informed by the search strategy used in NICE clinical Guideline CG166 [[5](#_ENREF_5)].

#### Data management

Bibliographic details and abstracts of all citations retrieved by the literature search will be downloaded into Microsoft Excel.

#### Selection process

Titles and abstracts identified by the search strategy will be independently assessed for possible eligibility by two reviewers. Those studies that do not meet eligibility criteria will be excluded. For those citations that could potentially meet the eligibility criteria, full texts will be retrieved, and eligibility criteria applied. Any discrepancies between the two reviewers will be resolved by a third reviewer. Reviewers will document reasons for exclusion and present the results in the form of a PRISMA flow diagram allowing full traceability of studies included and excluded.

#### Data collection

All data will be extracted in a consistent manner from studies meeting the review objectives. Data will be extracted by a single reviewer and checked by a second reviewer for accuracy. Reviewers will complete the data extraction for all fields and note where data in the relevant field is ‘not reported’ (NR) or ‘not applicable’ (NA).

All extracted data will be collated within tables in Microsoft Excel.

#### Data items

Data from studies identified as part of the SLR update will be added to the existing data extraction grid developed for the original SLR.

#### Quality Assessment

Each study that meets criteria for inclusion will be critically appraised by a single reviewer and reviewed by a second reviewer using the Cochrane Collaboration’s tool for assessing the risk of bias in line with NICE requirements [[6](#_ENREF_6)]. The Cochrane Collaboration’s risk of bias tool covers six domains, and assesses potential sources of bias within them:

- Selection bias:
- Performance bias
- Detection bias
- Attrition bias
- Reporting bias
- Other bias

# References

1. Moher D, Shamseer L, Clarke M, et al. Preferred reporting items for systematic review and meta-analysis protocols (PRISMA-P) 2015 statement. Systematic reviews. 2015;(4):1-1.

2. National Institute for Clinical Excellence.Tofacitinib for moderately to severely active ulcerative colitis. Technology appraisal guidance 547., 2018. Available from: <https://www.nice.org.uk/guidance/ta547> (Accessed 12/12/2018).

3. National Institute for Clinical Excellence.Vedolizumab for treating moderately to severely active ulcerative colitis. Technology appraisal guidance 342., 2015. Available from: <https://www.nice.org.uk/guidance/ta342> (Accessed 12/12/2018).

4. National Institute for Clinical Excellence.Infliximab, adalimumab and golimumab for treating moderately to severely active ulcerative colitis after the failure of conventional therapy. Technology appraisal guidance 329., 2015. Available from: <https://www.nice.org.uk/guidance/ta329> (Accessed 12/12/2018).

5. National Institute for Clinical Excellence.Ulcerative colitis: management. Clinical guideline 166., 2013. Available from: <https://www.nice.org.uk/guidance/cg166> (Accessed 12/12/2018).

6. Higgins JP, Green S. Cochrane handbook for systematic reviews of interventions: Wiley Online Library. 2008;(
