# Supplementary material for: Comparative efficacy of advanced treatments in biologic-naïve or biologic-experienced patients with ulcerative colitis: a systematic review and network meta-analysis
Source: Int J Clin Pharm. 2022 Dec 9;45(2):330–41. doi: 10.1007/s11096-022-01509-1 (PMC10147762; doi:10.1007/s11096-022-01509-1)
Supplement: Supplementary file 2 — Supplementary file2 (DOCX 374 KB) [file 11096_2022_1509_MOESM2_ESM.docx]

**Comparative efficacy of advanced treatments in biologic-naïve or biologic-experienced patients with ulcerative colitis: a systematic review and network meta-analysis – Supplementary Material 2**

## Systematic searching

**Supplementary table 1.** Search terms used in systematic searching.

*Embase (for Embase and MEDLINE^®^)*

| **#** | **Search terms** | **Number of hits May 2019** | **Number of hits May 2019- November 2020** |
| --- | --- | --- | --- |
|  | **Population** |  |  |
| 1 | ‘Ulcerative colitis’/exp OR ‘ulcerative colitis’/exp OR ‘Ulcerative proctitis’/exp OR ‘Proctosigmoiditis’/exp OR ‘Left-sided colitis’/exp OR ‘Pancolitis’/exp OR ‘Proctocolitis’/exp | 79,634 | 81,212 |
| 2 | (pancolitis or rectitis or proctocolitis or procto-colitis or colorectitis or rectocolitis or rectocolitis or recto-sigmoiditis or rectosigmoiditis or procto-sigmoiditis or proctosigmoiditis or proctitis):ti,ab | 7,289 | 8,122 |
| 3 | (coli*:ti,ab) AND ulcerat*:ti,ab | 59,326 | 67,348 |
| 4 | Inflammatory:ti,ab AND bowel:ti,ab AND disease:ti,ab | 69,219 | 80,840 |
| 5 | #1 OR #2 OR #3 OR #4 | 122,196 | 138,543 |
|  | **Biologics** |  |  |
| 6 | Infliximab:ti,ab OR Remicade:ti,ab | 23,308 | 26,383 |
| 7 | Golimumab:ti,ab OR Simponi:ti,ab | 3,117 | 3,727 |
| 8 | Apremilast:ti,ab OR Otezla:ti,ab | 924 | 1,346 |
| 9 | Adalimumab:ti,ab OR Humira:ti,ab OR Trudexa:ti,ab OR “ABP 501”:ti,ab OR BI695501:ti,ab OR CHS-1420:ti,ab OR GP2017:ti,ab OR M923:ti,ab OR PF-06410293:ti,ab | 15,701 | 18,413 |
| 10 | Vedolizumab:ti,ab OR Entyvio:ti,ab | 1,682 | 2,764 |
| 11 | #6 OR #7 OR #8 OR #9 OR #10 | 33,561 | 39,219 |
|  | **Targeted synthetic drugs** |  |  |
| 12 | Baricitinib:ti,ab OR Olumiant:ti,ab | 453 | 767 |
| 13 | Peficitinib:ti,ab OR ASP015K:ti,ab | 49 | 93 |
| 14 | Tofacitinib:ti,ab OR Xeljanz:ti,ab | 1,966 | 2,915 |
| 15 | Upadacitinib:ti,ab OR ABT-494:ti,ab | 117 | 308 |
| 16 | Filgotinib:ti,ab OR GLPG0634:ti,ab OR GS-6034:ti,ab | 156 | 257 |
| 17 | PF-06651600:ti,ab OR PF-06700841:ti,ab | 12 | 19 |
| 18 | TD-1473:ti,ab | 6 | 11 |
| 19 | #12 OR #12 OR #13 OR #14 OR #15 OR #16 OR #17 OR #18 | 2,574 | 3,962 |
|  | **Biosimilars** |  |  |
|  | **Infliximab biosimilars** |  |  |
| 20 | Inflectra:ti,ab OR Remsima:ti,ab OR Flixabi:ti,ab OR Renflexis:ti,ab OR SB2:ti,ab OR CT-P13:ti,ab OR PF-06438179:ti,ab OR PF-06438179:ti,ab OR ABP501:ti,ab OR “Infliximab BS”:ti,ab OR Infliximab-dyyb:ti,ab OR Infliximab-qbtx:ti,ab OR Infliximab-abda:ti,ab OR Ixifi:ti,ab | 955 | 1,270 |
|  | **Adalimumab biosimilars** |  |  |
| 21 | Amjevita:ti,ab OR ABP-501:ti,ab OR Cyltezo:ti,ab OR BI 695501:ti,ab OR SB5:ti,ab OR MSB11022:ti,ab OR Imraldi:ti,ab OR GP2017:ti,ab OR Adfrar:ti,ab OR Exemptia:ti,ab OR Amjevita:ti,ab OR Adalimumab-atto:ti,ab OR Adalimumab-adbm:ti,ab | 154 | 253 |
| 22 | #20 OR #21 | 1,090 | 1,486 |
|  | **Surgery** |  |  |
| 23 | ‘Colectomy’/exp OR ‘ileostomy’/exp OR ‘colonic pouches’/exp | 51,010 | 56,924 |
| 24 | Ileostomy:ti,ab OR ‘colonic pouches’:ti,ab OR ‘colon pouch’:ti,ab OR colectomy:ti,ab OR proctocolectomy:ti,ab OR procto-colectomy:ti,ab OR IPAA:ti,ab | 27,577 | 30,860 |
| 25 | (j:ti,ab OR s:ti,ab OR w:ti,ab,kw OR kock:ti,ab OR pelvic:ti,ab) AND pouch:ti,ab OR (((ileal:ti,ab OR ileoanal:ti,ab OR ileo) AND anal:ti,ab OR anal:ti,ab OR ileum:ti,ab OR anus:ti,ab OR rectal:ti,ab OR rectum:ti,ab) AND pouch*:ti,ab) OR (((ileal:ti,ab OR ileoanal:ti,ab OR ileo) AND anal:ti,ab OR anal:ti,ab OR ileum:ti,ab OR anus:ti,ab OR rectal:ti,ab OR rectum:ti,ab) AND reservoir*:ti,ab) OR ((ileal:ti,ab OR pouch;ti,ab OR ileoanal:ti,ab OR 'ileo anal':ti,ab OR anal:ti,ab OR ileum:ti,ab OR anus:ti,ab OR rectal:ti,ab OR rectum) AND anastomos*:ti,ab) | 23,453 | 25,580 |
| 26 | #23 OR #24 OR #25 | 72,479 | 80,083 |
|  | **Combine** |  |  |
| 27 | #11 OR #19 OR #22 OR #26 | 106,638 | 120,820 |
| 28 | #5 AND #27 | 21,261 | 24,681 |
|  | **Randomised controlled trials (RCTs) search terms** |  |  |
| 29 | 'randomized controlled trial (topic)'/exp OR 'randomized controlled trial (topic)' | 158,635 | 189,094 |
| 30 | 'random allocation' | 2,046 | 2,265 |
| 31 | 'double blind procedure' | 160,041 | 177,556 |
| 32 | 'single blind procedure' | 34,749 | 40,548 |
| 33 | 'crossover procedure' | 58,807 | 64,621 |
| 34 | 'prospective study' | 575,810 | 697,701 |
| 35 | 'controlled clinical trial' | 446,372 | 454,627 |
| 36 | 'clinical trial' | 1,486,912 | 1,595,309 |
| 37 | 'clinical study' | 3,566,572 | 4,115,320 |
| 38 | 'phase 1 clinical trial (topic)' | 20,870 | 23,428 |
| 39 | 'phase 2 clinical trial (topic)' | 32,566 | 36,603 |
| 40 | 'phase 3 clinical trial (topic)' | 33,669 | 38,609 |
| 41 | 'phase 4 clinical trial (topic)' | 1,476 | 1,675 |
| 42 | 'comparative study' | 873,505 | 948,053 |
| 43 | 'multicenter study' | 251,172 | 307,578 |
| 44 | (Clinical:ti,ab OR control:ti,ab OR controlled:ti,ab) AND (study:ti,ab OR trial:ti,ab) | 3,585,478 | 4,089,605 |
| 45 | (Singl*:ti,ab OR doubl*:ti,ab OR treb*:ti,ab OR tripl*:ti,ab) AND (blind*:ti,ab OR mask*:ti,ab) | 253,918 | 278,757 |
| 46 | Random*:ti,ab AND (assign*:ti,ab OR allocate*:ti,ab OR group:ti,ab OR grouped:ti,ab OR patients:ti,ab OR study:ti,ab OR trial:ti,ab OR distribut*:ti,ab) | 1,240,547 | 1,409,600 |
| 47 | Crossover:ti,ab AND (design:ti,ab OR study:ti,ab OR trial:ti,ab) | 59,052 | 90,307 |
| 48 | Prospective:ti,ab OR randomized:ti,ab OR randomised:ti,ab OR trial:ti,ab OR phase:ti,ab | 2,738,045 | 3,085,274 |
| 49 | Placebo*:ti,ab | 288,102 | 315,117 |
| 50 | #29 OR #30 OR #31 OR #32 OR #33 OR #34 OR #35 OR #36 OR #37 OR #38 OR #39 OR #40 OR #41 OR #42 OR #43 OR #44 OR #45 OR #46 OR #47 OR #48 OR #49 | 9,013,170 | 10,106,957 |
|  | **Exclusion Terms** |  |  |
| 51 | 'longitudinal study' | 144,896 | 167,287 |
| 52 | 'case control study' | 185,045 | 208,942 |
| 53 | 'cross-sectional study' | 332,747 | 410,720 |
| 54 | 'observational study' | 192,941 | 241,836 |
| 55 | 'longitudinal study' | 144,896 | 167,287 |
| 56 | 'retrospective study' | 803,813 | 1,021,401 |
| 57 | 'prospective study' | 575,810 | 697,701 |
| 58 | Cohort:ti,ab AND (study:ti,ab OR studies:ti,ab OR analys*:ti,ab) | 694,664 | 848,598 |
| 59 | (‘follow up’:ti,ab OR observational:ti,ab OR uncontrolled:ti,ab OR ‘non randomi*ed’:ti,ab OR epidemiologic*:ti,ab) AND (study:ti,ab OR studies:ti,ab) | 1,266,350 | 1,461,748 |
| 60 | (longitudinal:ti,ab OR retrospective:ti,ab OR prospective:ti,ab) AND (study:ti,ab OR studies:ti,ab OR review:ti,ab OR analys*:ti,ab or cohort*:ti,ab) | 1,596,764 | 1,867,623 |
| 61 | Review:it | 2,476,433 | 2,667,387 |
| 62 | Editorial:it | 595,466 | 664,210 |
| 63 | Letter:it | 1,054,935 | 1,129,278 |
| 64 | ‘Comment’ | 66,112 | 734,78 |
| 65 | ‘Case not*’:it OR ‘Case repor*’:it OR survey:it | 351,764 | 358,330 |
| 66 | [animals]/lim NOT [humans]/lim | 5,576,783 | 5,868,170 |
| 67 | #51 OR #52 OR #53 OR #54 OR #55 OR #56 OR #57 OR #58 OR #59 OR #60 OR #61 OR #62 OR #63 OR #64 OR #65 OR #66 | 13,133,460 | 14,320,988 |
|  | **Combine** |  |  |
| 68 | #28 AND #50 | 11,337 | 13,738 |
| 69 | #68 NOT #67 | 4,299 | 4,970 |
|  | **Limits** |  |  |
| 70 | #69 AND [english]/lim | 4,038 | 4,701 |
| 71 | #70 AND ([article]/lim OR [article in press]/lim OR [conference abstract]/lim OR [conference paper]/lim) | **3,930** | 4,587 |
|  | #71 AND [1-4-2019]/sd NOT [1-1-2021]/sd |  | 751 |

*Pubmed (for MEDLINE and MEDLINE In-Process)*

| **#** | **Search terms** | **Number of hits May 2019** | **Number of hits May 2019- November 2020** |
| --- | --- | --- | --- |
|  | **Population** |  |  |
| 1 | colitis, ulcerative[MeSH Terms] OR proctitis[MeSH Terms] OR “inflammatory bowel diseases”[MeSH Terms] | 77,953 | 84,097 |
| 2 | pancolitis*[Title/Abstract] or rectitis*[Title/Abstract] or proctocolitis*[Title/Abstract] or procto-colitis*[Title/Abstract] or colorectitis*[Title/Abstract] or rectocolitis*[Title/Abstract] or rectocolitis*[Title/Abstract] or recto-sigmoiditis*[Title/Abstract] or rectosigmoiditis*[Title/Abstract] or procto-sigmoiditis*[Title/Abstract] or proctosigmoiditis*[Title/Abstract] or proctitis*[Title/Abstract] | 4,403 | 2,142 |
| 3 | ulcerative[Title/Abstract] AND colitis[Title/Abstract] | 37,737 | 41,648 |
| 4 | inflammatory[Title/Abstract] AND bowel[Title/Abstract] AND disease[Title/Abstract] | 43,840 | 50,283 |
| 5 | #1 OR #2 OR #3 OR #4 | 101,118 | 110,254 |
|  | **Biologics** |  |  |
| 6 | (Infliximab[Title/Abstract]) OR Remicade[Title/Abstract] | 11,382 | 12,655 |
| 7 | (Golimumab[Title/Abstract]) OR Simponi[Title/Abstract] | 937 | 1,144 |
| 8 | Apremilast[Title/Abstract] OR Otezla[Title/Abstract] | 438 | 651 |
| 9 | ((Adalimumab[Title/Abstract]) OR Humira[Title/Abstract]) OR Trudexa[Title/Abstract]) OR ABP 501[Title/Abstract]) OR BI695501[Title/Abstract]) OR CHS-1420[Title/Abstract]) OR GP2017[Title/Abstract]) OR M923[Title/Abstract]) OR PF-06410293[Title/Abstract] | 6,137 | 7,216 |
| 10 | (Vedolizumab[Title/Abstract]) OR Entyvio[Title/Abstract] | 642 | 960 |
| 11 | #6 OR #7 OR #8 OR #9 OR #10 | 15,620 | 17,959 |
|  | **Targeted synthetic drugs** |  |  |
| 12 | (Baricitinib[Title/Abstract]) OR Olumiant[Title/Abstract] | 168 | 383 |
| 13 | (Peficitinib[Title/Abstract]) OR ASP015K[Title/Abstract] | 32 | 63 |
| 14 | (Tofacitinib[Title/Abstract]) OR Xeljanz[Title/Abstract] | 919 | 1,403 |
| 15 | (Upadacitinib[Title/Abstract]) OR ABT-494[Title/Abstract] | 33 | 124 |
| 16 | (Filgotinib[Title/Abstract]) OR (GLPG0634[Title/Abstract]) OR (GS-6034[Title/Abstract]) | 52 | 103 |
| 17 | (PF-06651600[Title/Abstract]) OR PF-06700841[Title/Abstract] | 7 | 14 |
| 18 | (TD-1473[Title/Abstract]) | 1 | 3 |
| 19 | #12 OR #13 OR #14 OR #15 OR #16 OR #17 OR #18 | 1,090 | 1,796 |
|  | **Biosimilars** |  |  |
|  | **Infliximab biosimilars** |  |  |
| 20 | ((Inflectra[Title/Abstract]) OR Remsima[Title/Abstract]) OR Flixabi[Title/Abstract]) OR Renflexis[Title/Abstract]) OR SB2[Title/Abstract]) OR CT-P13[Title/Abstract]) OR PF-06438179[Title/Abstract]) OR PF-06438179[Title/Abstract]) OR ABP501[Title/Abstract]) OR “Infliximab BS”[Title/Abstract]) OR Infliximab-dyyb[Title/Abstract]) OR Infliximab-qbtx[Title/Abstract]) OR Infliximab-abda[Title/Abstract]) OR Ixifi[Title/Abstract]) | 534 | 752 |
|  | **Adalimumab biosimilars** |  |  |
| 21 | ((Amjevita[Title/Abstract]) OR ABP-501[Title/Abstract]) OR Cyltezo[Title/Abstract]) OR BI 695501[Title/Abstract]) OR SB5[Title/Abstract] OR MSB11022 [Title/Abstract]) OR Imraldi[Title/Abstract]) OR GP2017[Title/Abstract]) OR Adfrar[Title/Abstract]) OR Exemptia[Title/Abstract]) OR Amjevita[Title/Abstract]) OR Adalimumab-atto[Title/Abstract]) OR Adalimumab-adbm[Title/Abstract]) | 125 | 229 |
| 22 | #20 OR #21 | 642 | 950 |
|  | **Surgery** |  |  |
| 23 | Colectomy[MeSH Terms] OR ileostomy[MeSH Terms] OR “colonic pouches”[MeSH Terms] | 24,899 | 26,474 |
| 24 | Ileostomy[Title/Abstract] OR “colonic pouches”[Title/Abstract] OR “colon pouch”[Title/Abstract] OR colectomy[Title/Abstract] OR proctocolectomy[Title/Abstract] OR procto-colectomy[Title/Abstract] OR IPAA[Title/Abstract] | 17,899 | 19,514 |
| 25 | (((J[Title/Abstract] OR S[Title/Abstract] OR W[Title/Abstract] or kock[Title/Abstract] OR pelvic[Title/Abstract]) AND pouch[Title/Abstract]) OR ((ileal[Title/Abstract] or ileoanal[Title/Abstract] OR ileo anal[Title/Abstract] OR anal[Title/Abstract] OR ileum[Title/Abstract] OR anus[Title/Abstract] OR rectal[Title/Abstract] OR rectum[Title/Abstract]) AND pouch*[Title/Abstract]) OR ((ileal[Title/Abstract] OR ileoanal[Title/Abstract] OR ileo anal[Title/Abstract] OR anal[Title/Abstract] OR ileum[Title/Abstract] OR anus[Title/Abstract] OR rectal[Title/Abstract] OR rectum[Title/Abstract]) AND reservoir*[Title/Abstract]) OR ((ileal[Title/Abstract] OR pouch[Title/Abstract] OR ileoanal[Title/Abstract] OR ileo-anal[Title/Abstract] OR anal[Title/Abstract] OR ileum[Title/Abstract] OR anus[Title/Abstract] OR rectal[Title/Abstract] OR rectum) AND anastomos*[Title/Abstract])) | 15,360 | 18,029 |
| 26 | #23 OR #24 OR #25 | 41,511 | 46,220 |
|  | **Combine** |  |  |
| 27 | #11 OR #19 OR #22 OR #26 | 57,808 | 65,575 |
| 28 | #5 AND #27 | 14,297 | 15,824 |
|  | **Randomised controlled trials (RCTs) search terms** |  |  |
| 29 | Randomized-Controlled-Trials/ | 638,545 | 698,571 |
| 30 | Random-Allocation/ | 102,196 | 107,714 |
| 31 | Double-Blind-Method/ | 154,221 | 163,529 |
| 32 | Single-Blind-Method/ | 44,816 | 43,415 |
| 33 | Cross-Over-Studies/ | 145,004 | 136,639 |
| 34 | Prospective-Studies/ | 615,378 | 678,985 |
| 35 | Placebos/ | 35,908 | 233,486 |
| 36 | Clinical trial/ | 1,127,604 | 1,209,882 |
| 37 | Randomized controlled trial[Publication Type] OR Clinical-trial[publication type] OR controlled-clinical-trial[publication type] | 825,622 | 873,787 |
| 38 | Clinical trial[Publication Type] | 825,622 | 873,787 |
| 39 | Clinical trial, phase i[Publication Type] | 18,893 | 20,959 |
| 40 | Clinical trial, phase ii[Publication Type] | 30,534 | 33,661 |
| 41 | Clinical trial, phase iii[Publication Type] | 15,011 | 17,431 |
| 42 | Clinical trial, phase iv[Publication Type] | 1,706 | 1,986 |
| 43 | Comparative study [publication type] | 1,829,137 | 1,874,387 |
| 44 | Multicenter study [publication type] | 249,876 | 282,066 |
| 45 | (Clinical[Title/Abstract] OR control[Title/Abstract] OR controlled[Title/Abstract]) AND (study[Title/Abstract] OR trial[Title/Abstract]) | 2,502,485 | 2,825,837 |
| 46 | (Singl*[Title/Abstract] OR doubl*[Title/Abstract] OR treb*[Title/Abstract] OR tripl*[Title/Abstract]) AND (blind*[Title/Abstract] OR mask*[Title/Abstract]) | 183,335 | 197,826 |
| 47 | Random*[Title/Abstract] AND (assign*[Title/Abstract] OR allocate*[Title/Abstract] OR group[Title/Abstract] OR grouped[Title/Abstract] OR patients[Title/Abstract] OR study[Title/Abstract] OR trial[Title/Abstract] OR distribut*[Title/Abstract]) | 899,643 | 1,010,583 |
| 48 | Crossover[Title/Abstract] AND (design[Title/Abstract] OR study[Title/Abstract] OR trial[Title/Abstract]) | 47,319 | 70,505 |
| 49 | Prospective[Title/Abstract] OR randomized[Title/Abstract] OR randomised[Title/Abstract] OR trial[Title/Abstract] OR phase[Title/Abstract] | 2,056,081 | 2,276,600 |
| 50 | Placebo*[Title/Abstract] | 204,702 | 219,908 |
| 51 | #29 OR #30 OR #31 OR #32 OR #33 OR #34 OR #35 OR #36 OR #37 OR #38 OR #39 OR #40 OR #41 OR #42 OR #43 OR #44 OR #45 OR #46 OR #47 OR #48 OR #49 or #50 | 6,033,579 | 6,563,199 |
|  | **Exclusion terms** |  |  |
| 52 | Epidemiologic studies/ | 2,333,979 | 2,589,835 |
| 53 | Case control studies/ | 1,055,908 | 1,186,340 |
| 54 | Cohort studies/ | 1,923,677 | 2,131,479 |
| 55 | Cross-sectional studies/ | 334,864 | 389,356 |
| 56 | Observational study[publication type] | 61,428 | 87,290 |
| 57 | Cohort[Title/Abstract] AND (study[Title/Abstract] OR studies[Title/Abstract] OR analys*[Title/Abstract]) | 416,059 | 503,585 |
| 58 | (“follow up”[Title/Abstract] OR observational[Title/Abstract] OR uncontrolled[Title/Abstract] OR “non randomized”[Title/Abstract] OR “non randomised”[Title/Abstract]) AND (study[Title/Abstract] OR studies[Title/Abstract]) | 687,507 | 790,081 |
| 59 | (longitudinal[Title/Abstract] OR retrospective[Title/Abstract] OR prospective[Title/Abstract]) AND (study[Title/Abstract] OR studies[Title/Abstract] OR review[Title/Abstract] OR analys*[Title/Abstract] or cohort*[Title/Abstract]) | 1,050,885 | 1,207,645 |
| 60 | cross sectional[Title/Abstract] | 308,727 | 368,405 |
| 61 | Review [publication type] | 2,513,782 | 2,718,745 |
| 62 | Editorial [publication type] | 488,967 | 545,295 |
| 63 | Letter [publication type] | 1,025,143 | 1,105,740 |
| 64 | Comment [publication type] | 769,698 | 875,751 |
| 65 | ((Case not*[Title/Abstract]) OR Case repor*[Title/Abstract]) OR survey[Title/Abstract] | 850,445 | 949,401 |
| 66 | (animals[MeSH Terms]) NOT humans[MeSH Terms] | 4,577,949 | 4,752,218 |
| 67 | #52 OR #53 OR #54 OR #55 OR #56 OR #57 OR #58 OR #59 OR #60 OR #61 OR #62 OR #63 OR #64 OR #65 OR #66 | 11,989,514 | 12,952,892 |
|  | **Combine** |  |  |
| 68 | #28 AND #51 | 4,871 | 5,573 |
| 69 | #68 NOT #67 | 1,171 | 1,328 |
| 70 | #69 AND English [Language] | 1,057 | 1,213 |
| 71 | #69 AND English [Language] and 2019/4/1 - 2020/12/31 | - | 185 |

*Cochrane Library*

| **#** | **Search terms** | **Number of hits May 2019** | **Number of hits May 2019- November 2020** |
| --- | --- | --- | --- |
|  | **Population** |  |  |
| 1 | “Inflammatory Bowel Disease”[MeSH descriptor] | 2,868 | 3,325 |
| 2 | Colitis, Ulcerative[MeSH descriptor] | 1,361 | 1,585 |
| 3 | Proctitis[MeSH descriptor] | 131 | 139 |
| 4 | (ulcer* near/2 colitis):ti,ab,kw | 4,337 | 4,846 |
| 5 | (inflamm* near/2 (colon* or bowel)):ti,ab,kw | 3,056 | 3,245 |
| 6 | (pancolitis or rectitis or proctocolitis or procto-colitis or colorectitis or rectocolitis or rectocolitis or recto-sigmoiditis or rectosigmoiditis or procto-sigmoiditis or proctosigmoiditis or proctitis):ti,ab,kw | 613 | 623 |
| 7 | ((total or sub-total or subtotal or extensive or left-sided or universal) near colitis):ti,ab,kw | 234 | 237 |
| 8 | #1 OR #2 OR #3 OR #4 OR #5 OR #6 OR #7 | 7,536 | 8,348 |
|  | **Biologics** |  |  |
| 9 | Infliximab:ti,ab,kw OR Remicade:ti,ab,kw | 2,338 | 2,326 |
| 10 | Golimumab:ti,ab,kw OR Simponi:ti,ab,kw | 632 | 671 |
| 11 | Apremilast:ti,ab,kw OR Otezla:ti,ab,kw | 357 | 427 |
| 12 | Adalimumab:ti,ab,kw OR Humira:ti,ab,kw OR Trudexa:ti,ab,kw OR “ABP 501”:ti,ab,kw OR BI695501:ti,ab,kw OR CHS-1420:ti,ab,kw OR GP2017:ti,ab,kw OR M923:ti,ab,kw OR PF-06410293:ti,ab,kw | 2,712 | 3,028 |
| 13 | Vedolizumab:ti,ab,kw OR Entyvio:ti,ab,kw | 311 | 393 |
| 14 | #9 OR #10 OR #11 OR #12 OR #13 | 5,397 | 5,970 |
|  | **Targeted synthetic drugs** |  |  |
| 15 | Baricitinib:ti,ab,kw OR Olumiant:ti,ab,kw | 267 | 366 |
| 16 | Peficitinib:ti,ab,kw OR ASP015K:ti,ab,kw | 31 | 44 |
| 17 | Tofacitinib:ti,ab,kw OR Xeljanz:ti,ab,kw | 544 | 733 |
| 18 | Upadacitinib:ti,ab,kw OR ABT-494:ti,ab,kw | 119 | 233 |
| 19 | Filgotinib:ti,ab,kw OR GLPG0634:ti,ab,kw OR GS-6034:ti,ab,kw | 113 | 157 |
| 20 | PF-06651600:ti,ab,kw OR PF-06700841:ti,ab,kw | 23 | 46 |
| 21 | TD-1473:ti,ab,kw | 8 | 14 |
| 22 | #15 OR #15 OR #16 OR #17 OR #18 OR #19 OR #20 OR #21 | 1,090 | 1,577 |
|  | **Biosimilars** |  |  |
|  | **Infliximab biosimilars** |  |  |
| 23 | Inflectra:ti,ab,kw OR Remsima:ti,ab,kw OR Flixabi:ti,ab,kw OR Renflexis:ti,ab,kw OR SB2:ti,ab,kw OR CT-P13:ti,ab,kw OR PF-06438179:ti,ab,kw OR PF-06438179:ti,ab,kw OR ABP501:ti,ab,kw OR “Infliximab BS”:ti,ab,kw OR Infliximab-dyyb:ti,ab,kw OR Infliximab-qbtx:ti,ab,kw OR Infliximab-abda:ti,ab,kw OR Ixifi:ti,ab,kw | 162 | 194 |
|  | **Adalimumab biosimilars** |  |  |
| 24 | Amjevita:ti,ab,kw OR ABP-501:ti,ab OR Cyltezo:ti,ab,kw OR BI 695501:ti,ab,kw OR SB5:ti,ab,kw OR MSB11022:ti,ab,kw OR Imraldi:ti,ab,kw OR GP2017:ti,ab,kw OR Adfrar:ti,ab,kw OR Exemptia:ti,ab,kw OR Amjevita:ti,ab,kw OR Adalimumab-atto:ti,ab,kw OR Adalimumab-adbm:ti,ab,kw | 111 | 152 |
| 25 | #23 OR #24 | 269 | 338 |
|  | **Surgery** |  |  |
| 26 | Colectomy [MeSH descriptor] | 676 | 722 |
| 27 | ileostomy [MeSH descriptor] | 187 | 198 |
| 28 | colonic pouches [MeSH descriptor] | 46 | 51 |
| 29 | Ileostomy:ti,ab OR ‘colonic pouches’:ti,ab OR ‘colon pouch’:ti,ab OR colectomy:ti,ab OR proctocolectomy:ti,ab OR procto-colectomy:ti,ab OR IPAA:ti,ab | 1,600 | 1,768 |
| 30 | ((J:ti,ab,kw OR S:ti,ab,kw OR W:ti,ab,kw or kock:ti,ab,kw or pelvic:ti,ab,kw) AND pouch:ti,ab,kw) OR ((ileal:ti,ab,kw or ileoanal:ti,ab,kw OR ileo anal:ti,ab,kw OR anal:ti,ab,kw OR ileum:ti,ab,kw OR anus:ti,ab,kw OR rectal:ti,ab,kw OR rectum:ti,ab,kw) AND pouch*:ti,ab,kw) OR ((ileal:ti,ab,kw OR ileoanal:ti,ab,kw OR ileo anal:ti,ab,kw OR anal:ti,ab,kw OR ileum:ti,ab,kw OR anus:ti,ab,kw OR rectal:ti,ab,kw OR rectum:ti,ab,kw) AND reservoir*:ti,ab,kw) OR ((ileal:ti,ab,kw OR pouch:ti,ab,kw OR ileoanal:ti,ab,kw OR ileo-anal:ti,ab,kw OR anal:ti,ab,kw OR ileum:ti,ab,kw OR anus:ti,ab,kw OR rectal:ti,ab,kw OR rectum) AND anastomos*:ti,ab,kw) | 1,297 | 1,441 |
| 31 | #26 OR #27 OR #28 OR #29 OR #30 | 2,874 | 3,180 |
|  | **Combine** |  |  |
| 32 | #14 OR #22 OR #25 OR #31 | 9,085 | 10,390 |
| 33 | #8 AND #32 | 1,603 | 1,816 |
|  | **Study Type** |  |  |
| 34 | Controlled clinical trial:pt | 455,795 | 471,313 |
| 35 | Randomized controlled trial:pt | 472,157 | 499,031 |
| 36 | Comparative study:pt | 164,682 | 167,902 |
| 37 | Clinical study:pt | 188,203 | 193,888 |
| 38 | Clinical trial:pt | 455,795 | 471,313 |
| 39 | Multicenter study:pt | 82,884 | 88,811 |
| 40 | (prospective OR randomised OR randomized OR trial OR phase):ti,ab,kw | 978,085 | 1,088,122 |
| 41 | #34 OR #35 OR #36 OR #37 OR #38 OR #39 OR #40 | 1,160,481 | 1,268,092 |
| 42 | Observational study:pt | 2,519 | 2,708 |
| 43 | Review:pt | 26,151 | 17,879 |
| 44 | Editorial:pt | 2,605 | 2,745 |
| 45 | Letter:pt | 10,896 | 12,006 |
| 46 | Comment:pt | 1,870 | 1,879 |
| 47 | Guideline:pt | 33 | 31 |
| 48 | (observational OR longitudinal OR retrospective):ti,ab,kw | 61,423 | 61,644 |
| 49 | (“Case not*” OR “Case repor*”):ti,ab,kw | 52 | 51 |
| 50 | (Biomarker OR epigenetic OR in-vitro):ti,ab,kw | 30,862 | 32,708 |
| 51 | (pharmacokinetics OR pharmacokinetic)ti,ab,kw | 79 | 86 |
| 52 | #42 OR #43 OR #44 OR #45 OR #46 OR #47 OR #48 OR #49 OR #50 OR #51 | 128,695 | 124,455 |
|  | **Combine** |  |  |
| 53 | #33 AND #41 | 1,387 | 1,567 |
| 54 | #53 NOT #52 | 1,114 | - |
|  | **Limits** |  |  |
| 55 | #54 Limit to Trials | 1,090 | - |
| 54b | #53 NOT #52 with Cochrane Library publication date Between Apr 2019 and Dec 2020, in Trials | - | 301 |

**Supplementary table 2.** Ustekinumab additional search terms.

*Embase (for Embase and MEDLINE)*

| **#** | **Terms** | **Number of hits November 2020** |
| --- | --- | --- |
|  | **Population** |  |
| 1 | 'ulcerative colitis'/exp OR 'ulcerative proctitis'/exp OR 'proctosigmoiditis'/exp OR 'left-sided colitis' OR 'pancolitis'/exp OR 'proctocolitis'/exp | 81,383 |
| 2 | pancolitis:ti,ab OR rectitis:ti,ab OR proctocolitis:ti,ab OR 'procto colitis':ti,ab OR colorectitis:ti,ab OR rectocolitis:ti,ab OR 'recto sigmoiditis':ti,ab OR rectosigmoiditis:ti,ab OR 'procto sigmoiditis':ti,ab OR proctosigmoiditis:ti,ab OR proctitis:ti,ab | 8,136 |
| 3 | coli*:ti,ab AND ulcerat*:ti,ab | 67,494 |
| 4 | inflammatory:ti,ab AND bowel:ti,ab AND disease:ti,ab | 81,126 |
| 5 | #1 OR #2 OR #3 OR #4 | 138,922 |
|  | **Intervention** |  |
| 6 | ustekinumab:ti,ab OR stelara:ti,ab | 4,253 |
| 7 | #5 AND #6 | 651 |
|  | **Randomised controlled trials (RCT) search terms** |  |
| 8 | 'randomized controlled trial (topic)'/exp OR 'randomized controlled trial (topic)' | 189,963 |
| 9 | 'random allocation' | 2,278 |
| 10 | 'double blind procedure' | 178,024 |
| 11 | 'single blind procedure' | 40,689 |
| 12 | 'crossover procedure' | 64,754 |
| 13 | 'prospective study' | 700,875 |
| 14 | 'controlled clinical trial' | 454,901 |
| 15 | 'clinical trial' | 1,598,294 |
| 16 | 'clinical study' | 4,129,966 |
| 17 | 'phase 1 clinical trial (topic)' | 23,499 |
| 18 | 'phase 2 clinical trial (topic)' | 36,721 |
| 19 | 'phase 3 clinical trial (topic)' | 38,738 |
| 20 | 'phase 4 clinical trial (topic)' | 1,680 |
| 21 | 'comparative study' | 949,864 |
| 22 | 'multicenter study' | 309,080 |
| 23 | (clinical:ti,ab OR control:ti,ab OR controlled:ti,ab) AND (study:ti,ab OR trial:ti,ab) | 4,103,879 |
| 24 | (singl*:ti,ab OR doubl*:ti,ab OR treb*:ti,ab OR tripl*:ti,ab) AND (blind*:ti,ab OR mask*:ti,ab) | 279,454 |
| 25 | random*:ti,ab AND (assign*:ti,ab OR allocate*:ti,ab OR group:ti,ab OR grouped:ti,ab OR patients:ti,ab OR study:ti,ab OR trial:ti,ab OR distribut*:ti,ab) | 1,414,274 |
| 26 | crossover:ti,ab AND (design:ti,ab OR study:ti,ab OR trial:ti,ab) | 90,497 |
| 27 | prospective:ti,ab OR randomized:ti,ab OR randomised:ti,ab OR trial:ti,ab OR phase:ti,ab | 3,094,281 |
| 28 | placebo*:ti,ab | 31,5915 |
| 29 | #8 OR #9 OR #10 OR #11 OR #12 OR #13 OR #14 OR #15 OR #16 OR #17 OR #18 OR #19 OR #20 OR #21 OR #22 OR #23 OR #24 OR #25 OR #26 OR #27 OR #28 | 10,136,494 |
|  | **Exclusion terms** |  |
| 30 | 'longitudinal study' | 167,996 |
| 31 | 'case control study' | 209,726 |
| 32 | 'cross-sectional study' | 413,321 |
| 33 | 'observational study' | 243,254 |
| 34 | 'longitudinal study' | 167,996 |
| 35 | 'retrospective study' | 1,027,329 |
| 36 | 'prospective study' | 700,875 |
| 37 | cohort:ti,ab AND (study:ti,ab OR studies:ti,ab OR analys*:ti,ab) | 852,975 |
| 38 | ('follow up':ti,ab OR observational:ti,ab OR uncontrolled:ti,ab OR 'non randomi*ed':ti,ab OR epidemiologic*:ti,ab) AND (study:ti,ab OR studies:ti,ab) | 1,467,110 |
| 39 | (longitudinal:ti,ab OR retrospective:ti,ab OR prospective:ti,ab) AND (study:ti,ab OR studies:ti,ab OR review:ti,ab OR analys*:ti,ab OR cohort*:ti,ab) | 1,874,965 |
| 40 | review:it | 2,673,828 |
| 41 | editorial:it | 665,495 |
| 42 | letter:it | 1,131,597 |
| 43 | 'comment' | 73,661 |
| 44 | 'case not*':it OR 'case repor*':it OR survey:it | 358,489 |
| 45 | [animals]/lim NOT [humans]/lim | 5,875,173 |
| 46 | #30 OR #31 OR #32 OR #33 OR #34 OR #35 OR #36 OR #37 OR #38 OR #39 OR #40 OR #41 OR #42 OR #43 OR #44 OR #45 | 14,353,752 |
|  | **Combine** |  |
| 47 | #7 AND #29 | 476 |
| 48 | #47 NOT #46 | 182 |
|  | **Limits** |  |
| 49 | #48 AND [english]/lim | 178 |
| 50 | #49 AND ([article]/lim OR [article in press]/lim OR [conference abstract]/lim OR [conference paper]/lim) | 178 |

*Pubmed (for MEDLINE and MEDLINE In-Process)*

| **#** | **Search terms** | **Number of hits November 2020** |
| --- | --- | --- |
|  | **Population** |  |
| 1 | colitis, ulcerative[MeSH Terms] OR proctitis[MeSH Terms] OR "inflammatory bowel diseases"[MeSH Terms] | 84,241 |
| 2 | pancolitis*[Title/Abstract] or rectitis*[Title/Abstract] or proctocolitis*[Title/Abstract] or procto-colitis*[Title/Abstract] or colorectitis*[Title/Abstract] or rectocolitis*[Title/Abstract] or rectocolitis*[Title/Abstract] | 2,145 |
| 3 | ulcerative[Title/Abstract] AND colitis[Title/Abstract] | 41,748 |
| 4 | inflammatory[Title/Abstract] AND bowel[Title/Abstract] AND disease[Title/Abstract] | 50,428 |
| 5 | #1 OR #2 OR #3 OR #4 | 110,474 |
|  | **Intervention** |  |
| 6 | (Ustekinumab[Title/Abstract]) OR (Stelara[Title/Abstract]) | 1,932 |
| 7 | #5 and #6 | 405 |
|  | **Randomised controlled trials (RCTs) search terms** |  |
| 8 | Randomized-Controlled-Trials/ | 699,858 |
| 9 | Random-Allocation/ | 107,802 |
| 10 | Double-Blind-Method/ | 163,729 |
| 11 | Single-Blind-Method/ | 43,492 |
| 12 | Cross-Over-Studies/ | 137,078 |
| 13 | Prospective-Studies/ | 680,567 |
| 14 | Placebos/ | 233,808 |
| 15 | Clinical trial/ | 1,211,577 |
| 16 | Randomized controlled trial[Publication Type] OR Clinical-trial[publication type] OR controlled-clinical-trial[publication type] | 874,839 |
| 17 | Clinical trial[Publication Type] | 874,839 |
| 18 | Clinical trial, phase i[Publication Type] | 20,991 |
| 19 | Clinical trial, phase ii[Publication Type] | 33,726 |
| 20 | Clinical trial, phase iii[Publication Type] | 17,482 |
| 21 | Clinical trial, phase iv[Publication Type] | 1,994 |
| 22 | Comparative study [publication type] | 1,875,349 |
| 23 | Multicenter study [publication type] | 282,820 |
| 24 | (Clinical[Title/Abstract] OR control[Title/Abstract] OR controlled[Title/Abstract]) AND (study[Title/Abstract] OR trial[Title/Abstract]) | 2,833,520 |
| 25 | (Singl*[Title/Abstract] OR doubl*[Title/Abstract] OR treb*[Title/Abstract] OR tripl*[Title/Abstract]) AND (blind*[Title/Abstract] OR mask*[Title/Abstract]) | 198,140 |
| 26 | Random*[Title/Abstract] AND (assign*[Title/Abstract] OR allocate*[Title/Abstract] OR group[Title/Abstract] OR grouped[Title/Abstract] OR patients[Title/Abstract] OR study[Title/Abstract] OR trial[Title/Abstract] OR distribut*[Title/Abstract]) | 1,013,245 |
| 27 | Crossover[Title/Abstract] AND (design[Title/Abstract] OR study[Title/Abstract] OR trial[Title/Abstract]) | 70,595 |
| 28 | Prospective[Title/Abstract] OR randomized[Title/Abstract] OR randomised[Title/Abstract] OR trial[Title/Abstract] OR phase[Title/Abstract] | 2,282,872 |
| 29 | Placebo*[Title/Abstract] | 220,225 |
| 30 | #6 OR #7 OR #8 OR #9 OR #10 OR #11 OR #12 OR #13 OR #14 OR #15 OR #16 OR #17 OR #18 OR #19 OR #20 OR #21 OR #22 OR #23 OR #24 OR #25 OR #26 OR #27 OR #28 OR #29 | 6,577,576 |
|  | **Combine** |  |
| 31 | #7 AND #30 | 405 |
|  | **Exclusion terms** |  |
| 32 | Epidemiologic studies/ | 2,596,092 |
| 33 | Case control studies/ | 1,189,690 |
| 34 | Cohort studies/ | 2,136,613 |
| 35 | Cross-sectional studies/ | 390,705 |
| 36 | Observational study[publication type] | 87,953 |
| 37 | Cohort[Title/Abstract] AND (study[Title/Abstract] OR studies[Title/Abstract] OR analys*[Title/Abstract]) | 505,844 |
| 38 | ("follow up"[Title/Abstract] OR observational[Title/Abstract] OR uncontrolled[Title/Abstract] OR "non randomized"[Title/Abstract] OR "non randomised"[Title/Abstract]) AND (study[Title/Abstract] OR studies[Title/Abstract]) | 792,693 |
| 39 | (longitudinal[Title/Abstract] OR retrospective[Title/Abstract] OR prospective[Title/Abstract]) AND (study[Title/Abstract] OR studies[Title/Abstract] OR review[Title/Abstract] OR analys*[Title/Abstract] or cohort*[Title/Abstract]) | 1,212,163 |
| 40 | cross sectional[Title/Abstract] | 370,008 |
| 41 | Review [publication type] | 2,722,903 |
| 42 | Editorial [publication type] | 546,325 |
| 43 | Letter [publication type] | 1,107,171 |
| 44 | Comment [publication type] | 877,277 |
| 45 | ((Case not*[Title/Abstract]) OR Case repor*[Title/Abstract]) OR survey[Title/Abstract] | 952,088 |
| 46 | (animals[MeSH Terms]) NOT humans[MeSH Terms] | 4,756,281 |
| 47 | #32 OR #33 OR #34 OR #35 OR #36 OR #37 OR #38 OR #39 OR #40 OR #41 OR #42 OR #43 OR #44 OR #45 OR #46 | 12,974,406 |
|  | **Combine** |  |
| 48 | #31 NOT #47 | 108 |

*Cochrane Library*

| **#** | **Terms** | **Number of hits November 2020** |
| --- | --- | --- |
|  | **Population** |  |
| #1 | MeSH descriptor: [Inflammatory Bowel Diseases] explode all trees | 3,325 |
| #2 | MeSH descriptor: [Colitis, Ulcerative] explode all trees | 1,585 |
| #3 | MeSH descriptor: [Proctitis] explode all trees | 139 |
| #4 | (ulcer* near/2 colitis):ti,ab,kw | 4,846 |
| #5 | (inflamm* near/2 (colon* or bowel)):ti,ab,kw | 3,245 |
| #6 | (pancolitis or rectitis or proctocolitis or procto-colitis or colorectitis or rectocolitis or rectocolitis or recto-sigmoiditis or rectosigmoiditis or procto-sigmoiditis or proctosigmoiditis or proctitis):ti,ab,kw | 623 |
| #7 | ((total or sub-total or subtotal or extensive or left-sided or universal) near colitis):ti,ab,kw | 237 |
| #8 | #1 OR #2 OR #3 OR #4 OR #5 OR #6 OR #7 | 8,348 |
|  | **Intervention** |  |
| #9 | Ustekinumab:ti,ab,kw OR Stelara:ti,ab,kw | 798 |
|  | **Combine** |  |
| #10 | #8 AND #9 | 138 |
|  | **Randomised control trials (RCT) search terms** |  |
| #11 | Controlled clinical trial:pt | 471,313 |
| #12 | Randomized controlled trial:pt | 499,031 |
| #13 | Comparative study:pt | 167,902 |
| #14 | Clinical study:pt | 193,888 |
| #15 | Clinical trial:pt | 471,313 |
| #16 | Multicenter study:pt | 88,811 |
| #17 | (prospective OR randomised OR randomized OR trial OR phase):ti,ab,kw | 1,088,129 |
| #18 | #11 OR #12 OR #13 OR #14 OR #15 OR #16 OR #17 | 1,268,099 |
|  | **Exclusion terms** |  |
| #19 | Observational study:pt | 2,708 |
| #20 | Review:pt | 17,879 |
| #21 | Editorial:pt | 2,745 |
| #22 | Letter:pt | 12,006 |
| #23 | Comment:pt | 1,879 |
| #24 | Guideline:pt | 31 |
| #25 | (observational OR longitudinal OR retrospective):ti,ab,kw | 61,644 |
| #26 | (“Case not*” OR “Case repor*”):ti,ab,kw | 51 |
| #27 | (biomarker OR in-vitro OR epigenetic):ti,ab,kw | 32,708 |
| #28 | (pharmacokinetics OR pharmacokinetic)ti,ab,kw | 88 |
| #29 | #19 OR #20 OR #21 OR #22 OR #23 OR #24 OR #25 OR #26 OR #27 OR #28 | 124,457 |
|  | **Combine** |  |
| #30 | #10 AND #18 | 125 |
|  | **Limits** |  |
| #31 | #30 NOT #29 (and limit to trials) | 107 |

**Supplementary table 3.** University of York Centre for Reviews and Dissemination search terms.

| **#** | **Search terms** | **Number of hits May 2019** | **Number of hits May 2019- November 2020** |
| --- | --- | --- | --- |
| 1 | MeSH DESCRIPTOR Colitis, Ulcerative EXPLODE ALL TREES | 153 | 153 |
| 2 | ((ulcerative colitis)) and ((Systematic review:ZDT and Bibliographic:ZPS) OR (Systematic review:ZDT and Abstract:ZPS) OR (Cochrane review:ZDT) OR (Cochrane related review record:ZDT) OR (Economic evaluation:ZDT and Bibliographic:ZPS) OR (Economic evaluation:ZDT and Abstract:ZPS) OR Project record:ZDT OR Full publication record:ZDT) IN DARE, NHSEED, HTA | 229 | 229 |
| 3 | #1 or #2 | 261 | 261 |
| 4 | #3 since July 2019 | - | 0 |

**Supplementary table 4.** Eligibility criteria for systematic searching.

| **Variable** | **Inclusion criteria** | **Exclusion criteria** |
| --- | --- | --- |
| Population | - Adult (≥18 years of age) patients with moderately to severely active ulcerative colitis in adults when conventional therapy cannot be tolerated or the disease has responded inadequately or lost response to conventional^§^ (biologic-naïve) or biologic (biologic experienced/failed) treatment*. | - Juvenile or paediatric ulcerative colitis - Presence of Crohn's disease, indeterminate colitis, ischemic colitis, fulminant colitis, ulcerative proctitis, or toxic mega-colon - Patients with mild UC; if the study population is mixed (i.e., mild to severe), exclude those studies in which data are not reported separately for moderate or severely active UC - Patients without UC |
| Interventions | - Biologic drugs, including:   - - Adalimumab (Humira, Trudexa, ABP 501, BI695501, CHS-1420, GP2017, M923, PF-06410293)     - Apremilast (Otezla)     - Golimumab (Simponi)     - Infliximab (Remicade)     - Ustekinumab (Stelara)     - Vedolizumab (Entyvio) - Biosimilars, including:   - - Adalimumab biosimilars (Amjevita/ABP-501; Cyltezo/BI 695501; SB5)     - Infliximab biosimilars (Remsima; Inflectra; Flixabi; Renflexis/SB2, CT-P13; PF-06438179; PF-06438179; ABP501) - Targeted synthetic drugs including:   - - Baricitinib (Oluminant)     - Tofacitinib (Xeljanz)     - Filgotinib (GLPG0634, GS-6034)     - Peficitinib (ASP015K)     - Upadaacitinib (ABT-494)     - PF-06651600     - PF-06700841     - TD-1473 - Surgical procedures for managing moderate to severe ulcerative colitis | - Studies that do not have an intervention of interest in more than 1 arm - Non-pharmacological studies, e.g., exercise, Chinese medicine, etc. - Studies only comparing conventional therapies including aminosalicylates and corticosteroids |
| Comparators | - Any comparison between any of the listed interventions and each other or placebo | - Studies not reporting on at least one of the interventions of interest |
| Outcomes | - †To be included in the review, a study must report at least 1 of the following outcomes of interest: - †Efficacy measurements:   - - Mayo Clinic Score (MCS).     - Partial Mayo Score     - Ulcerative colitis symptom score.     - Clinical response     - Histologic remission     - Clinical Remission     - Corticosteroid-free remission     - Endoscopic/Mucosal healing - Surgery - †Safety outcomes reported at study endpoint:   - - Overall rate of AEs     - Overall rate of serious AEs     - Discontinuations due to adverse events     - Lack of efficacy     - AEs - Individual AEs, such as the following:   - - Arthralgia     - Infections, including herpes zoster     - Nasopharyngitis     - Intestinal perforation     - Death     - Initial or prolonged inpatient hospitalisation | - Outcomes of interest not reported |
| Study design | - Randomised, controlled, prospective clinical trials (above phase 1)   - - RCTs in which patients are re-randomised at the end of induction     - RCTs in which patients are stay in their randomised groups at the end of induction (treat-through) - Long-term follow-up studies (e.g. open-label follow-up studies with continuation of treatments in their respective randomised group) - Post hoc analyses of patient sub-groups of interest (biologic-naïve/biologic experienced/biologic failed) | - Phase 1 studies - Non-randomised clinical trials - Single-arm studies - Long-term follow-up or extension studies of RCTs of over 1 year (post maintenance phase data) - Maintenance studies and step-down treatment studies - Preclinical studies - Prognostic studies - Retrospective observational studies - Prospective observational studies - Case report - Case series - Animal models |
| Publication type | - Peer-reviewed publications - Clinical trial records - Conference proceedings | - Commentaries and letters (publication type) - Pooled analyses - Non-systematic reviews - Systematic reviews (including meta-analyses)† - Consensus reports |
| Language restrictions | - English language only | - Studies published in languages other than English |
| Date restriction | - None | - NA |
| AE: adverse event; UC: ulcerative colitis; VAS: visual analogue scale.  *Disease severity is defined according to the Truelove and Witts’ severity index in line with NICE clinical guidance.[[1](#_ENREF_1)] Moderate to severely active ulcerative colitis: total Mayo score of 6 to 12.  §Conventional therapy considered to include topical or oral aminosalicylate, corticosteroids, mercaptopurine, azathioprine or prednisolone  †Systematic reviews and meta-analyses will be used for identification of primary studies that may have been missed in the electronic searches | | |

**Supplementary searching**

Additional supplementary searching included relevant congresses held between 2016 and 2020 (American College of Gastroenterology, Crohn’s and Colitis UK, the European Crohn’s and Colitis Organisation, the British Society of Gastroenterology and the International Society for Pharmacoeconomics and Outcomes research), health technology and regulatory databases via the University of York Centre for Reviews and Dissemination (Supplementary file 1), and the reference lists of identified systematic reviews and meta-analyses.

## Trials excluded from the network meta-analysis

**Supplementary table 5.** Trials identified that were excluded from the network meta-analysis with reasons.

| **Trial Name/Author, year** | **Reason for exclusion from NMA** |
| --- | --- |
| Armuzzi et al. (2004) [[2](#_ENREF_2)] | No outcome data for NMA |
| Bruce et al. (2014) [[3](#_ENREF_3)] | No outcome data for NMA |
| Colombel et al. (2013) [[4](#_ENREF_4)] | No outcome data for NMA |
| Colombel et al. (2020) [[5](#_ENREF_5)] | Mixed bio-naïve/experienced population |
| D’Haens et al. (2016) [[6](#_ENREF_6)] | Outcomes reported in primary study |
| Danese et al. (2018) [[7](#_ENREF_7)] | Intervention not relevant |
| Danese et al. (2020) [[8](#_ENREF_8)] | Mixed bio-naïve/experienced population |
| Euctr et al. (2009) [[9](#_ENREF_9)] | Mixed bio-naïve/experienced population |
| Euctr et al. (2014) [[10](#_ENREF_10)] | Intervention not of interest |
| Feagan et al. (2013) [[11](#_ENREF_11)] | Outcomes reported in primary study |
| Feagan et al. (2014) [[12](#_ENREF_12)] | No outcome data for NMA |
| Feagan et al. (2018) [[13](#_ENREF_13)] | No outcome data for NMA |
| Gibson et al. (2016) [[14](#_ENREF_14)] | No outcome data for NMA |
| Hibi et al. (2017) [[15](#_ENREF_15)] | Lack of data (small trial population), single arm induction period |
| Kaser et al. (2017) [[16](#_ENREF_16)] | No outcome data for NMA |
| Leiper et al. (2011) [[17](#_ENREF_17)] | Intervention not relevant |
| Lichtenstein et al. (2018) [[18](#_ENREF_18)] | No outcome data for NMA |
| Panaccione et al. (2014) [[19](#_ENREF_19)] | Comparator arm could not be linked to NMA |
| Panes et al. (2019) [[20](#_ENREF_20)] | Mixed bio-naïve/experienced population |
| Parikh et al. (2012) [[21](#_ENREF_21)] | Mixed bio-naïve/experienced population |
| Parikh et al. (2012) [[22](#_ENREF_22)] | Outcomes reported in primary study |
| Parikh et al. (2013) [[23](#_ENREF_23)] | No outcome data for NMA |
| Probert et al. (2003) [[24](#_ENREF_24)] | Inappropriate measure of remission |
| Reinish et al. (2012) [[25](#_ENREF_25)] | No outcome data for NMA |
| Reinish et al. (2013) [[26](#_ENREF_26)] | Outcomes reported in primary study |
| Reinish et al. (2018) [[27](#_ENREF_27)] | No outcome data for NMA |
| Rutgeerts et al. (2015) [[28](#_ENREF_28)] | Non-EMA approved dose |
| Sandborn et al. (2012) [[29](#_ENREF_29)] | Phase II trial |
| Sandborn et al. (2013) [[30](#_ENREF_30)] | Outcomes reported in primary study |
| Sandborn et al. (2018) [[31](#_ENREF_31)] | Mixed bio-naïve/experienced population |
| Sandborn et al. (2020) [[32](#_ENREF_32)] | Intervention not relevant |
| Sands et al. (2018) [[33](#_ENREF_33)] | Intervention not of interest |
| Sands et al. (2018) [[34](#_ENREF_34)] | Mixed bio-naïve/experienced population |
| Schreiber et al. (2019) [NCT 2015] [[35](#_ENREF_35)] | Mixed bio-naïve/experienced population |

## Cochrane risk of bias assessment

**Supplementary figure 1.** Risk of bias of studies included in the NMA


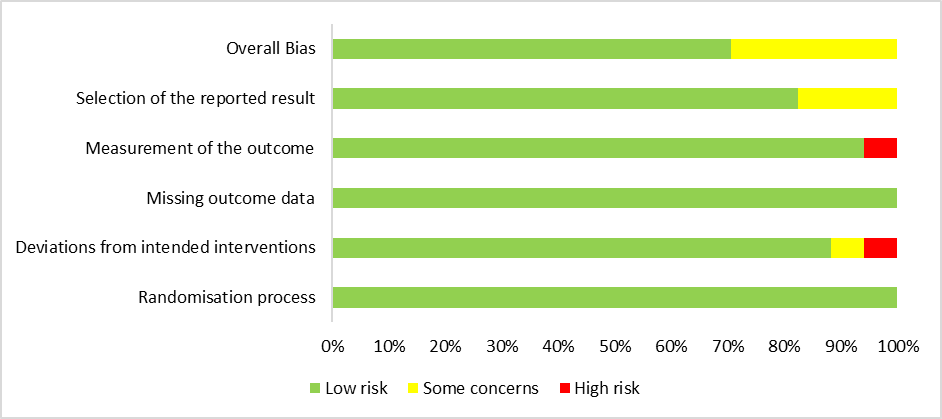


## Summary of potential biases in the analysis

**Supplementary table 6.** Summary of potential biases in the biologic-naïve analysis, their direction, magnitude and any action taken to adjust or control for bias

| Comparison | | Trials | Potential source, direction, magnitude of bias and adjustments/controls | | | | | | | | | | | | | | | | | | | | | | |
| --- | --- | --- | --- | --- | --- | --- | --- | --- | --- | --- | --- | --- | --- | --- | --- | --- | --- | --- | --- | --- | --- | --- | --- | --- | --- |
|  |  | | **Trial design** | | | | **Outcome definitions** | | | | **Population definition** | | | | | | **Patient characteristics** | | | | | **Assessment timepoints** | | | |
|  |  | | **Bias possible*** | **Direction of bias** | **Magnitude of bias** | **Adjustment/control** | **Bias possible** | **Direction of bias** | **Magnitude of bias** | **Adjustment/control** | | **Bias possible**** | **Direction of bias** | **Magnitude of bias** | **Adjustment/control** | **Bias possible***** | | **Direction of bias** | **Magnitude of bias** | **Adjustment/control** | **Bias possible****** | | **Direction of bias** | **Magnitude of bias** | **Adjustment/control** |
| ADA vs placebo | ULTRA 1[[36](#_ENREF_36)] | |  |  |  |  |  |  |  |  | |  |  |  |  | **🗸** | | Cannot determine | Uncertain | None |  | |  |  |  |
|  | ULTRA 2[[37](#_ENREF_37),[38](#_ENREF_38)] | |  |  |  |  |  |  |  |  | |  |  |  |  | **🗸** | | Cannot determine | Uncertain | None |  | |  |  |  |
|  | VARSITY[[39](#_ENREF_39)] | |  |  |  |  |  |  |  |  | |  |  |  |  | **🗸** | | Cannot determine | Uncertain | None | **🗸** | | Possibly favours intervention | Uncertain | None |
| FIL vs placebo | SELECTION | | **🗸** | Favours intervention | Large | Re-weighting |  |  |  |  | |  |  |  |  | **🗸** | | Cannot determine | Uncertain | None | **🗸** | | Possibly favours intervention | Uncertain | None |
| GOL vs placebo | PURSUIT-SC Induction[[40](#_ENREF_40)], PURSUIT-SC Maintenance[[41](#_ENREF_41)] | | **🗸** | Favours intervention | Large | Re-weighting |  |  |  |  | |  |  |  |  | **🗸** | | Cannot determine | Uncertain | None |  | |  |  |  |
| IFX vs placebo | ACT 1[[42](#_ENREF_42)] | |  |  |  |  |  |  |  |  | |  |  |  |  | **🗸** | | Cannot determine | Uncertain | None |  | |  |  |  |
|  | ACT 2[[42](#_ENREF_42)] | |  |  |  |  |  |  |  |  | |  |  |  |  | **🗸** | | Cannot determine | Uncertain | None |  | |  |  |  |
|  | Kobayashi 2016 (Japic)[[43](#_ENREF_43)] | |  |  |  |  |  |  |  |  | |  |  |  |  | **🗸** | | Cannot determine | Uncertain | None |  | |  |  |  |
|  | Jiang 2015[[44](#_ENREF_44)] | |  |  |  |  |  |  |  |  | |  |  |  |  | **🗸** | | Cannot determine | Uncertain | None |  | |  |  |  |
|  | NCT01551290[[45](#_ENREF_45)] | |  |  |  |  |  |  |  |  | |  |  |  |  | **🗸** | | Cannot determine | Uncertain | None |  | |  |  |  |
| TOFA vs placebo | OCTAVE 1[[46](#_ENREF_46)], OCTAVE 2[[46](#_ENREF_46)], OCTAVE SUSTAIN[[47](#_ENREF_47)] | |  |  |  |  | **🗸** | Favours comparators | Small | Not possible | |  |  |  |  | **🗸** | | Cannot determine | Uncertain | None | **🗸** | | Possibly favours intervention | Uncertain | None |
| UST vs. placebo | UNIFI[[48](#_ENREF_48)] | | **🗸** | Favours intervention | Large | Re-weighting |  |  |  |  | | **🗸** | Possibly favours comparator | Small | Not possible | **🗸** | | Cannot determine | Uncertain | None |  | |  |  | None |
| VDZ vs placebo | GEMINI 1[[49](#_ENREF_49)] | | **🗸** | Favours intervention | Large | Re-weighting |  |  |  |  | |  |  |  |  | **🗸** | | Cannot determine | Uncertain | None | **🗸** | | Possibly favours comparator | Uncertain | None |
|  | VISIBLE[[50](#_ENREF_50)] | | **🗸** | Favours intervention | Large | Re-weighting |  |  |  |  | |  |  |  |  | **🗸** | | Cannot determine | Uncertain | None | **🗸** | | Possibly favours comparator | Uncertain | None |
| VDZ vs ADA | VARSITY[[39](#_ENREF_39)] | |  |  |  |  |  |  |  |  | |  |  |  |  | **🗸** | | Cannot determine | Uncertain | None | **🗸** | | Possibly favours intervention | Uncertain | None |
| * Treat through is considered the base case here compared with which, re-randomising patients introduces bias by actively selecting for treatment responders in maintenance.  ** No biologic exposure is considered the base case here as the more standard definition across trials, whereas no biologic failure definition may introduce some differences in patients.  *** Particularly baseline disease severity and concomitant medications  **** 8 weeks induction and 52 weeks maintenance assessment timepoints were considered the base case as most commonly used. Trials deviating from these timepoints may have introduced some bias. | | | | | | | | | | | | | | | | | | | | | | | | | |

**Supplementary table 7.** Summary of potential biases in the biologic-experienced analysis, their direction, magnitude and any action taken to adjust or control for bias

| Comparison | | Trials | Potential source, direction, magnitude of bias and adjustments/controls | | | | | | | | | | | | | | | | | | | | | |
| --- | --- | --- | --- | --- | --- | --- | --- | --- | --- | --- | --- | --- | --- | --- | --- | --- | --- | --- | --- | --- | --- | --- | --- | --- |
|  |  | | **Trial design** | | | | **Outcome definitions** | | | | **Population definition** | | | | **Patient characteristics** | | | | **Assessment timepoints** | | | | |  |
|  |  | | **Bias possible*** | **Direction of bias** | **Magnitude of bias** | **Adjustment/control** | **Bias possible** | **Direction of bias** | **Magnitude of bias**** | **Adjustment/control** | **Bias possible** | **Direction of bias** | **Magnitude of bias** | **Adjustment/control** | **Bias possible***** | **Direction of bias** | **Magnitude of bias** | **Adjustment/control** | **Bias possible****** | **Direction of bias** | **Magnitude of bias** | **Adjustment/control** | |  |
| ADA vs placebo | ULTRA 1[[36](#_ENREF_36)] | |  |  |  |  |  |  |  |  |  |  |  |  | **🗸** | Cannot determine | Uncertain | None |  |  |  |  | |  |
|  | ULTRA 2[[37](#_ENREF_37),[38](#_ENREF_38)] | |  |  |  |  |  |  |  |  | **🗸** | Possibly favours intervention | Small | Not possible | **🗸** | Cannot determine | Uncertain | None |  |  |  |  | |  |
|  | VARSITY[[39](#_ENREF_39)] | |  |  |  |  |  |  |  |  | **🗸** | Possibly favours intervention | Small | Not possible | **🗸** | Cannot determine | Uncertain | None | **🗸** | Possibly favours intervention | Uncertain | None | |  |
| FIL vs placebo | SELECTION | | **🗸** | Favours intervention | Large | Re-weighting |  |  |  |  |  |  |  |  | **🗸** | Cannot determine | Uncertain | None | **🗸** | Possibly favours intervention | Uncertain | None | |  |
| GOL vs placebo | PURSUIT-SC Induction[[40](#_ENREF_40)] PURSUIT-SC Maintenance[[41](#_ENREF_41)] | | **🗸** | Favours intervention | Large | Re-weighting |  |  |  |  |  |  |  |  | **🗸** | Cannot determine | Uncertain | None |  |  |  |  | |  |
| IFX vs placebo | ACT 1[[42](#_ENREF_42)] | |  |  |  |  |  |  |  |  |  |  |  |  | **🗸** | Cannot determine | Uncertain | None |  |  |  |  | |  |
|  | ACT 2[[42](#_ENREF_42)] | |  |  |  |  |  |  |  |  |  |  |  |  | **🗸** | Cannot determine | Uncertain | None |  |  |  |  | |  |
|  | Kobayashi 2016 (Japic)[[43](#_ENREF_43)] | |  |  |  |  |  |  |  |  |  |  |  |  | **🗸** | Cannot determine | Uncertain | None |  |  |  |  | |  |
|  | Jiang 2015[[44](#_ENREF_44)] | |  |  |  |  |  |  |  |  |  |  |  |  | **🗸** | Cannot determine | Uncertain | None |  |  |  |  | |  |
|  | NCT01551290[[45](#_ENREF_45)] | |  |  |  |  |  |  |  |  |  |  |  |  | **🗸** | Cannot determine | Uncertain | None |  |  |  |  | |  |
| TOFA vs placebo | OCTAVE 1[[46](#_ENREF_46)] OCTAVE 2[[46](#_ENREF_46)] OCTAVE SUSTAIN[[47](#_ENREF_47)] | |  |  |  |  | **🗸** | Favours comparators | Small | Not possible |  |  |  |  | **🗸** | Cannot determine | Uncertain | None | **🗸** | Possibly favours intervention | Uncertain | None | |  |
| UST vs. placebo | UNIFI[[48](#_ENREF_48)] | | **🗸** | Favours intervention | Large | Re-weighting |  |  |  |  |  |  |  |  | **🗸** | Cannot determine | Uncertain | None |  |  |  | None | |  |
| VDZ vs placebo | GEMINI 1[[49](#_ENREF_49)] | | **🗸** | Favours intervention | Large | Re-weighting |  |  |  |  |  |  |  |  | **🗸** | Cannot determine | Uncertain | None | **🗸** | Possibly favours comparator | Uncertain | None | |  |
|  | VISIBLE[[50](#_ENREF_50)] | | **🗸** | Favours intervention | Large | Re-weighting |  |  |  |  |  |  |  |  | **🗸** | Cannot determine | Uncertain | None | **🗸** | Possibly favours comparator | Uncertain | None | |  |
| VDZ vs ADA | VARSITY[[39](#_ENREF_39)] | |  |  |  |  |  |  |  |  | **🗸** | Possibly favours intervention | Small | Not possible | **🗸** | Cannot determine | Uncertain | None | **🗸** | Possibly favours intervention | Uncertain | None | |  |
| * Treat through is considered the base case here compared with which, re-randomising patients introduces bias by actively selecting for treatment responders in maintenance.  ** Biologic failure is considered the base case here as the more standard definition across trials, whereas biologic exposed definition may introduce some differences in patients.  *** Particularly baseline disease severity and concomitant medications  **** 8 weeks induction and 52 weeks maintenance assessment timepoints were considered the base case as most commonly used. Trials deviating from these timepoints may have introduced some bias. | | | | | | | | | | | | | | | | | | | | | | |  |  |

## Re-weighting: methodology

Re-weighting of treat through trials, an accepted approach by NICE, was performed where analysis of maintenance phase response and remission outcomes were restricted to induction phase responders. The induction phase number of responders was assumed as the denominator (a proxy for those entering the maintenance phase). Number of patients achieving sustained clinical response (response at both induction and maintenance phase) was assumed as the numerator. A similar process was used for clinical remission; where number of patients achieving clinical remission was the numerator used, unless the number of patients achieving remission *given induction response* was reported separately for the given study (this was the case for ULTRA 2). This approach assumes that all maintenance phase remission patients were at least responders in the induction phase.

## Trials included in the induction phase analyses

**Supplementary table 8** Trials included in the induction analyses: study characteristics and efficacy outcomes

|  |  | |  |  | Biologic-naïve^a^ | | | | Biologic-experienced^a^ | | | |
| --- | --- | --- | --- | --- | --- | --- | --- | --- | --- | --- | --- | --- |
| Trial | Drug development phase | Treatment duration (induction phase),  weeks | | Treatment arms | N | Response without remission,  n (%) | Response with remission, n (%) | Endoscopic mucosal healing,  n (%) | N | Response without remission, n (%) | Response with remission, n (%) | Endoscopic mucosal healing, n (%) |
| ACT 1 [[51](#_ENREF_51)] | 3 | 8 | | Placebo | 121 | 27 (22.3) | 18 (14.9) | 41 (33.9) | - | - | - | - |
|  |  |  | | Infliximab  5 mg/kg | 121 | 37 (30.6) | 47 (38.8) | 75 (62) | - | - | - | - |
| ACT 2 [[51](#_ENREF_51)] | 3 | 8 | | Placebo | 123 | 29 (23.6) | 7 (5.7) | 38 (30.9) | - | - | - | - |
|  |  |  | | Infliximab  5 mg/kg | 121 | 37 (30.6) | 41 (33.9) | 73 (60.3) | - | - | - | - |
| GEMINI 1 [[52](#_ENREF_52)] | 3 | 6 | | Placebo | 76 | 15 (19.7) | 5 (6.6) | 19 (25.0) | 63 | 11 (17.5) | 2 (3.2) | 13 (20.6) |
|  |  |  | | Vedolizumab  300 mg | 130 | 39 (30.0) | 30 (23.1) | 64 (49.2) | 82 | 24 (29.3) | 8 (9.8) | 25 (30.5) |
| Jiang 2015 [[53](#_ENREF_53)] | 3 | 8 | | Placebo | 41 | 6 (14.6) | 9 (22.0) | 10 (24.4) | - | - | - | - |
|  |  |  | | Infliximab  5 mg/kg | 41 | 10 (24.4) | 22 (53.7) | 24 (58.5) | - | - | - | - |
| Kobayashi 2016 [[54](#_ENREF_54)] | 3 | 8 | | Placebo | 104 | 26 (25.0) | 11 (10.6) | 29 (27.9) | - | - | - | - |
|  |  |  | | Infliximab  5 mg/kg | 104 | 36 (34.6) | 21 (20.2) | 48 (46.2) | - | - | - | - |
| NCT01551290 [[55](#_ENREF_55)] | 3 | 8 | | Placebo | 49 | 11 (22.4) | 5 (10.2) | 8 (16.3) | - | - | - | - |
|  |  |  | | Infliximab  5 mg/kg | 50 | 21 (42.0) | 11 (22.0) | 17 (34.0) | - | - | - | - |
| OCTAVE 1 [[56](#_ENREF_56)] | 3 | 8 | | Placebo | 57 | 13 (22.8) | 9 (15.8) | 15 (26.3) | 64 | 14 (21.9) | 1 (1.6) | 4 (6.3) |
|  |  |  | | Tofacitinib  10 mg | 222 | 87 (39.2) | 56 (25.2) | 88 (39.6) | 243 | 97 (39.9) | 27 (11.1) | 58 (23.9) |
| OCTAVE 2 [[56](#_ENREF_56)] | 3 | 8 | | Placebo | 47 | 14 (29.8) | 4 (8.5) | 9 (19.1) | 60 | 14 (23.3) | 0 (0) | 4 (6.7) |
|  |  |  | | Tofacitinib  10 mg | 195 | 83 (42.6) | 43 (22.1) | 71 (36.4) | 222 | 87 (39.2) | 26 (11.7) | 48 (21.6) |
| PURSUIT-SC Induction Phase 2/3 [[57](#_ENREF_57)] | 2/3 | 6 | | Placebo | 28 | 10 (35.7) | 3 (10.7) | 12 (42.9) | - | - | - | - |
|  |  |  | | Golimumab 200/100 mg | 30 | 9 (30.0) | 6 (20) | 15 (50.0) | - | - | - | - |
| PURSUIT-SC Induction Phase 3 [[57](#_ENREF_57)] | 3 | 6 | | Placebo | 258 | 62 (24.0) | 16 (6.2) | 72/251 (28.7) | - | - | - | - |
|  |  |  | | Golimumab 200/100 mg | 258 | 86 (33.3) | 45 (17.4) | 107/253 (42.3) | - | - | - | - |
| SELECTION [[58](#_ENREF_58)] | 2/3 | 10 | | Placebo | 137 | 47 (34.3) | 17 (12.4) | 28 (20.4) | 142 | 19 (13.4) | 6 (4.2) | 11 (7.7) |
|  |  |  | | Filgotinib 100 mg | 277 | 117 (42.2) | 47 (17.0) | 73 (26.4) | 285 | 85 (29.8) | 17 (6.0) | 37 (13.0) |
|  |  |  | | Filgotinib 200 mg | 245 | 103 (42.0) | 60 (24.5) | 83 (33.9) | 262 | 114 (43.5) | 25 (9.5) | 45 (17.2) |
| ULTRA 1 [[59](#_ENREF_59)] | 3 | 8 | | Placebo | 130 | 46 (35.4) | 12 (9.2) | 54 (41.5) | - | - | - | - |
|  |  |  | | Adalimumab 160/80/40 mg | 130 | 47 (36.2) | 24 (18.5) | 61 (46.9) | - | - | - | - |
| ULTRA 2 [[37](#_ENREF_37),[60](#_ENREF_60)] | 3 | 8 | | Placebo | 145 | 40 (27.6) | 16 (11.0) | 51 (35.2) | 101 | 22 (21.8) | 7 (6.9) | 27 (26.7) |
|  |  |  | | Adalimumab 160/80/40 mg | 150 | 57 (38.0) | 32 (21.3) | 74 (49.3) | 98 | 27 (27.6) | 9 (9.2) | 28 (28.6) |
| UNIFI [[61](#_ENREF_61)] | 3 | 8 | | Placebo | 158 | 41 (25.9) | 15 (9.5) | 33 (20.9) | 161 | 42 (26.1) | 2 (1.2) | 11 (6.8) |
|  |  |  | | Ustekinumab 90 mg | 156 | 75 (48.1) | 29 (18.6) | 52 (33.3) | 166 | 74 (44.6) | 21 (12.7) | 35 (21.1) |
| VARSITY [[61](#_ENREF_61)] | 3 | 14 | | Adalimumab | 305 | 79 (25.9) | 72 (23.6) | - | 81 | 16 (19.8) | 10 (12.3) | - |
|  |  |  | | Vedolizumab 300 mg | 304 | 129 (42.4) | 84 (27.6%) | - | 79 | 26 (32.9) | 18 (22.8) | - |

^a^ For each component of each efficacy outcome, the numerator and denominator meeting the endpoint were extracted for analysis

Trials included in the maintenance phase analyses

**Supplementary table 9** Trials included in the maintenance phase analyses: study characteristics and efficacy outcomes

|  |  |  |  | Biologic-naïve^a^ | | | | Biologic-experienced^a^ | | | |
| --- | --- | --- | --- | --- | --- | --- | --- | --- | --- | --- | --- |
| Trial | **Drug development phase** | **Treatment duration (maintenance phase), weeks** | **Treatment arms** | **N** | **Response without remission, n (%)** | **Response with remission, n (%)** | **Endoscopic mucosal healing, n (%)** | **N** | **Response without remission, n (%)** | **Response with remission, n (%)** | **Endoscopic mucosal healing, n (%)** |
| ACT 1 [[51](#_ENREF_51)] | 3 | 54 | Placebo | 121 | 7 (15.6) | 10 (22.2) | 22 (18.2) | - | - | - | - |
|  |  |  | Infliximab 5 mg/kg Q8W | 121 | 5 (6.0) | 42 (50.0) | 55 (45.5) | - | - | - | - |
| GEMINI 1 [[52](#_ENREF_52)] | 3 | 46 | Placebo | 79 | 6 (7.6) | 15 (19.0) | 19 (24.1) | 38 | 4 (10.5) | 2 (5.3) | 3 (7.9) |
|  |  |  | Vedolizumab 300 mg Q8W | 72 | 14 (19.4) | 33 (45.8) | 43 (59.7) | 40 | 1 (2.5) | 14 (35.0) | 19 (47.5) |
|  |  |  | Vedolizumab 300 mg Q4W | 73 | 6 (8.2) | 35 (47.9) | 44 (60.3) | 43 | 6 (14.0) | 16 (37.2) | 18 (41.9) |
| OCTAVE SUSTAIN [[62](#_ENREF_62)] | 3 | 52 | Placebo | 109 | 15 (13.8) | 12 (11.0) | - | 89 | 3 (3.4) | 10 (11.2) | - |
|  |  |  | Tofacitinib 5 mg BID | 115 | 17 (14.8) | 48 (41.7) | - | 83 | 17 (20.5) | 20 (24.1) | - |
|  |  |  | Tofacitinib 10 mg BID | 104 | 21 (20.2) | 46 (44.2) | - | 93 | 21 (22.6) | 34 (36.6) | - |
| PURSUIT-SC Maintenance [[57](#_ENREF_57)] | 2/3 | 54 | Placebo | 154 | 14 (9.1) | 34 (22.1) | - | - | - | - | - |
|  |  |  | Golimumab 100 mg Q4W | 151 | 21 (13.9) | 51 (33.8) | - | - | - | - | - |
|  |  |  | Golimumab 50 mg Q4W | 151 | 24 (15.9) | 50 (33.1) | - | - | - | - | - |
| SELECTION | 2/3 | 48 | Placebo | 54 | 15 (27.8) | 7 (13.0) | 10 (18.5) | 44 | 8 (18.2) | 2 (4.5) | 5 (11.4) |
|  |  |  | Filgotinib 200 mg QD | 107 | 31 (29.0) | 49 (45.8) | 57 (53.3) | 92 | 33 (35.9) | 20 (21.7) | 24 (26.1) |
|  |  |  | Placebo | 54 | 19 (35.2) | 9 (16.7) | 13 (24.1) | 35 | 4 (11.4) | 3 (8.6) | 4 (11.4) |
|  |  |  | Filgotinib 100 mg QD | 105 | 35 (33.3) | 26 (24.8) | 32 (30.5) | 67 | 13 (19.4) | 13 (19.4) | 14 (20.9) |
| ULTRA 2 [[37](#_ENREF_37),[60](#_ENREF_60)] | 3 | 44 | Placebo | 145 | 10 (17.9) | 14 (25.0) | 28 (19.3) | 29 | 3 (10.3) | 3 (10.3) | 10 (9.9) |
|  |  |  | Adalimumab 160/80/40 mg Q2W | 150 | 16 (18.0) | 28 (31.5) | 47 (31.3) | 36 | 7 (19.4) | 8 (22.2) | 15 (15.3) |
| UNIFI [[63](#_ENREF_63)] | 3 | 44 | Placebo | 87 | 17 (19.5) | 27 (31.0) | 30 (34.5) | 88 | 19 (21.6) | 15 (17.0) | 20 (22.7) |
|  |  |  | Ustekinumab 90 mg Q12W | 102 | 28 (27.5) | 50 (49.0) | 57 (55.9) | 70 | 23 (32.9) | 16 (22.9) | 18 (25.7) |
|  |  |  | Ustekinumab 90 mg Q8W | 85 | 25 (29.4) | 41 (48.2) | 49 (57.6) | 91 | 23 (25.3) | 36 (39.6) | 41 (45.1) |
| VARSITY [[61](#_ENREF_61)] | 3 | 52 | Adalimumab 160/80/40 mg Q2W | 305 | 24 (15.8) | 74 (49.0) | 90 (29.5) | 44 | 8 (18) | 16 (36.0) | 17 (21.0) |
|  |  |  | Vedolizumab 300 mg Q8W | 304 | 55 (25.9) | 104 (49.1) | 131 (43.1) | 26 | 0 (0) | 13 (50.0) | 21 (26.6) |
| VISIBLE 1 [[64](#_ENREF_64)] | 3 | 46 | Placebo | 37 | - | 7 (18.9) | - | 19 | - | 1 (5.3) | - |
|  |  |  | Vedolizumab 108 mg Q2W SC | 67 | - | 36 (53.7) | - | 29 | - | 13 (33.3) | - |
|  |  |  | Vedolizumab 300 mg Q8W | 32 | - | 17 (53.1) | - | 22 | - | 6 (27.3) | - |

^a^ For each component of each efficacy outcome, the numerator and denominator meeting the endpoint were extracted for analysis.

BID, twice daily; SC, subcutaneous; QD, once daily; QXW, every X weeks

## Re-weighting: results

**Supplementary table 9.** Treat-through maintenance phase re-weighting.

| **Trial** | **Treatment** | **Raw data** | | | | **Imputation data (maintenance phase)** | | | | | |
| --- | --- | --- | --- | --- | --- | --- | --- | --- | --- | --- | --- |
|  |  | **Maintenance phase (n)** | **Induction phase responders, n (%)** | **Sustained responders at maintenance, n (%)** | **(1) Assumed patients re-randomised (n)** | | **(2) Assumed maintenance clinical responders, n (%)** | **(3) Assumed maintenance clinical remission** | **No response^a^**  **n (%) (1-2)** | **Response without remission^a^**  **n (%) (2-3)** | **Response with remission^a^**  **n (%) (3)** |
| **Biologic-naïve** | | | | | | | | | | | |
| **ULTRA 2** | ADA 160/80/40mg Q2W | 150 | 89 (59.3) | 44 (29.3) | 89 | | 44/89 (**49.4)** | 28**^a^**/89 (31.5) | 45 (50.6) | 16 (17.9) | 28 (31.5) |
|  | PBO | 145 | 56 (38.6) | 24 (16.6) | 56 | | 24/56 (**42.9)** | 14/56 (25.0)^d^ | 32 (57.1) | 10 (17.9) | 14 (25.0) |
| **ACT 1** | IFX 5mg/kg Q8W | 121 | 84 (69.4) | 47 (38.8) | 84 | | 47/84 (**56.0**) | 42/84 (**50.0**) | 37 (44.0) | 5 (6.0) | 42 (50.0) |
|  | PBO | 121 | 45 (37.2) | 17 (14.0) | 45 | | 17/45 (**37.8**) | 10/45 (**22.0**)^e^ | 28 (23.1) | 7 (5.8) | 10 (8.3)^f^ |
| **VARSITY^b^** | VDZ 300mg Q8W | 304 | 212 (69.7) | 159 (52.3) | 212 | | 159 (75)^c^ | 104 (49.1)^f^ | 53 (25.0) | 55 (25.9) | 104 (49.1) |
|  | ADA 160/80/40mg Q2W | 305 | 151 (49.5) | 98 (32.1) | 152 | | 98 (64.9)^c^ | 74 (49.0)^f^ | 53 (35.1) | 24 (15.9) | 74 (49.0) |
| **Biologic-experienced** | | | | | | | | | | | |
| **ULTRA 2** | ADA 160/80/40mg Q2W | 98 | 36 (36.7) | 15 (15.3) | 36 | | 15 (41.7) | 8^a^ (22.2) | 21 (58.3) | 7 (19.4) | 8 (22.2) |
|  | PBO | 101 | 29 (28.7) | 6 (5.9) | 29 | | 6 (20.7) | 3 (10.3)^d^ | 23 (79.3) | 3 (10.3) | 3 (10.3) |
| **VARSITY** | VDZ 300mg Q8W | 79 | 44 (55.7) | 24 (30.4) | 44 | | 24 (54.5)^c^ | 16 (36.4)^f^ | 20 (45.4) | 8 (18.2) | 16 (36.4) |
|  | ADA 160/80/40mg Q2W | 81 | 26 (32.1) | 13 (16.0) | 26 | | 13 (50.0)^c^ | 13 (50.0)^f^ | 13 (50.0) | 0 (0.0) | 13 (50.0) |
| ^a^ Remission in induction phase responders, data from^49^  ^b^ Data from Varsity were published in the poster “Comparing the efficacy and safety of subcutaneous vedolizumab vs adalimumab for the treatment of ulcerative colitis: a network meta-analysis ” at advance in inflammatory bowel diseases conference 2019.  ^c^ Maintenance sustained response not published. Percentages of patients with sustained response provided by oral presentation at conference and used to estimate n’s.  ^d^ Maintenance remission from trial data not provided therefore we applied the weighted average PBO data for % of responders who were remitters from the combined PBO arms of the other trials (58%) leading to the estimate of 14 (naïve) and 3 (experienced) remitters in the PBO arm.  ^e^ Maintenance remission from trial data provided an illogical value (n = 20) which is greater than sustained responders (n =17), therefore we applied the weighted average PBO data for % of responders who were remitters from the combined PBO arms of the other trials (58%) leading to the estimate of 10 remitters in the PBO arm.  ^f^ Maintenance remission n not published. Estimated from maintenance remissiion % of 34.2% (naïve, VDZ), 24.3% (naïve, ADA), 20.3% (experienced, VDZ) and 16.0% (experienced, ADA).  ^g^ In scenario analysis the minimum (31.8%, SELECTION) and maximum (71.4%, GEMINI 1) placebo arm values were used to estimate this value, leading to estimates of 5 (min) and 12 (max).  ADA, adalimumab; IFX, infliximab; PBO, placebo; QXW, every X weeks; VDZ, vedolizumab | | | | | | | | | | | |

**Supplementary table 10.** Treat-through maintenance phase re-weighting using Hernandez *et al*. (sensitivity analysis).

|  |  | **Reported trial results** | | | | **Re-weighting calculations** | | | | | | |
| --- | --- | --- | --- | --- | --- | --- | --- | --- | --- | --- | --- | --- |
| **Bold = numbers used in NMA** |  | **(1) Start N** | **(2) Induction Responders/ Remitters** | **(3) Maintenance responders/ remitters** | **(4) Sustained Responders/ remitters^c^** | **(5) Proportion induction Response/ remission (2/1)^a^** | **(6)**  **Imputed Placebo responders/ remitters (6 = 5*1^b^** | **(7)**  **Maintenance responders/ remitters who were not sustained responders (3-4)** | **(8)**  **Lower bound estimate for proportion of (7) who may have responded/ remitted on active therapy induction (6-2)/(1-2)** | **(9)**  **Lower bound estimate n additional sustained responders (8*7)** | **(10)**  **Mean of lower bound estimate of n additional sustained responders (9) and upper bound estimate (7)** | **(11)**  **Imputed sustained responders/ remitters (4 + 10)^c^** |
| **Biologic-naïve: ULTRA 2 (ADA vs. PBO)** | | | | | | | | | | | | |
| Response | Active treatment arm | 150 | **89** | 55 | **44** | 59.3% | NA | NA | NA | NA | NA | NA |
|  | Placebo | 145 | 56 | 35 | 24 |  | **86**^c^ | 11 | 34% | 4 | 7 | **31**^c^ |
| Remission | Active treatment arm | 150 | **32** | **28**^d^ | 16 | 21.3% | NA | NA | NA | NA | NA | NA |
|  | Placebo | 145 | 16 | **18**^c^ | 9 |  | NA | NA | NA | NA | NA | NA |
| **Biologic-naïve: ACT 1 (IFX vs. PBO)** | | | | | | | | | | | | |
| Response | Active treatment arm | 121 | **84** | 55 | **47** | 69.4% | NA | NA | NA | NA | NA | NA |
|  | Placebo | 121 | 45 | 24 | 17 |  | **84**^c^ | 7 | 51% | 4 | 5 | **22**^c^ |
| Remission | Active treatment arm | 121 | **47** | **42** | **24** | 38.8% | NA | NA | NA | NA | NA | NA |
|  | Placebo | 121 | 18 | **20**^c^ | 8 |  | NA | NA | NA | NA | NA | NA |
| **Biologic-experienced: ULTRA 2 (ADA vs. PBO)** | | | | | | | | | | | | |
| Response | Active treatment arm | 98 | **36** | 20 | **15** | 36.7% | NA | NA | NA | NA | NA | NA |
|  | Placebo | 101 | 29 | 10 | 6 |  | **37**^c^ | 4 | 11% | 0 | 2 | **8**^c^ |
| Remission | Active treatment arm | 98 | **9** | **8**^d^ | 5 | 9.2% | NA | NA | NA | NA | NA | NA |
|  | Placebo | 101 | 7 | **3**^c^ | 1 |  | NA | NA | NA | NA | NA | NA |
| ^a^ active treatment arm % assumed to apply to placebo arm for the purpose of this calculation.  ^b^ only imputed for placebo arm.  ^c^ note that for placebo arms all numbers used in final NMA inputs were reduced by 10% to increase the measure of uncertainty of outputs in line with the imputation method.  ^d^ note based on additional data available in subsequent abstract for treatment arms only: proportion of patients with 8-week response who were 52 week remitters.  ADA, adalimumab; IFX, infliximab; PBO, placebo; | | | | | | | | | | | | |

## League tables of network analysis results

### Induction

**Supplementary table 11.** Relative risk of achieving response (including remission) at induction in biologic-naïve patients (fixed-effects model)

| Relative risk of response at induction – posterior median (95% CrI) | | | | | | | | |
| --- | --- | --- | --- | --- | --- | --- | --- | --- |
| **PBO** | 1.51 (1.43, 1.60) | 1.34 (1.24, 1.44) | 1.62 (1.51, 1.74) | 1.69 (1.59, 1.78) | 2.09 (2.02, 2.17) | 1.77 (1.66, 1.90) | 1.86 (1.74, 1.98) | 2.01 (1.91, 2.11) |
| 0.66 (0.63, 0.70) | **ADA 160/80/40 mg** | 0.88 (0.81, 0.97) | 1.07 (0.98, 1.17) | 1.11 (1.03, 1.20) | 1.38 (1.30, 1.47) | 1.17 (1.08, 1.28) | 1.23 (1.13, 1.34) | 1.33 (1.27, 1.39) |
| 0.75 (0.69, 0.81) | 1.13 (1.03, 1.24) | **FIL 100 mg** | 1.21 (1.14, 1.29) | 1.26 (1.14, 1.39) | 1.56 (1.44, 1.71) | 1.32 (1.19, 1.47) | 1.39 (1.26, 1.53) | 1.50 (1.37, 1.65) |
| 0.62 (0.58, 0.66) | 0.93 (0.86, 1.02) | 0.82 (0.77, 0.88) | **FIL 200 mg** | 1.04 (0.95, 1.14) | 1.29 (1.20, 1.40) | 1.09 (0.99, 1.21) | 1.15 (1.05, 1.26) | 1.24 (1.14, 1.35) |
| 0.59 (0.56, 0.63) | 0.90 (0.83, 0.97) | 0.79 (0.72, 0.88) | 0.96 (0.88, 1.06) | **GOL 200/100 mg** | 1.24 (1.16, 1.33) | 1.05 (0.97, 1.15) | 1.11 (1.01, 1.20) | 1.20 (1.11, 1.29) |
| 0.48 (0.46, 0.50) | 0.72 (0.68, 0.77) | 0.64 (0.58, 0.70) | 0.78 (0.72, 0.84) | 0.81 (0.75, 0.86) | **IFX 5 mg/kg** | 0.85 (0.79, 0.91) | 0.89 (0.82, 0.95) | 0.96 (0.91, 1.02) |
| 0.56 (0.53, 0.60) | 0.85 (0.78, 0.93) | 0.75 (0.68, 0.84) | 0.91 (0.83, 1.01) | 0.95 (0.87, 1.04) | 1.18 (1.10, 1.27) | **TOFA 10 mg** | 1.05 (0.96, 1.15) | 1.13 (1.04, 1.23) |
| 0.54 (0.51, 0.57) | 0.81 (0.75, 0.89) | 0.72 (0.65, 0.79) | 0.87 (0.79, 0.96) | 0.90 (0.83, 0.99) | 1.12 (1.05, 1.21) | 0.96 (0.87, 1.05) | **UST 6 mg/kg** | 1.08 (0.99, 1.17) |
| 0.50 (0.47, 0.52) | 0.75 (0.72, 0.79) | 0.66 (0.61, 0.73) | 0.80 (0.74, 0.88) | 0.84 (0.78, 0.90) | 1.04 (0.98, 1.10) | 0.88 (0.81, 0.96) | 0.92 (0.85, 1.01) | **VDZ 300 mg** |
| *ADA: adalimumab; FIL: filgotinib; GOL: golimumab; IFX: infliximab; TOFA: tofacitinib; UST: ustekinumab; VDZ: vedolizumab*  *Pink: Relative risk > 1 (column treatment has greater RR of achieving the outcome than row treatment, if CrI does not include 1 this difference is significant)*  *Blue: Relative risk < 1*  *Each cell represents the comparison (median relative risk and 95% CrI) of the column treatment versus the row treatment.* | | | | | | | | |

**Supplementary table 12.** Relative risk of achieving response (including remission) at induction in biologic-experienced patients (fixed-effects model)

| Relative risk of response at induction – posterior median (95% CrI) | | | | | | |
| --- | --- | --- | --- | --- | --- | --- |
| **PBO** | 1.28 (1.08, 1.48) | 2.13 (1.88, 2.39) | 3.28 (2.99, 3.60) | 2.88 (2.62, 3.16) | 2.93 (2.65, 3.22) | 2.61 (2.28, 2.96) |
| 0.78 (0.67, 0.92) | **ADA 160/80/40mg** | 1.67 (1.36, 2.04) | 2.58 (2.14, 3.10) | 2.25 (1.87, 2.73) | 2.28 (1.90, 2.76) | 2.04 (1.76, 2.36) |
| 0.47 (0.42, 0.53) | 0.60 (0.49, 0.73) | **FIL 100mg** | 1.54 (1.42, 1.67) | 1.36 (1.17, 1.58) | 1.37 (1.18, 1.60) | 1.22 (1.02, 1.45) |
| 0.31 (0.28, 0.33) | 0.39 (0.32, 0.47) | 0.65 (0.60, 0.70) | **FIL 200mg** | 0.88 (0.77, 1.00) | 0.89 (0.78, 1.02) | 0.79 (0.67, 0.93) |
| 0.35 (0.32, 0.38) | 0.44 (0.37, 0.54) | 0.74 (0.63, 0.85) | 1.13 (1.00, 1.29) | **TOFA 10mg** | 1.02 (0.88, 1.16) | 0.90 (0.77, 1.06) |
| 0.34 (0.31, 0.38) | 0.44 (0.36, 0.53) | 0.73 (0.62, 0.85) | 1.13 (0.98, 1.28) | 0.98 (0.86, 1.13) | **UST 6mg/kg** | 0.89 (0.76, 1.04) |
| 0.38 (0.34, 0.44) | 0.49 (0.42, 0.57) | 0.82 (0.69, 0.98) | 1.26 (1.08, 1.48) | 1.11 (0.95, 1.31) | 1.12 (0.96, 1.32) | **VDZ 300mg** |
| ADA: adalimumab; FIL: filgotinib; PBO: placebo; TOFA: tofacitinib; UST: ustekinumab; VDZ: vedolizumab  *Pink: Relative risk >1 (column treatment has greater RR of achieving the outcome than row treatment, if CrI does not include 1 this difference is significant)*  *Blue: Relative risk <1*  *Each cell represents the comparison (median relative risk and 95% CrI) of the column treatment versus the row treatment* | | | | | | |

**Supplementary table 13.** Relative risk of achieving remission at induction in biologic-naïve patients (fixed-effects model)

| Relative risk of response at induction – posterior median (95% CrI) | | | | | | | | |
| --- | --- | --- | --- | --- | --- | --- | --- | --- |
| **PBO** | 2.05 (1.86, 2.27) | 1.65 (1.43, 1.88) | 2.33 (2.04, 2.66) | 2.51 (2.24, 2.79) | 3.83 (3.56, 4.10) | 2.75 (2.42, 3.14) | 3.04 (2.66, 3.42) | 3.54 (3.20, 3.90) |
| 0.49 (0.44, 0.54) | **ADA 160/80/40 mg** | 0.80 (0.68, 0.95) | 1.14 (0.96, 1.33) | 1.22 (1.05, 1.40) | 1.87 (1.66, 2.09) | 1.35 (1.15, 1.58) | 1.48 (1.25, 1.73) | 1.72 (1.58, 1.87) |
| 0.61 (0.53, 0.70) | 1.25 (1.06, 1.47) | **FIL 100 mg** | 1.42 (1.27, 1.58) | 1.53 (1.27, 1.82) | 2.32 (2.00, 2.73) | 1.67 (1.37, 2.04) | 1.83 (1.53, 2.20) | 2.16 (1.82, 2.54) |
| 0.43 (0.38, 0.49) | 0.88 (0.75, 1.04) | 0.70 (0.63, 0.79) | **FIL 200 mg** | 1.07 (0.90, 1.28) | 1.64 (1.42, 1.90) | 1.18 (0.98, 1.43) | 1.30 (1.09, 1.56) | 1.52 (1.29, 1.78) |
| 0.40 (0.36, 0.45) | 0.82 (0.71, 0.95) | 0.66 (0.55, 0.79) | 0.93 (0.78, 1.11) | **GOL 200/100 mg** | 1.53 (1.35, 1.74) | 1.11 (0.93, 1.31) | 1.22 (1.02, 1.43) | 1.41 (1.23, 1.64) |
| 0.26 (0.24, 0.28) | 0.54 (0.48, 0.60) | 0.43 (0.37, 0.50) | 0.61 (0.53, 0.70) | 0.66 (0.58, 0.74) | **IFX 5 mg/kg** | 0.72 (0.62, 0.83) | 0.79 (0.68, 0.91) | 0.92 (0.82, 1.04) |
| 0.36 (0.32, 0.41) | 0.74 (0.63, 0.87) | 0.60 (0.49, 0.73) | 0.85 (0.70, 1.02) | 0.90 (0.76, 1.07) | 1.38 (1.20, 1.60) | **TOFA 10 mg** | 1.09 (0.92, 1.31) | 1.28 (1.09, 1.51) |
| 0.33 (0.29, 0.38) | 0.68 (0.58, 0.80) | 0.55 (0.45, 0.65) | 0.77 (0.64, 0.92) | 0.82 (0.70, 0.98) | 1.26 (1.10, 1.47) | 0.92 (0.76, 1.09) | **UST 6 mg/kg** | 1.17 (0.99, 1.37) |
| 0.28 (0.26, 0.31) | 0.58 (0.53, 0.63) | 0.46 (0.39, 0.55) | 0.66 (0.56, 0.77) | 0.71 (0.61, 0.81) | 1.08 (0.96, 1.22) | 0.78 (0.66, 0.92) | 0.86 (0.73, 1.01) | **VDZ 300 mg** |
| *ADA: adalimumab; FIL: filgotinib; GOL: golimumab; IFX: infliximab; TOFA: tofacitinib; UST: ustekinumab; VDZ: vedolizumab*  *Pink: Relative risk >1 (column treatment has greater RR of achieving the outcome than row treatment, if CrI does not include 1 this difference is significant)*  *Blue: Relative risk <1*  *Each cell represents the comparison (median relative risk and 95% CrI) of the column treatment versus the row treatment* | | | | | | | | |

**Supplementary table 14.** Relative risk of achieving remission at induction in biologic-experienced patients (fixed-effects model)

| Relative risk of remission at induction – posterior median (95% CrI) | | | | | | |
| --- | --- | --- | --- | --- | --- | --- |
| **PBO** | 1.49 (1.14, 1.91) | 3.58 (2.88, 4.38) | 7.94 (6.61, 9.47) | 6.22 (5.20, 7.38) | 6.37 (5.27, 7.67) | 5.13 (4.02, 6.48) |
| 0.67 (0.52, 0.88) | **ADA 160/80/40mg** | 2.40 (1.71, 3.37) | 5.36 (3.90, 7.36) | 4.14 (3.01, 5.79) | 4.26 (3.11, 5.84) | 3.46 (2.68, 4.41) |
| 0.28 (0.23, 0.35) | 0.42 (0.30, 0.59) | **FIL 100mg** | 2.21 (1.92, 2.59) | 1.74 (1.33, 2.29) | 1.77 (1.36, 2.37) | 1.43 (1.04, 1.95) |
| 0.13 (0.11, 0.15) | 0.19 (0.14, 0.26) | 0.45 (0.39, 0.52) | **FIL 200mg** | 0.79 (0.61, 1.00) | 0.80 (0.62, 1.04) | 0.65 (0.48, 0.86) |
| 0.16 (0.14, 0.19) | 0.24 (0.17, 0.33) | 0.58 (0.44, 0.75) | 1.27 (1.00, 1.63) | **TOFA 10mg** | 1.03 (0.79, 1.32) | 0.83 (0.61, 1.11) |
| 0.16 (0.13, 0.19) | 0.23 (0.17, 0.32) | 0.57 (0.42, 0.74) | 1.25 (0.97, 1.61) | 0.97 (0.76, 1.26) | **UST 6mg/kg** | 0.80 (0.60, 1.08) |
| 0.19 (0.15, 0.25) | 0.29 (0.23, 0.37) | 0.70 (0.51, 0.96) | 1.54 (1.16, 2.09) | 1.21 (0.90, 1.64) | 1.25 (0.93, 1.67) | **VDZ 300mg** |
| ADA: adalimumab; FIL: filgotinib; PBO: placebo; TOFA: tofacitinib; UST: ustekinumab; VDZ: vedolizumab  *Pink: Relative risk >1 (column treatment has greater RR of achieving the outcome than row treatment, if CrI does not include 1 this difference is significant)*  *Blue: Relative risk <1*  *Each cell represents the comparison (median relative risk and 95% CrI) of the column treatment versus the row treatment* | | | | | | |

**Supplementary table 15.** Relative risk of endoscopic mucosal healing at induction in biologic-naïve patients (fixed-effects model)

| Relative risk of response at induction – posterior median (95% CrI) | | | | | | | | |
| --- | --- | --- | --- | --- | --- | --- | --- | --- |
| PBO | 1.32 (1.23, 1.42) | 1.27 (1.13, 1.42) | 1.57 (1.42, 1.73) | 1.45 (1.35, 1.56) | 1.94 (1.85, 2.03) | 1.61 (1.46, 1.76) | 1.52 (1.36, 1.67) | 1.90 (1.71, 2.09) |
| 0.76 (0.71, 0.81) | ADA 160/80/40mg | 0.96 (0.84, 1.10) | 1.19 (1.05, 1.34) | 1.10 (0.99, 1.22) | 1.47 (1.35, 1.60) | 1.22 (1.08, 1.38) | 1.15 (1.01, 1.29) | 1.44 (1.27, 1.63) |
| 0.79 (0.71, 0.89) | 1.04 (0.91, 1.19) | FIL 100mg | 1.24 (1.14, 1.34) | 1.15 (1.00, 1.31) | 1.53 (1.35, 1.74) | 1.27 (1.09, 1.47) | 1.19 (1.02, 1.39) | 1.50 (1.29, 1.74) |
| 0.64 (0.58, 0.70) | 0.84 (0.74, 0.95) | 0.81 (0.75, 0.87) | FIL 200mg | 0.92 (0.82, 1.05) | 1.23 (1.11, 1.38) | 1.02 (0.89, 1.17) | 0.96 (0.83, 1.11) | 1.21 (1.04, 1.39) |
| 0.69 (0.64, 0.74) | 0.91 (0.82, 1.01) | 0.87 (0.76, 1.00) | 1.09 (0.96, 1.22) | GOL 200/100mg | 1.33 (1.22, 1.45) | 1.10 (0.98, 1.24) | 1.04 (0.92, 1.17) | 1.30 (1.16, 1.48) |
| 0.52 (0.49, 0.54) | 0.68 (0.63, 0.74) | 0.66 (0.58, 0.74) | 0.81 (0.72, 0.90) | 0.75 (0.69, 0.82) | IFX 5mg/kg | 0.83 (0.75, 0.92) | 0.78 (0.70, 0.87) | 0.98 (0.87, 1.10) |
| 0.62 (0.57, 0.68) | 0.82 (0.73, 0.93) | 0.79 (0.68, 0.92) | 0.98 (0.86, 1.12) | 0.91 (0.80, 1.02) | 1.20 (1.09, 1.34) | TOFA 10mg | 0.94 (0.82, 1.08) | 1.18 (1.02, 1.36) |
| 0.66 (0.60, 0.73) | 0.87 (0.78, 0.99) | 0.84 (0.72, 0.98) | 1.04 (0.90, 1.20) | 0.96 (0.85, 1.09) | 1.28 (1.15, 1.44) | 1.06 (0.92, 1.22) | UST 6mg/kg | 1.26 (1.09, 1.45) |
| 0.52 (0.48, 0.58) | 0.70 (0.61, 0.79) | 0.67 (0.58, 0.78) | 0.83 (0.72, 0.96) | 0.77 (0.68, 0.87) | 1.02 (0.91, 1.14) | 0.85 (0.74, 0.98) | 0.80 (0.69, 0.92) | VDZ 300mg |
| *ADA: adalimumab; FIL: filgotinib; GOL: golimumab; IFX: infliximab; TOFA: tofacitinib; UST: ustekinumab; VDZ: vedolizumab*  *Pink: Relative risk >1 (column treatment has greater RR of achieving the outcome than row treatment, if CrI does not include 1 this difference is significant)*  *Blue: Relative risk <1*  *Each cell represents the comparison (median relative risk and 95% CrI) of the column treatment versus the row treatment* | | | | | | | | |

**Supplementary table 16.** Relative risk of endoscopic mucosal healing at induction in biologic-experienced patients (fixed-effects model)

| Relative risk of response at induction – posterior median (95% CrI) | | | | | | |
| --- | --- | --- | --- | --- | --- | --- |
| PBO | 1.08 (0.90, 1.30) | 1.63 (1.32, 2.01) | 2.09 (1.72, 2.55) | 3.09 (2.52, 3.86) | 2.75 (2.25, 3.39) | 1.54 (1.25, 1.95) |
| 0.92 (0.77, 1.11) | ADA 160/80/40mg | 1.49 (1.14, 2.02) | 1.91 (1.46, 2.56) | 2.87 (2.18, 3.84) | 2.53 (1.94, 3.35) | 1.42 (1.08, 1.91) |
| 0.61 (0.50, 0.76) | 0.67 (0.49, 0.88) | FIL 100mg | 1.28 (1.13, 1.46) | 1.87 (1.44, 2.51) | 1.67 (1.29, 2.19) | 0.95 (0.70, 1.29) |
| 0.48 (0.39, 0.58) | 0.52 (0.39, 0.68) | 0.78 (0.69, 0.88) | FIL 200mg | 1.47 (1.15, 1.90) | 1.30 (1.03, 1.66) | 0.74 (0.56, 0.99) |
| 0.32 (0.26, 0.40) | 0.35 (0.26, 0.46) | 0.53 (0.40, 0.70) | 0.68 (0.53, 0.87) | TOFA 10mg | 0.90 (0.71, 1.11) | 0.51 (0.38, 0.66) |
| 0.36 (0.30, 0.44) | 0.39 (0.30, 0.52) | 0.60 (0.46, 0.78) | 0.77 (0.60, 0.97) | 1.11 (0.90, 1.40) | UST 6mg/kg | 0.57 (0.43, 0.74) |
| 0.65 (0.51, 0.80) | 0.70 (0.52, 0.93) | 1.05 (0.78, 1.43) | 1.35 (1.01, 1.80) | 1.97 (1.52, 2.65) | 1.75 (1.35, 2.33) | VDZ 300mg |
| ADA: adalimumab; FIL: filgotinib; TOFA: tofacitinib; UST: ustekinumab; VDZ: vedolizumab  *Pink: Relative risk >1 (column treatment has greater RR of achieving the outcome than row treatment, if CrI does not include 1 this difference is significant)*  *Blue: Relative risk <1*  *Each cell represents the comparison (median relative risk and 95% CrI) of the column treatment versus the row treatment* | | | | | | |

### Maintenance Phase

**Supplementary table 17.** Relative risk of achieving response (including remission) at maintenance phase in biologic-naïve patients (fixed-effects model)

| Relative risk of response at induction – posterior median (95% CrI) | | | | | | | | | | | | | |
| --- | --- | --- | --- | --- | --- | --- | --- | --- | --- | --- | --- | --- | --- |
| **PBO** | 1.60 (1.45, 1.77) | 1.21 (1.05, 1.37) | 2.05 (1.82, 2.37) | 1.54 (1.41, 1.70) | 1.49 (1.36, 1.64) | 1.75 (1.53, 2.00) | 2.28 (2.01, 2.64) | 2.13 (1.89, 2.44) | 1.69 (1.51, 1.90) | 1.70 (1.52, 1.91) | 2.03 (1.77, 2.35) | 1.90 (1.69, 2.15) | 1.90 (1.72, 2.14) |
| 0.63 (0.56, 0.69) | **ADA 160/80/40mg** | 0.76 (0.64, 0.87) | 1.28 (1.15, 1.44) | 0.97 (0.87, 1.07) | 0.93 (0.84, 1.04) | 1.10 (0.96, 1.25) | 1.41 (1.28, 1.59) | 1.33 (1.19, 1.48) | 1.06 (0.94, 1.19) | 1.06 (0.95, 1.20) | 1.27 (1.14, 1.42) | 1.18 (1.08, 1.31) | 1.18 (1.11, 1.27) |
| 0.83 (0.73, 0.95) | 1.32 (1.14, 1.57) | **FIL 100mg** | 1.70 (1.45, 2.05) | 1.28 (1.10, 1.50) | 1.23 (1.07, 1.44) | 1.44 (1.22, 1.75) | 1.88 (1.60, 2.27) | 1.76 (1.51, 2.10) | 1.40 (1.20, 1.64) | 1.40 (1.20, 1.67) | 1.68 (1.42, 2.03) | 1.58 (1.34, 1.87) | 1.57 (1.35, 1.87) |
| 0.49 (0.42, 0.55) | 0.78 (0.69, 0.87) | 0.59 (0.49, 0.69) | **FIL 200mg** | 0.76 (0.66, 0.84) | 0.73 (0.65, 0.81) | 0.86 (0.74, 0.97) | 1.10 (1.01, 1.22) | 1.03 (0.94, 1.14) | 0.83 (0.73, 0.92) | 0.83 (0.73, 0.93) | 0.99 (0.88, 1.10) | 0.93 (0.83, 1.03) | 0.93 (0.84, 1.01) |
| 0.65 (0.59, 0.71) | 1.03 (0.93, 1.15) | 0.78 (0.67, 0.91) | 1.32 (1.19, 1.50) | **GOL 100mg Q4W** | 0.97 (0.90, 1.04) | 1.13 (0.99, 1.29) | 1.46 (1.32, 1.66) | 1.37 (1.23, 1.54) | 1.09 (0.98, 1.22) | 1.10 (0.98, 1.24) | 1.31 (1.16, 1.50) | 1.23 (1.10, 1.38) | 1.22 (1.11, 1.36) |
| 0.67 (0.61, 0.74) | 1.07 (0.96, 1.19) | 0.81 (0.69, 0.94) | 1.37 (1.23, 1.55) | 1.03 (0.96, 1.12) | **GOL 50mg Q4W** | 1.17 (1.02, 1.35) | 1.52 (1.36, 1.73) | 1.42 (1.27, 1.60) | 1.13 (1.01, 1.28) | 1.14 (1.01, 1.29) | 1.36 (1.20, 1.56) | 1.27 (1.14, 1.44) | 1.27 (1.15, 1.42) |
| 0.57 (0.50, 0.65) | 0.91 (0.80, 1.04) | 0.69 (0.57, 0.82) | 1.17 (1.03, 1.35) | 0.88 (0.77, 1.01) | 0.85 (0.74, 0.98) | **IFX 5mg/kg** | 1.29 (1.14, 1.49) | 1.20 (1.07, 1.40) | 0.97 (0.84, 1.10) | 0.97 (0.85, 1.11) | 1.16 (1.00, 1.35) | 1.08 (0.95, 1.25) | 1.08 (0.96, 1.23) |
| 0.44 (0.38, 0.50) | 0.71 (0.63, 0.78) | 0.53 (0.44, 0.63) | 0.91 (0.82, 0.99) | 0.68 (0.60, 0.76) | 0.66 (0.58, 0.74) | 0.78 (0.67, 0.88) | **TOFA 10mg** | 0.94 (0.88, 0.99) | 0.75 (0.66, 0.83) | 0.76 (0.66, 0.84) | 0.90 (0.80, 1.00) | 0.84 (0.75, 0.92) | 0.84 (0.76, 0.91) |
| 0.47 (0.41, 0.53) | 0.75 (0.67, 0.84) | 0.57 (0.48, 0.66) | 0.97 (0.88, 1.07) | 0.73 (0.65, 0.81) | 0.71 (0.62, 0.78) | 0.83 (0.72, 0.93) | 1.07 (1.01, 1.13) | **TOFA 5mg** | 0.80 (0.71, 0.89) | 0.80 (0.71, 0.89) | 0.96 (0.86, 1.06) | 0.90 (0.81, 0.99) | 0.90 (0.82, 0.97) |
| 0.59 (0.53, 0.66) | 0.94 (0.84, 1.07) | 0.72 (0.61, 0.84) | 1.21 (1.09, 1.37) | 0.91 (0.82, 1.02) | 0.88 (0.78, 0.99) | 1.04 (0.91, 1.19) | 1.34 (1.20, 1.51) | 1.25 (1.13, 1.41) | **UST 90mg Q12W** | 1.00 (0.92, 1.10) | 1.20 (1.06, 1.37) | 1.12 (1.00, 1.27) | 1.12 (1.01, 1.25) |
| 0.59 (0.52, 0.66) | 0.94 (0.83, 1.06) | 0.71 (0.60, 0.83) | 1.21 (1.07, 1.36) | 0.91 (0.81, 1.03) | 0.88 (0.78, 0.99) | 1.03 (0.90, 1.18) | 1.32 (1.20, 1.51) | 1.24 (1.12, 1.40) | 1.00 (0.91, 1.08) | **UST 90mg Q8W** | 1.19 (1.05, 1.37) | 1.11 (0.99, 1.26) | 1.11 (1.00, 1.24) |
| 0.49 (0.43, 0.56) | 0.79 (0.70, 0.88) | 0.59 (0.49, 0.71) | 1.01 (0.91, 1.14) | 0.76 (0.67, 0.86) | 0.73 (0.64, 0.84) | 0.86 (0.74, 1.00) | 1.11 (1.00, 1.25) | 1.04 (0.94, 1.17) | 0.83 (0.73, 0.95) | 0.84 (0.73, 0.95) | **VDZ 108mg SC Q2W** | 0.93 (0.84, 1.05) | 0.93 (0.85, 1.03) |
| 0.53 (0.46, 0.59) | 0.84 (0.76, 0.92) | 0.63 (0.53, 0.74) | 1.07 (0.97, 1.21) | 0.81 (0.73, 0.91) | 0.79 (0.69, 0.88) | 0.93 (0.80, 1.05) | 1.19 (1.08, 1.33) | 1.11 (1.01, 1.23) | 0.89 (0.79, 1.00) | 0.90 (0.79, 1.01) | 1.07 (0.96, 1.19) | **VDZ 300mg Q4W** | 1.00 (0.92, 1.08) |
| 0.53 (0.47, 0.58) | 0.84 (0.79, 0.90) | 0.64 (0.53, 0.74) | 1.08 (0.99, 1.19) | 0.82 (0.74, 0.90) | 0.79 (0.70, 0.87) | 0.93 (0.81, 1.05) | 1.19 (1.10, 1.31) | 1.12 (1.03, 1.22) | 0.89 (0.80, 0.99) | 0.90 (0.80, 1.00) | 1.07 (0.97, 1.17) | 1.00 (0.93, 1.08) | **VDZ 300mg Q8W** |
| *ADA: adalimumab; FIL: filgotinib; GOL: golimumab; IFX: infliximab; TOFA: tofacitinib; UST: ustekinumab; VDZ: vedolizumab*  *Pink: Relative risk >1 (column treatment has greater RR of achieving the outcome than row treatment, if CrI does not include 1 this difference is significant)*  *Blue: Relative risk <1*  *Each cell represents the comparison (median relative risk and 95% CrI) of the column treatment versus the row treatment* | | | | | | | | | | | | | |

**Supplementary table 18.** Relative risk of achieving response (including remission) at maintenance phase in biologic-experienced patients (fixed-effects model)

| Relative risk of response at induction – posterior median (95% CrI) | | | | | | | | | | |
| --- | --- | --- | --- | --- | --- | --- | --- | --- | --- | --- |
| **PBO** | 3.25 (2.60, 4.06) | 2.30 (1.79, 2.97) | 3.28 (2.65, 4.10) | 4.58 (3.77, 5.63) | 3.19 (2.65, 3.88) | 1.70 (1.42, 2.08) | 2.67 (2.25, 3.21) | 4.25 (3.28, 5.52) | 3.30 (2.62, 4.18) | 3.56 (2.89, 4.38) |
| 0.31 (0.25, 0.39) | **ADA 160/80/40mg** | 0.72 (0.53, 0.97) | 1.01 (0.80, 1.31) | 1.39 (1.13, 1.76) | 0.97 (0.77, 1.25) | 0.53 (0.40, 0.68) | 0.82 (0.65, 1.04) | 1.31 (1.02, 1.68) | 1.02 (0.80, 1.27) | 1.09 (0.92, 1.30) |
| 0.43 (0.34, 0.56) | 1.39 (1.03, 1.89) | **FIL 100mg** | 1.41 (1.06, 1.90) | 1.94 (1.51, 2.66) | 1.36 (1.05, 1.82) | 0.74 (0.54, 1.02) | 1.15 (0.87, 1.53) | 1.83 (1.32, 2.53) | 1.41 (1.05, 1.94) | 1.53 (1.15, 2.06) |
| 0.31 (0.24, 0.38) | 0.99 (0.76, 1.26) | 0.71 (0.53, 0.95) | **FIL 200mg** | 1.38 (1.13, 1.70) | 0.96 (0.77, 1.21) | 0.52 (0.40, 0.68) | 0.81 (0.64, 1.01) | 1.29 (0.99, 1.69) | 1.01 (0.78, 1.30) | 1.07 (0.86, 1.35) |
| 0.22 (0.18, 0.27) | 0.72 (0.57, 0.89) | 0.51 (0.38, 0.66) | 0.73 (0.59, 0.88) | **TOFA 10mg** | 0.70 (0.61, 0.80) | 0.37 (0.29, 0.48) | 0.59 (0.48, 0.71) | 0.94 (0.74, 1.19) | 0.74 (0.57, 0.91) | 0.78 (0.64, 0.94) |
| 0.31 (0.26, 0.38) | 1.03 (0.80, 1.30) | 0.73 (0.55, 0.95) | 1.04 (0.82, 1.29) | 1.42 (1.25, 1.65) | **TOFA 5mg** | 0.53 (0.42, 0.68) | 0.84 (0.68, 1.03) | 1.34 (1.03, 1.73) | 1.05 (0.80, 1.33) | 1.11 (0.89, 1.38) |
| 0.59 (0.48, 0.70) | 1.90 (1.46, 2.48) | 1.35 (0.98, 1.85) | 1.91 (1.47, 2.52) | 2.67 (2.10, 3.45) | 1.87 (1.47, 2.39) | **UST 90mg Q12W** | 1.56 (1.32, 1.85) | 2.46 (1.86, 3.36) | 1.92 (1.47, 2.55) | 2.06 (1.60, 2.68) |
| 0.37 (0.31, 0.44) | 1.22 (0.96, 1.55) | 0.87 (0.65, 1.15) | 1.24 (0.99, 1.55) | 1.71 (1.41, 2.09) | 1.19 (0.97, 1.48) | 0.64 (0.54, 0.76) | **UST 90mg Q8W** | 1.60 (1.22, 2.05) | 1.25 (0.96, 1.58) | 1.33 (1.08, 1.65) |
| 0.24 (0.18, 0.30) | 0.76 (0.59, 0.98) | 0.55 (0.40, 0.76) | 0.77 (0.59, 1.01) | 1.07 (0.84, 1.35) | 0.75 (0.58, 0.97) | 0.41 (0.30, 0.54) | 0.63 (0.49, 0.82) | **VDZ 108mg SC Q2W** | 0.79 (0.61, 0.99) | 0.84 (0.68, 1.02) |

**Supplementary table 19.** Relative risk of achieving remission at maintenance phase in biologic-naïve patients (fixed-effects model)

| Relative risk of remission at induction – posterior median (95% CrI) | | | | | | | | | | | | | |
| --- | --- | --- | --- | --- | --- | --- | --- | --- | --- | --- | --- | --- | --- |
| **PBO** | 1.92 (1.69, 2.20) | 1.30 (1.07, 1.54) | 2.80 (2.40, 3.37) | 1.83 (1.62, 2.08) | 1.74 (1.54, 1.98) | 2.20 (1.83, 2.65) | 3.29 (2.81, 3.95) | 2.95 (2.53, 3.51) | 2.09 (1.80, 2.44) | 2.10 (1.81, 2.48) | 2.77 (2.30, 3.34) | 2.50 (2.13, 2.92) | 2.47 (2.18, 2.87) |
| 0.52 (0.45, 0.59) | **ADA 160/80/40mg** | 0.67 (0.53, 0.83) | 1.46 (1.24, 1.72) | 0.95 (0.82, 1.10) | 0.91 (0.78, 1.06) | 1.14 (0.94, 1.39) | 1.70 (1.47, 2.00) | 1.53 (1.31, 1.80) | 1.09 (0.91, 1.29) | 1.10 (0.92, 1.30) | 1.43 (1.21, 1.69) | 1.29 (1.13, 1.49) | 1.28 (1.18, 1.41) |
| 0.77 (0.65, 0.93) | 1.48 (1.21, 1.89) | **FIL 100mg** | 2.17 (1.73, 2.79) | 1.42 (1.15, 1.75) | 1.34 (1.10, 1.67) | 1.69 (1.33, 2.22) | 2.53 (2.04, 3.24) | 2.28 (1.85, 2.91) | 1.61 (1.30, 2.02) | 1.62 (1.30, 2.05) | 2.14 (1.68, 2.75) | 1.92 (1.54, 2.44) | 1.92 (1.55, 2.41) |
| 0.36 (0.30, 0.42) | 0.68 (0.58, 0.81) | 0.46 (0.36, 0.58) | **FIL 200mg** | 0.66 (0.55, 0.77) | 0.62 (0.52, 0.73) | 0.79 (0.64, 0.95) | 1.17 (1.01, 1.36) | 1.05 (0.90, 1.22) | 0.74 (0.63, 0.88) | 0.75 (0.63, 0.90) | 0.98 (0.82, 1.17) | 0.90 (0.75, 1.04) | 0.89 (0.76, 1.02) |
| 0.55 (0.48, 0.62) | 1.05 (0.91, 1.22) | 0.70 (0.57, 0.87) | 1.52 (1.30, 1.83) | **GOL 100mg Q4W** | 0.95 (0.85, 1.06) | 1.20 (0.99, 1.46) | 1.78 (1.53, 2.13) | 1.61 (1.37, 1.90) | 1.14 (0.98, 1.34) | 1.15 (0.96, 1.37) | 1.51 (1.25, 1.83) | 1.36 (1.16, 1.59) | 1.35 (1.17, 1.57) |
| 0.58 (0.51, 0.65) | 1.10 (0.94, 1.29) | 0.74 (0.60, 0.91) | 1.61 (1.36, 1.91) | 1.05 (0.94, 1.17) | **GOL 50mg Q4W** | 1.26 (1.03, 1.55) | 1.87 (1.60, 2.25) | 1.69 (1.45, 2.00) | 1.20 (1.02, 1.42) | 1.21 (1.02, 1.44) | 1.59 (1.31, 1.92) | 1.43 (1.22, 1.70) | 1.42 (1.23, 1.66) |
| 0.45 (0.38, 0.55) | 0.87 (0.72, 1.06) | 0.59 (0.45, 0.75) | 1.27 (1.05, 1.57) | 0.83 (0.68, 1.01) | 0.79 (0.65, 0.97) | **IFX 5mg/kg** | 1.47 (1.23, 1.82) | 1.32 (1.11, 1.65) | 0.95 (0.78, 1.16) | 0.96 (0.78, 1.17) | 1.25 (1.00, 1.57) | 1.12 (0.92, 1.40) | 1.12 (0.93, 1.36) |
| 0.30 (0.25, 0.36) | 0.59 (0.50, 0.68) | 0.39 (0.31, 0.49) | 0.86 (0.73, 0.99) | 0.56 (0.47, 0.65) | 0.53 (0.45, 0.63) | 0.68 (0.55, 0.81) | **TOFA 10mg** | 0.90 (0.82, 0.98) | 0.64 (0.53, 0.75) | 0.65 (0.54, 0.75) | 0.84 (0.71, 0.99) | 0.76 (0.65, 0.88) | 0.76 (0.66, 0.86) |
| 0.34 (0.28, 0.40) | 0.65 (0.56, 0.76) | 0.44 (0.34, 0.54) | 0.95 (0.82, 1.11) | 0.62 (0.53, 0.73) | 0.59 (0.50, 0.69) | 0.76 (0.61, 0.90) | 1.11 (1.02, 1.21) | **TOFA 5mg** | 0.72 (0.60, 0.83) | 0.72 (0.60, 0.84) | 0.94 (0.79, 1.10) | 0.85 (0.72, 0.98) | 0.84 (0.74, 0.96) |
| 0.48 (0.41, 0.56) | 0.92 (0.78, 1.10) | 0.62 (0.49, 0.77) | 1.34 (1.13, 1.60) | 0.88 (0.74, 1.03) | 0.83 (0.70, 0.99) | 1.05 (0.87, 1.29) | 1.56 (1.34, 1.87) | 1.40 (1.20, 1.68) | **UST 90mg Q12W** | 1.00 (0.89, 1.14) | 1.32 (1.08, 1.61) | 1.19 (1.01, 1.43) | 1.18 (1.02, 1.39) |
| 0.48 (0.40, 0.55) | 0.91 (0.77, 1.08) | 0.62 (0.49, 0.77) | 1.33 (1.12, 1.59) | 0.87 (0.73, 1.04) | 0.83 (0.69, 0.98) | 1.05 (0.85, 1.28) | 1.54 (1.33, 1.86) | 1.39 (1.19, 1.66) | 1.00 (0.87, 1.13) | **UST 90mg Q8W** | 1.31 (1.08, 1.60) | 1.18 (0.99, 1.42) | 1.17 (1.00, 1.38) |
| 0.36 (0.30, 0.44) | 0.70 (0.59, 0.83) | 0.47 (0.36, 0.60) | 1.02 (0.86, 1.22) | 0.66 (0.55, 0.80) | 0.63 (0.52, 0.76) | 0.80 (0.64, 1.00) | 1.18 (1.01, 1.42) | 1.07 (0.91, 1.27) | 0.76 (0.62, 0.92) | 0.76 (0.63, 0.93) | **VDZ 108mg SC Q2W** | 0.90 (0.76, 1.07) | 0.89 (0.78, 1.04) |
| 0.40 (0.34, 0.47) | 0.78 (0.67, 0.89) | 0.52 (0.41, 0.65) | 1.12 (0.96, 1.33) | 0.74 (0.63, 0.86) | 0.70 (0.59, 0.82) | 0.89 (0.72, 1.08) | 1.31 (1.14, 1.54) | 1.18 (1.02, 1.38) | 0.84 (0.70, 0.99) | 0.85 (0.71, 1.01) | 1.11 (0.93, 1.32) | **VDZ 300mg Q4W** | 1.00 (0.89, 1.12) |
| 0.41 (0.35, 0.46) | 0.78 (0.71, 0.85) | 0.52 (0.41, 0.65) | 1.13 (0.98, 1.31) | 0.74 (0.64, 0.85) | 0.70 (0.60, 0.81) | 0.89 (0.73, 1.07) | 1.32 (1.17, 1.51) | 1.19 (1.04, 1.36) | 0.85 (0.72, 0.98) | 0.85 (0.72, 1.00) | 1.12 (0.96, 1.28) | 1.00 (0.89, 1.13) | **VDZ 300mg Q8W** |
| *ADA: adalimumab; FIL: filgotinib; GOL: golimumab; IFX: infliximab; TOFA: tofacitinib; UST: ustekinumab; VDZ: vedolizumab*  *Pink: Relative risk >1 (column treatment has greater RR of achieving the outcome than row treatment, if CrI does not include 1 this difference is significant)*  *Blue: Relative risk <1*  *Grey: Relative risk = 1*  *Each cell represents the comparison (median relative risk and 95% CrI) of the column treatment versus the row treatment* | | | | | | | | | | | | | |

**Supplementary table 20.** Relative risk of achieving remission at maintenance phase in biologic-experienced patients (fixed-effects model)

| Relative risk of remission at induction – posterior median (95% CrI) | | | | | | | | | | |
| --- | --- | --- | --- | --- | --- | --- | --- | --- | --- | --- |
| **PBO** | 4.84 (3.57, 6.47) | 3.02 (2.15, 4.20) | 4.92 (3.70, 6.54) | 7.88 (6.24, 10.19) | 4.72 (3.69, 6.03) | 2.00 (1.59, 2.60) | 3.67 (2.94, 4.64) | 7.15 (5.01, 10.11) | 4.95 (3.64, 6.76) | 5.47 (4.19, 7.21) |
| 0.21 (0.15, 0.28) | **ADA 160/80/40mg** | 0.63 (0.42, 0.96) | 1.02 (0.72, 1.47) | 1.60 (1.19, 2.23) | 0.96 (0.70, 1.37) | 0.42 (0.29, 0.59) | 0.76 (0.54, 1.05) | 1.48 (1.03, 2.12) | 1.03 (0.74, 1.42) | 1.14 (0.89, 1.45) |
| 0.33 (0.24, 0.47) | 1.58 (1.04, 2.40) | **FIL 100mg** | 1.62 (1.08, 2.42) | 2.55 (1.79, 3.87) | 1.54 (1.06, 2.28) | 0.67 (0.44, 1.03) | 1.22 (0.83, 1.78) | 2.35 (1.49, 3.67) | 1.63 (1.07, 2.51) | 1.80 (1.22, 2.70) |
| 0.20 (0.15, 0.27) | 0.98 (0.68, 1.38) | 0.62 (0.41, 0.93) | **FIL 200mg** | 1.59 (1.19, 2.16) | 0.95 (0.70, 1.31) | 0.41 (0.29, 0.59) | 0.74 (0.54, 1.02) | 1.46 (0.98, 2.14) | 1.02 (0.71, 1.46) | 1.10 (0.81, 1.53) |
| 0.13 (0.10, 0.16) | 0.62 (0.45, 0.84) | 0.39 (0.26, 0.56) | 0.63 (0.46, 0.84) | **TOFA 10mg** | 0.60 (0.50, 0.72) | 0.26 (0.18, 0.35) | 0.47 (0.35, 0.61) | 0.91 (0.64, 1.30) | 0.64 (0.45, 0.87) | 0.70 (0.53, 0.91) |
| 0.21 (0.17, 0.27) | 1.04 (0.73, 1.44) | 0.65 (0.44, 0.94) | 1.05 (0.76, 1.43) | 1.66 (1.40, 2.02) | **TOFA 5mg** | 0.43 (0.31, 0.59) | 0.79 (0.58, 1.04) | 1.53 (1.04, 2.21) | 1.08 (0.73, 1.50) | 1.16 (0.85, 1.58) |
| 0.50 (0.38, 0.63) | 2.39 (1.68, 3.43) | 1.50 (0.97, 2.29) | 2.42 (1.69, 3.49) | 3.91 (2.86, 5.48) | 2.35 (1.69, 3.24) | **UST 90mg Q12W** | 1.82 (1.45, 2.29) | 3.53 (2.37, 5.32) | 2.45 (1.71, 3.56) | 2.70 (1.92, 3.86) |
| 0.27 (0.22, 0.34) | 1.32 (0.95, 1.84) | 0.82 (0.56, 1.21) | 1.35 (0.98, 1.85) | 2.14 (1.64, 2.83) | 1.27 (0.96, 1.72) | 0.55 (0.44, 0.69) | **UST 90mg Q8W** | 1.95 (1.33, 2.78) | 1.37 (0.95, 1.90) | 1.48 (1.11, 2.02) |
| 0.14 (0.10, 0.20) | 0.68 (0.47, 0.97) | 0.43 (0.27, 0.67) | 0.68 (0.47, 1.02) | 1.10 (0.77, 1.55) | 0.65 (0.45, 0.96) | 0.28 (0.19, 0.42) | 0.51 (0.36, 0.75) | **VDZ 108mg SC Q2W** | 0.70 (0.49, 0.99) | 0.77 (0.57, 1.03) |
| 0.20 (0.15, 0.27) | 0.97 (0.71, 1.36) | 0.61 (0.40, 0.94) | 0.98 (0.68, 1.42) | 1.57 (1.15, 2.22) | 0.93 (0.67, 1.37) | 0.41 (0.28, 0.59) | 0.73 (0.53, 1.06) | 1.42 (1.01, 2.06) | **VDZ 300mg Q4W** | 1.10 (0.87, 1.42) |
| 0.18 (0.14, 0.24) | 0.88 (0.69, 1.12) | 0.55 (0.37, 0.82) | 0.91 (0.65, 1.23) | 1.44 (1.10, 1.90) | 0.86 (0.63, 1.18) | 0.37 (0.26, 0.52) | 0.67 (0.50, 0.90) | 1.31 (0.97, 1.75) | 0.91 (0.70, 1.15) | **VDZ 300mg Q8W** |
| ADA: adalimumab; FIL: filgotinib; TOFA: tofacitinib; UST: ustekinumab; VDZ: vedolizumab  *Pink : Relative risk >1 (column treatment has greater RR of achieving the outcome than row treatment, if CrI does not include 1 this difference is significant)*  *Blue: Relative risk <1*  *Each cell represents the comparison (median relative risk and 95% CrI) of the column treatment versus the row treatment* | | | | | | | | | | |

**Supplementary table 21.** Relative risk of endoscopic mucosal healing at maintenance phase in biologic naïve patients (fixed effects model)

| Relative risk of response at induction – posterior median (95% CrI) | | | | | | | | |
| --- | --- | --- | --- | --- | --- | --- | --- | --- |
| PBO | 1.71 (1.54, 1.86) | 1.30 (1.07, 1.55) | 2.67 (2.38, 2.95) | 2.34 (2.10, 2.55) | 1.84 (1.61, 2.04) | 1.91 (1.69, 2.15) | 2.50 (2.26, 2.73) | 2.39 (2.22, 2.56) |
| 0.59 (0.54, 0.65) | ADA 160/80/40mg | 0.76 (0.62, 0.93) | 1.56 (1.35, 1.79) | 1.37 (1.20, 1.56) | 1.08 (0.92, 1.25) | 1.13 (0.96, 1.31) | 1.46 (1.31, 1.64) | 1.40 (1.32, 1.49) |
| 0.77 (0.64, 0.94) | 1.31 (1.07, 1.61) | FIL 100mg | 2.04 (1.66, 2.54) | 1.80 (1.47, 2.23) | 1.41 (1.14, 1.76) | 1.48 (1.19, 1.84) | 1.92 (1.57, 2.34) | 1.85 (1.52, 2.25) |
| 0.37 (0.34, 0.42) | 0.64 (0.56, 0.74) | 0.49 (0.39, 0.60) | FIL 200mg | 0.87 (0.76, 1.01) | 0.69 (0.59, 0.80) | 0.71 (0.61, 0.85) | 0.94 (0.81, 1.08) | 0.90 (0.79, 1.02) |
| 0.43 (0.39, 0.48) | 0.73 (0.64, 0.84) | 0.56 (0.45, 0.68) | 1.15 (0.99, 1.32) | IFX 5mg/kg | 0.79 (0.68, 0.91) | 0.82 (0.71, 0.95) | 1.07 (0.94, 1.23) | 1.03 (0.91, 1.16) |
| 0.54 (0.49, 0.62) | 0.93 (0.80, 1.08) | 0.71 (0.57, 0.88) | 1.45 (1.24, 1.71) | 1.27 (1.10, 1.48) | UST 90mg Q12W | 1.05 (0.94, 1.17) | 1.36 (1.17, 1.58) | 1.30 (1.14, 1.50) |
| 0.52 (0.47, 0.59) | 0.89 (0.76, 1.04) | 0.67 (0.54, 0.84) | 1.40 (1.18, 1.64) | 1.22 (1.05, 1.41) | 0.96 (0.85, 1.07) | UST 90mg Q8W | 1.30 (1.12, 1.52) | 1.24 (1.09, 1.44) |
| 0.40 (0.37, 0.44) | 0.68 (0.61, 0.77) | 0.52 (0.43, 0.64) | 1.07 (0.92, 1.23) | 0.94 (0.82, 1.06) | 0.73 (0.63, 0.85) | 0.77 (0.66, 0.89) | VDZ 300mg Q4W | 0.96 (0.87, 1.05) |
| 0.42 (0.39, 0.45) | 0.71 (0.67, 0.76) | 0.54 (0.45, 0.66) | 1.11 (0.98, 1.26) | 0.97 (0.87, 1.10) | 0.77 (0.67, 0.87) | 0.80 (0.70, 0.92) | 1.04 (0.95, 1.14) | VDZ 300mg Q8W |
| *ADA: adalimumab; FIL: filgotinib; PBO: placebo; UST: ustekinumab; VDZ: vedolizumab*  *Pink: Relative risk >1 (column treatment has greater RR of achieving the outcome than row treatment, if CrI does not include 1 this difference is significant)*  *Blue: Relative risk <1*  *Each cell represents the comparison (median relative risk and 95% CrI) of the column treatment versus the row treatment* | | | | | | | | |

**Supplementary table 22.** Relative risk of endoscopic mucosal healing at maintenance phase in biologic-experienced patients (fixed-effects model)

| Relative risk of response at induction – posterior median (95% CrI) | | | | | | | |
| --- | --- | --- | --- | --- | --- | --- | --- |
| PBO | 2.15 (1.78, 2.63) | 1.90 (1.39, 2.63) | 2.35 (1.81, 3.01) | 1.17 (0.93, 1.44) | 2.34 (2.00, 2.73) | 3.31 (2.64, 4.04) | 3.29 (2.78, 3.84) |
| 0.46 (0.38, 0.56) | ADA 160/80/40mg | 0.88 (0.61, 1.29) | 1.08 (0.79, 1.49) | 0.54 (0.40, 0.72) | 1.09 (0.84, 1.38) | 1.52 (1.20, 1.87) | 1.51 (1.30, 1.76) |
| 0.53 (0.38, 0.72) | 1.13 (0.77, 1.65) | FIL 100mg | 1.23 (0.81, 1.84) | 0.61 (0.41, 0.89) | 1.22 (0.85, 1.74) | 1.70 (1.17, 2.46) | 1.72 (1.19, 2.44) |
| 0.43 (0.33, 0.55) | 0.92 (0.67, 1.26) | 0.81 (0.54, 1.23) | FIL 200mg | 0.49 (0.35, 0.69) | 0.99 (0.74, 1.34) | 1.39 (1.01, 1.93) | 1.40 (1.02, 1.88) |
| 0.85 (0.70, 1.07) | 1.86 (1.38, 2.50) | 1.64 (1.13, 2.41) | 2.03 (1.45, 2.82) | UST 90mg Q12W | 1.99 (1.66, 2.41) | 2.81 (2.07, 3.80) | 2.82 (2.12, 3.69) |
| 0.43 (0.37, 0.50) | 0.92 (0.73, 1.19) | 0.82 (0.57, 1.17) | 1.01 (0.75, 1.36) | 0.50 (0.41, 0.60) | UST 90mg Q8W | 1.41 (1.08, 1.80) | 1.40 (1.12, 1.75) |
| 0.30 (0.25, 0.38) | 0.66 (0.53, 0.83) | 0.59 (0.41, 0.85) | 0.72 (0.52, 0.99) | 0.36 (0.26, 0.48) | 0.71 (0.56, 0.93) | VDZ 300mg Q4W | 1.00 (0.84, 1.19) |
| 0.30 (0.26, 0.36) | 0.66 (0.57, 0.77) | 0.58 (0.41, 0.84) | 0.72 (0.53, 0.98) | 0.35 (0.27, 0.47) | 0.72 (0.57, 0.90) | 1.00 (0.84, 1.19) | VDZ 300mg Q8W |
| ADA: adalimumab; FIL: filgotinib; PBO: placebo; UST: ustekinumab; VDZ: vedolizumab  *Pink : Relative risk >1 (column treatment has greater RR of achieving the outcome than row treatment, if CrI does not include 1 this difference is significant)*  *Blue: Relative risk <1*  *Each cell represents the comparison (median relative risk and 95% CrI) of the column treatment versus the row treatment* | | | | | | | |

## Sensitivity analysis *–* biologic-naïve

### Induction phase

The sensitivity analysis excluded UNFI because the definition of biologic-naïve differed from other study (patients were required have biologic non-failure). Ustekinumab dropped from the network resulting in a slight change in efficacy estimates; however, the ranking of treatments remained the same.

**Supplementary table 23.** Sensitivity analysis: modelled probabilities of response and remission at induction.

| Treatment | Modelled probability of response – posterior median (95% CrI) | | | | | |  |
| --- | --- | --- | --- | --- | --- | --- | --- |
|  | **Base case** | | **Sensitivity (biologic naïve) – UNIFI excluded** | | | | |
|  | **Response** | **Remission** | **Response** | | **Remission** | | |
| IFX 5 mg/kg | 0.605 (0.542, 0.662) | 0.235 (0.188, 0.289) | 0.603 (0.540, 0.668) | 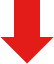 | 0.239 (0.191, 0.296) | 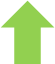 | |
| VDZ 300 mg | 0.580 (0.496, 0.664) | 0.217 (0.160, 0.288) | 0.583 (0.494, 0.664) | 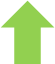 | 0.222 (0.161, 0.291) | 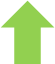 | |
| UST 6 mg/kg | 0.539 (0.433, 0.634) | 0.186 (0.123, 0.261) | – |  | – |  | |
| TOFA 10 mg | 0.512 (0.413, 0.614) | 0.169 (0.113, 0.244) | 0.514 (0.407, 0.612) | 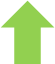 | 0.174 (0.114, 0.247) | 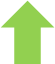 | |
| GOL 200/100 mg | 0.486 (0.407, 0.566) | 0.154 (0.110, 0.208) | 0.485 (0.404, 0.570) | 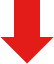 | 0.156 (0.111, 0.214) | 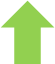 | |
| FIL 200 mg | 0.468 (0.373, 0.564) | 0.143 (0.095, 0.206) | 0.467 (0.372, 0.565) | 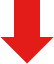 | 0.146 (0.096, 0.212) | **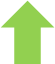** | |
| ADA 160/80/40 mg | 0.436 (0.370, 0.508) | 0.126 (0.092, 0.168) | 0.438 (0.368, 0.510) | 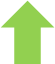 | 0.129 (0.094, 0.172) | 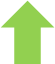 | |
| FIL 100 mg | 0.387 (0.297, 0.479) | 0.101 (0.064, 0.150) | 0.387 (0.297, 0.479) | 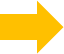 | 0.104 (0.066, 0.153) | 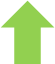 | |
| PBO | 0.289 (0.272, 0.304) | 0.061 (0.054, 0.070) | 0.290 (0.270, 0.310) | 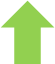 | 0.063 (0.054, 0.073) | 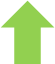 | |
| ADA: adalimumab; CrI: credible interval; FIL: filgotinib; GOL: golimumab; IFX: infliximab; PBO: placebo; TOFA: tofacitinib; UST: ustekinumab; VDZ: vedolizumab. | | | | | | |  |

#### Maintenance phase

Outcomes of the sensitivity analyses are shown in **Supplementary table 24**.

- **Sensitivity analysis 1:** exclusion of treat-through trials (ACT1, ULTRA 2 and VARSITY) meant adalimumab and infliximab dropped from the network leading to slight changes in efficacy estimates for response or remission; vedolizumab 300 mg Q3W was estimated to be more effective than in the base case.
- **Sensitivity analysis 2:** maintenance outcomes of the VARSITY trial were identified as uncertain. Exclusion of VARSITY resulted in a minor adjustment in efficacy estimates for response or remission.
- **Sensitivity analysis 3**: use of re-weighting methods described in Hernandez *et al*. (Supplementary table 24) resulted in slight changes to efficacy estimates for response or remission, suggesting that the efficacy estimates for infliximab 5 mg/kg may be underestimated in the base case, but the effect is minimal.
- **Sensitivity analysis 4a and 4b:** re-weighting using the highest and lowest placebo estimates led to small changes in efficacy estimates for all treatments.

**Supplementary table 24.** Sensitivity analysis: modelled probabilities of response at maintenance.

| Treatment | **Modelled probability of response – posterior median (95% CrI)** | | | | | | | | | | |
| --- | --- | --- | --- | --- | --- | --- | --- | --- | --- | --- | --- |
|  | **Base case** | **Sensitivity 1 (trial design) – ACT 1, ULTRA 2, VARSITY excluded** | | **Sensitivity 2 (unpublished data) – VARSITY excluded** | | **Sensitivity 3 (Hernandez et al. re-weighting)** | | **Sensitivity 4a (ACT 1 High placebo estimate)** | | **Sensitivity 4b (ACT 1 Low placebo estimate)** | |
|  | **Response** | **Response** | | **Response** | | **Response** | | **Response** | | **Response** | |
| TOFA 10 mg | 0.711  (0.465, 0.878) | 0.692  (0.324, 0.933) | 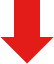 | 0.714  (0.480, 0.879) | 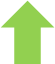 | 0.673  (0.540, 0.781) | 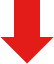 | 0.714  (0.478, 0.879) | 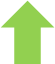 | 0.694  (0.411, 0.891) | 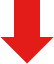 |
| TOFA 5 mg | 0.662  (0.424, 0.850) | 0.638  (0.277, 0.910) | 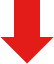 | 0.663  (0.426, 0.848) | 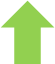 | 0.624  (0.487, 0.741) | 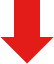 | 0.666  (0.424, 0.850) | 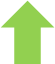 | 0.645  (0.359, 0.863) | 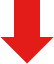 |
| FIL 200 mg | 0.643  (0.379, 0.846) | 0.621  (0.251, 0.906) | 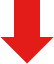 | 0.644  (0.383, 0.843) | 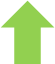 | 0.601  (0.446, 0.740) | 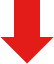 | 0.646  (0.392, 0.845) | 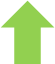 | 0.626  (0.328, 0.857) | 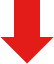 |
| VDZ 108 mg SC Q2W | 0.637  (0.353, 0.853) | 0.657  (0.276, 0.925) | 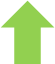 | 0.682  (0.407, 0.880) | 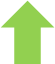 | 0.602  (0.422, 0.768) | 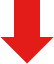 | 0.641  (0.371, 0.854) | 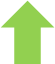 | 0.619  (0.309, 0.863) | 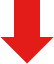 |
| VDZ 300 mg Q4W | 0.594  (0.335, 0.801) | 0.614  (0.250, 0.905) | 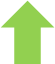 | 0.641  (0.392, 0.837) | 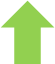 | 0.562  (0.417, 0.700) | 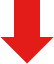 | 0.598  (0.348, 0.813) | 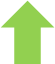 | 0.575  (0.288, 0.826) | 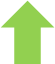 |
| VDZ 300 mg Q8W | 0.591  (0.356, 0.787) | 0.652  (0.286, 0.916) | 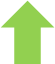 | 0.673  (0.435, 0.854) | 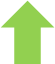 | 0.562  (0.457, 0.668) | 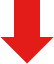 | 0.593  (0.360, 0.796) | 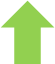 | 0.571  (0.297, 0.809) | 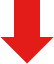 |
| IFX 5 mg/kg | 0.545  (0.286, 0.797) |  |  | 0.549  (0.293, 0.790) | 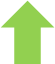 | 0.585  (0.433, 0.730) | 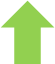 | 0.528  (0.287, 0.758) | 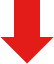 | 0.635  (0.323, 0.875) | 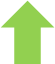 |
| UST 90 mg Q8W | 0.529  (0.290, 0.758) | 0.511  (0.175, 0.841) | 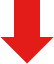 | 0.529  (0.287, 0.759) | 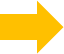 | 0.483  (0.344, 0.618) | 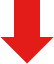 | 0.527  (0.291, 0.753) | 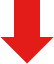 | 0.507  (0.234, 0.774) | 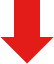 |
| UST 90 mg Q12W | 0.528  (0.280, 0.748) | 0.504  (0.168, 0.840) | 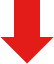 | 0.525  (0.283, 0.752) | 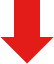 | 0.482  (0.346, 0.619) | 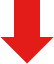 | 0.520  (0.263, 0.765) | 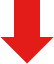 | 0.506  (0.236, 0.772) | 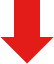 |
| ADA 160/80/40 mg | 0.494  (0.265, 0.718) |  |  | 0.374  (0.165, 0.626) | 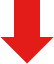 | 0.476  (0.369, 0.587) | 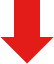 | 0.498  (0.271, 0.725) | **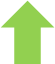** | 0.475  (0.219, 0.740) | 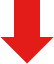 |
| GOL 100 mg Q4W | 0.477  (0.266, 0.704) | 0.454  (0.145, 0.791) | 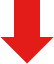 | 0.479  (0.256, 0.712) | 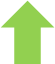 | 0.435  (0.326, 0.546) | 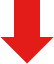 | 0.480  (0.260, 0.704) | **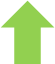** | 0.459  (0.205, 0.727) | 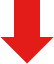 |
| GOL 50 mg Q4W | 0.462  (0.253, 0.691) | 0.431  (0.137, 0.782) | 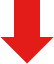 | 0.461  (0.242, 0.688) | 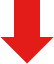 | 0.416  (0.313, 0.531) | 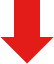 | 0.461  (0.246, 0.690) | 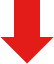 | 0.441  (0.193, 0.711) | 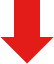 |
| FIL 100 mg | 0.370  (0.163, 0.626) | 0.352  (0.092, 0.730) | 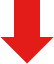 | 0.371  (0.169, 0.629) | 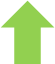 | 0.331  (0.207, 0.476) | 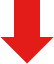 | 0.375  (0.168, 0.628) | **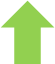** | 0.355  (0.130, 0.650) | 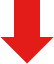 |
| PBO | 0.308  (0.152, 0.511) | 0.283  (0.072, 0.641) | 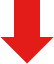 | 0.307  (0.150, 0.506) | 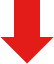 | 0.270  (0.248, 0.291) | 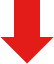 | 0.310  (0.154, 0.510) | **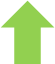** | 0.290  (0.114, 0.537) | 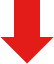 |
| ADA: adalimumab; CrI: credible interval; FIL: filgotinib; GOL: golimumab; IFX: infliximab; PBO: placebo; TOFA: tofacitinib; UST: ustekinumab; VDZ: vedolizumab. | | | | | | | | | | | |

**Supplementary table 25.** Sensitivity analysis: modelled probabilities of remission at maintenance.

| Treatment | Base case | Sensitivity 1 (trial design) – ACT 1, ULTRA 2, VARSITY excluded | | Sensitivity 2 (unpublished data) – VARSITY excluded | | Sensitivity 3 (Hernandez et al. re-weighting) | | Sensitivity 4a (ACT 1 High placebo estimate) | | Sensitivity 4b (ACT 1 Low placebo estimate) | |
| --- | --- | --- | --- | --- | --- | --- | --- | --- | --- | --- | --- |
|  | **Remission** | **Remission** | | **Remission** | | **Remission** | | **Remission** | | **Remission** | |
| TOFA 10 mg | 0.514  (0.275, 0.743) | 0.487  (0.161, 0.833) | 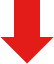 | 0.525  (0.289, 0.745) | 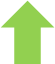 | 0.477  (0.342, 0.607) | 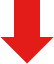 | 0.519  (0.284, 0.744) | 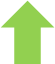 | 0.495  (0.228, 0.762) | 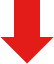 |
| TOFA 5 mg | 0.460  (0.237, 0.699) | 0.429  (0.131, 0.791) | 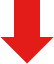 | 0.465  (0.243, 0.698) | 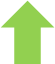 | 0.425  (0.295, 0.558) | 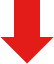 | 0.465  (0.240, 0.700) | 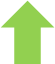 | 0.441  (0.189, 0.718) | 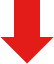 |
| FIL 200 mg | 0.440  (0.207, 0.688) | 0.411  (0.114, 0.781) | 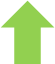 | 0.443  (0.212, 0.691) | 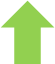 | 0.402  (0.260, 0.556) | 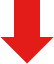 | 0.444  (0.215, 0.691) | 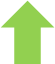 | 0.421  (0.167, 0.708) | 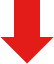 |
| VDZ 108 mg SC Q2W | 0.432  (0.186, 0.700) | 0.449  (0.128, 0.818) |  | 0.486  (0.232, 0.751) |  | 0.402  (0.240, 0.592) |  | 0.438  (0.198, 0.705) |  | 0.414  (0.154, 0.717) |  |
| VDZ 300 mg Q4W | 0.389  (0.174, 0.635) | 0.405  (0.113, 0.781) |  | 0.441  (0.217, 0.684) |  | 0.363  (0.236, 0.509) |  | 0.394  (0.182, 0.645) |  | 0.371  (0.140, 0.663) |  |
| VDZ 300 mg Q8W | 0.387  (0.188, 0.614) | 0.444  (0.140, 0.802) |  | 0.477  (0.251, 0.708) |  | 0.364  (0.269, 0.474) |  | 0.390  (0.191, 0.622) |  | 0.366  (0.146, 0.640) |  |
| IFX 5 mg/kg | 0.343  (0.138, 0.621) |  |  | 0.350  (0.148, 0.621) |  | 0.385  (0.250, 0.542) |  | 0.320  (0.125, 0.582) |  | 0.431  (0.163, 0.737) |  |
| UST 90 mg Q8W | 0.328  (0.141, 0.574) | 0.307  (0.071, 0.682) |  | 0.331  (0.141, 0.575) |  | 0.292  (0.181, 0.424) |  | 0.328  (0.141, 0.573) |  | 0.308  (0.106, 0.592) |  |
| UST 90 mg Q12W | 0.327  (0.134, 0.562) | 0.302  (0.067, 0.679) |  | 0.328  (0.140, 0.565) |  | 0.291  (0.184, 0.421) |  | 0.327  (0.143, 0.568) |  | 0.307  (0.108, 0.589) |  |
| ADA 160/80/40 mg | 0.299  (0.127, 0.527) |  |  | 0.204  (0.068, 0.425) |  | 0.286  (0.199, 0.390) |  | 0.301  (0.130, 0.531) |  | 0.280  (0.098, 0.551) |  |
| GOL 100 mg Q4W | 0.283  (0.125, 0.510) | 0.257  (0.056, 0.610) |  | 0.289  (0.122, 0.518) |  | 0.251  (0.169, 0.352) |  | 0.286  (0.123, 0.509) |  | 0.267  (0.090, 0.534) |  |
| GOL 50 mg Q4W | 0.270  (0.117, 0.495) | 0.242  (0.051, 0.597) |  | 0.272  (0.112, 0.493) |  | 0.237  (0.159, 0.336) |  | 0.269  (0.114, 0.492) |  | 0.251  (0.083, 0.514) |  |
| FIL 100 mg | 0.198  (0.069, 0.427) | 0.181  (0.031, 0.529) |  | 0.201  (0.071, 0.431) |  | 0.173  (0.092, 0.286) |  | 0.202  (0.069, 0.426) |  | 0.186  (0.050, 0.446) |  |
| PBO | 0.154  (0.060, 0.313) | 0.134  (0.023, 0.432) |  | 0.155  (0.061, 0.311) |  | 0.132  (0.115, 0.149) |  | 0.156  (0.062, 0.312) |  | 0.141  (0.042, 0.336) |  |
| ADA: adalimumab; CrI: credible interval; FIL: filgotinib; GOL: golimumab; IFX: infliximab; PBO: placebo; TOFA: tofacitinib; UST: ustekinumab; VDZ: vedolizumab. | | | | | | | | | | | |

## Sensitivity analysis *–* biologic-experienced

#### Induction phase

The VARSITY and ULTRA 2 trials were excluded owing to differences in the definition of biologic-naïve. Adalimumab was dropped from the network resulting in a slight reduction in efficacy estimates across treatments suggesting the results of the base case analysis was not sensitive to the inclusion of these trials.

**Supplementary table 26.** Sensitivity analysis: modelled probabilities of response and remission at induction.

| Treatment | Modelled probability of response – posterior median (95% CrI) | | | | | |
| --- | --- | --- | --- | --- | --- | --- |
|  | **Base case** | | **Sensitivity (population definition) – VARSITY and ULTRA 2 excluded** | | | |
|  | **Response** | **Remission** | **Response** | | **Remission** | |
| FIL 200mg | 0.385 (0.293, 0.494) | 0.070 (0.041, 0.116) | 0.367 (0.270, 0.474) |  | 0.055 (0.030, 0.093) |  |
| UST 6mg/kg | 0.344 (0.252, 0.445) | 0.056 (0.032, 0.094) | 0.329 (0.240, 0.432) |  | 0.044 (0.024, 0.077) |  |
| TOF 10mg | 0.339 (0.251, 0.436) | 0.055 (0.031, 0.091) | 0.328 (0.239, 0.422) |  | 0.044 (0.024, 0.076) |  |
| VDZ 300mg | 0.307 (0.202, 0.427) | 0.045 (0.021, 0.087) | 0.253 (0.136, 0.410) |  | 0.027 (0.009, 0.069) |  |
| FIL 100mg | 0.251 (0.172, 0.340) | 0.032 (0.016, 0.057) | 0.231 (0.157, 0.327) |  | 0.023 (0.011, 0.044) |  |
| ADA 160/80/40mg | 0.150 (0.090, 0.232) | 0.013 (0.006, 0.028) |  |  |  |  |
| PBO | 0.118 (0.115, 0.120) | 0.009 (0.007, 0.011) | 0.106 (0.104, 0.109) |  | 0.006 (0.005, 0.008) |  |
| ADA: adalimumab; CrI: credible interval; FIL: filgotinib; GOL: golimumab; IFX: infliximab; PBO: placebo; TOF: tofacitinib; UST: ustekinumab; VDZ: vedolizumab. | | | | | | |

#### Maintenance phase

Outcomes of the sensitivity analyses are shown in **Supplementary table 27**.

- **Sensitivity analysis 1:** exclusion of treat-through trials (ACT1, ULTRA 2 and VARSITY) meant adalimumab dropped from the network resulting in slight changes to efficacy estimates for response and remission, which were not significant.
- **Sensitivity analysis 2:** exclusion of the VARSITY trial, which introduced uncertainty surrounding maintenance outcomes resulted in minor changes in efficacy estimates for response and remission.
- **Sensitivity analysis 3:** use of re-weighting methods described in Hernandez *et al*. (Supplementary table 27) have minimal on efficacy estimates for response and remission.

**Supplementary table 27.** Sensitivity analysis: modelled probabilities of response and remission at maintenance.

| Treatment | Modelled probability of response – posterior median (95% CrI) | | | | | | | | | | | | | |
| --- | --- | --- | --- | --- | --- | --- | --- | --- | --- | --- | --- | --- | --- | --- |
|  | **Base case** | | **Sensitivity 1 (trial design) – ACT 1, ULTRA 2, VARISTY excluded** | | | | **Sensitivity 2 (unpublished data) –, VARISTY excluded** | | | | **Sensitivity 3 (Hernandez et al. re-weighting)** | | | |
|  | **Response** | **Remission** | **Response** | | **Remission** | | **Response** | | **Remission** | | **Response** | | **Remission** | |
| TOFA 10mg | 0.463 (0.217, 0.724) | 0.252 (0.088, 0.506) | 0.455 (0.170, 0.767) |  | 0.235 (0.059, 0.549) |  | 0.461 (0.222, 0.716) |  | 0.242 (0.086, 0.494) |  | 0.467 (0.183, 0.774) |  | 0.253 (0.067, 0.567) |  |
| VDZ 108mg SC Q2W | 0.440 (0.150, 0.772) | 0.233 (0.053, 0.564) | 0.478 (0.142, 0.847) |  | 0.254 (0.046, 0.660) |  | 0.493 (0.179, 0.808) |  | 0.267 (0.063, 0.612) |  | 0.439 (0.124, 0.802) |  | 0.232 (0.041, 0.606) |  |
| VDZ 300mg Q8W | 0.362 (0.140, 0.641) | 0.175 (0.049, 0.411) | 0.418 (0.131, 0.771) |  | 0.205 (0.043, 0.554) |  | 0.429 (0.174, 0.717) |  | 0.216 (0.059, 0.483) |  | 0.359 (0.109, 0.681) |  | 0.172 (0.035, 0.465) |  |
| VDZ 300mg Q4W | 0.340 (0.115, 0.642) | 0.160 (0.038, 0.410) | 0.366 (0.102, 0.736) |  | 0.169 (0.030, 0.513) |  | 0.376 (0.136, 0.681) |  | 0.181 (0.044, 0.448) |  | 0.341 (0.089, 0.691) |  | 0.161 (0.027, 0.472) |  |
| FIL 200mg | 0.334 (0.124, 0.615) | 0.157 (0.041, 0.387) | 0.331 (0.090, 0.676) |  | 0.148 (0.025, 0.437) |  | 0.333 (0.123, 0.612) |  | 0.149 (0.040, 0.374) |  | **0.338 (0.097, 0.669)** |  | **0.160 (0.029, 0.438)** |  |
| ADA 160/80/40mg | 0.332 (0.121, 0.619) | 0.154 (0.041, 0.390) |  |  |  |  | 0.242 (0.061, 0.554) |  | 0.096 (0.015, 0.320) |  | 0.327 (0.099, 0.650) |  | 0.152 (0.031, 0.423) |  |
| TOFA 5mg | 0.321 (0.126, 0.583) | 0.149 (0.042, 0.359) | 0.316 (0.088, 0.659) |  | 0.138 (0.024, 0.414) |  | 0.324 (0.133, 0.582) |  | 0.144 (0.042, 0.350) |  | 0.317 (0.085, 0.642) |  | 0.145 (0.025, 0.417) |  |
| UST 90mg Q8W | 0.268 (0.104, 0.505) | 0.117 (0.033, 0.289) | 0.265 (0.070, 0.587) |  | 0.107 (0.018, 0.339) |  | 0.269 (0.101, 0.508) |  | 0.111 (0.030, 0.283) |  | 0.276 (0.077, 0.575) |  | 0.119 (0.023, 0.351) |  |
| FIL 100mg | 0.234 (0.069, 0.524) | 0.096 (0.019, 0.301) | 0.226 (0.050, 0.575) |  | 0.086 (0.012, 0.334) |  | 0.229 (0.066, 0.516) |  | 0.089 (0.017, 0.289) |  | **0.236 (0.049, 0.572)** |  | **0.097 (0.013, 0.346)** |  |
| UST 90mg Q12W | 0.172 (0.056, 0.391) | 0.064 (0.015, 0.195) | 0.166 (0.035, 0.452) |  | 0.057 (0.008, 0.239) |  | 0.173 (0.051, 0.385) |  | 0.061 (0.012, 0.182) |  | 0.176 (0.040, 0.446) |  | 0.064 (0.010, 0.241) |  |
| PBO | 0.099 (0.032, 0.228) | 0.031 (0.007, 0.094) | 0.096 (0.019, 0.297) |  | 0.028 (0.004, 0.126) |  | 0.100 (0.033, 0.231) |  | 0.030 (0.007, 0.092) |  | 0.102 (0.022, 0.288) |  | 0.032 (0.004, 0.130) |  |
| ADA: adalimumab; CrI: credible interval; FIL: filgotinib; GOL: golimumab; IFX: infliximab; PBO: placebo; TOF: tofacitinib; UST: ustekinumab; VDZ: vedolizumab. | | | | | | | | | | | | | | |

# References

1. National Institute for Clinical Excellence.Ulcerative colitis: management. Clinical guideline 166., 2013. Available from: <https://www.nice.org.uk/guidance/cg166> (Accessed 12/12/2018).

2. Armuzzi A, De Pascalis B, Lupascu A, et al. Infliximab in the treatment of steroid-dependent ulcerative colitis. Eur Rev Med Pharmacol Sci. 2004;(8):231-3.

3. Bruce S, Marla D, Severine V, et al. Effects of increased vedolizumab dosing frequency on clinical remission and response in ulcerative colitis and crohn's disease. Inflammatory bowel diseases. 2014;(20):S67.

4. Colombel JF, Sands B, Hanauer S, et al. Long-term safety of vedolizumab for the treatment of ulcerative colitis or Crohn's disease. United European Gastroenterology Journal. 2013;(1):A106-A107.

5. Colombel JF, Panes J, D'Haens GR, et al. 945 HIGHER VERSUS STANDARD ADALIMUMAB MAINTENANCE REGIMENS IN PATIENTS WITH MODERATELY TO SEVERELY ACTIVE ULCERATIVE COLITIS: RESULTS FROM THE SERENE-UC MAINTENANCE STUDY. Gastroenterology. 2020;(158):S‐192‐.

6. DOP050. The efficacy of vedolizumab by disease activity and prior tumour necrosis factor-alpha antagonist failure in patients with ulcerative colitis or Crohn’s disease: post-hoc analyses from the GEMINI 1 and GEMINI 2 studies. Journal of Crohn's and Colitis. 2016;(10):S58-S58.

7. Danese S, Neurath M, Kopon A, et al. Apremilast for active ulcerative colitis: A phase 2, randomised, double-blind, placebo-controlled induction study. Journal of Crohn's and Colitis. 2018;(12):S004-S005.

8. Danese S, Neurath MF, Kopoń A, et al. Effects of Apremilast, an Oral Inhibitor of Phosphodiesterase 4, in a Randomized Trial of Patients With Active Ulcerative Colitis. Clin Gastroenterol Hepatol. 2020;(18):2526-2534.e9.

9. Euctr MT. A Phase 3, Randomized, Placebo-Controlled, Blinded, Multicenter Study of the Induction and Maintenance of Clinical Response and Remission by Vedolizumab (MLN0002) in Patients with Moderate to Severe Ulcerative Colitis. <Http://www.who.int/trialsearch/trial2.aspx>? Trialid=euctr2008-002782-32-mt. 2009;(

10. Euctr BG. A study to evaluate the dose response of JNJ-54781532 in participants with moderately to severely active ulcerative colitis (UC). <Http://www.who.int/trialsearch/trial2.aspx>? Trialid=euctr2013-000263-88-bg. 2014;(

11. Feagan BG, Rutgeerts P, Sands BE, et al. Vedolizumab as induction and maintenance therapy for ulcerative colitis. N Engl J Med. 2013;(369):699-710.

12. Feagan B, Kaser A, Smyth M, et al. Long-term efficacy of vedolizumab therapy for ulcerative colitis. United European Gastroenterology Journal. 2014;(2):A66-A67.

13. Feagan BG, Dubinsky MC, Lukas M, et al. Efficacy and safety of an additional 8 weeks of tofacitinib induction therapy: Results of the OCTAVE open study for tofacitinib 8-week induction non-responders. Journal of Crohn's and Colitis. 2018;(12):S50.

14. Gibson PR, Feagan BG, Sandborn WJ, et al. Maintenance of Efficacy and Continuing Safety of Golimumab for Active Ulcerative Colitis: PURSUIT-SC Maintenance Study Extension Through 1 Year. Clin Transl Gastroenterol. 2016;(7):e168.

15. Hibi T, Imai Y, Senoo A, et al. Efficacy and safety of golimumab 52-week maintenance therapy in Japanese patients with moderate to severely active ulcerative colitis: a phase 3, double-blind, randomized, placebo-controlled study-(PURSUIT-J study). J Gastroenterol. 2017;(52):1101-1111.

16. Kaser A, James A. Long-term effectiveness and safety of vedolizumab in patients with ulcerative colitis: 5-year cumulative exposure of gemini 1 completers rolling into the gemini open-label extension study. Gut. 2017;(66):A120.

17. Leiper K, Martin K, Ellis A, et al. Randomised placebo-controlled trial of rituximab (anti-CD20) in active ulcerative colitis. Gut. 2011;(60):1520.

18. Lichtenstein GR, Loftus EV, Bloom S, et al. Tofacitinib, an oral janus kinase inhibitor, in the treatment of ulcerative colitis: An interim analysis of an open-label, long-term extension study with up to 4.9 years of treatment. United European Gastroenterology Journal. 2018;(6):A255-A256.

19. Panaccione R, Ghosh S, Middleton S, et al. Combination therapy with infliximab and azathioprine is superior to monotherapy with either agent in ulcerative colitis. Gastroenterology. 2014;(146):392-400.e3.

20. Panes J, Colombel JF, D'Haens GR, et al. High versus standard adalimumab induction dosing regime ns in patients with moderately to severely active ulcerative colitis : results from the SERENE-UC induction study. United european gastroenterology journal. 2019;(7):118‐.

21. Parikh A, Leach T, Wyant T, et al. Vedolizumab for the treatment of active ulcerative colitis: a randomized controlled phase 2 dose-ranging study. Inflammatory bowel diseases. 2012;(18):1470‐1479.

22. Parikh A. Efficacy of vedolizumab in ulcerative colitis by prior treatment failure in gemini i, a randomized, placebo-controlled, double-blind, multicenter trial. Inflammatory Bowel Diseases. 2012;(18):S26.

23. Parikh A, Fox I, Leach T, et al. Long-term clinical experience with vedolizumab in patients with inflammatory bowel disease. Inflammatory Bowel Diseases. 2013;(19):1691-1699.

24. Probert CS, Hearing SD, Schreiber S, et al. Infliximab in moderately severe glucocorticoid resistant ulcerative colitis: a randomised controlled trial. Gut. 2003;(52):998-1002.

25. Reinisch W, Sandborn WJ, Rutgeerts P, et al. Long-term infliximab maintenance therapy for ulcerative colitis: the ACT-1 and -2 extension studies. Inflamm Bowel Dis. 2012;(18):201-11.

26. Reinisch W, Sandborn WJ, Panaccione R, et al. 52-week efficacy of adalimumab in patients with moderately to severely active ulcerative colitis who failed corticosteroids and/or immunosuppressants. Inflammatory bowel diseases. 2013;(19):1700‐1709.

27. Reinisch W, Gibson PR, Sandborn WJ, et al. Long-term Benefit of Golimumab for Patients with Moderately-to-Severely Active Ulcerative Colitis: Results from the PURSUIT-Maintenance Extension. J Crohns Colitis. 2018;(

28. Rutgeerts P, Feagan BG, Marano CW, et al. Randomised clinical trial: a placebo-controlled study of intravenous golimumab induction therapy for ulcerative colitis. Aliment Pharmacol Ther. 2015;(42):504-14.

29. Sandborn WJ, Ghosh S, Panes J, et al. Tofacitinib, an oral Janus kinase inhibitor, in active ulcerative colitis. N Engl J Med. 2012;(367):616-24.

30. Adalimumab in the Treatment of Moderate-to-Severe Ulcerative Colitis: ULTRA 2 Trial Results. Gastroenterol Hepatol (N Y). 2013;(9):317-20.

31. Sandborn WJ, Ghosh S, Panés J, et al. Efficacy and safety of upadacitinib as an induction therapy for patients with moderately-toseverely active ulcerative colitis: Data from the phase 2b study u-achieve. United European Gastroenterology Journal. 2018;(6):A74-A75.

32. Sandborn WJ, Ghosh S, Panes J, et al. Efficacy of Upadacitinib in a Randomized Trial of Patients With Active Ulcerative Colitis. Gastroenterology. 2020;(158):2139-2149.e14.

33. Sands BE, Sandborn WJ, Feagan BG, et al. Peficitinib, an Oral Janus Kinase Inhibitor, in Moderate-to-severe Ulcerative Colitis: Results From a Randomised, Phase 2 Study. J Crohns Colitis. 2018;(12):1158-1169.

34. Sands BE, Sandborn WJ, Panaccione R, et al. Safety and efficacy of ustekinumab induction therapy in patients with moderate to severe ulcerative colitis: Results from the phase 3 unifi study. United European Gastroenterology Journal. 2018;(6):1586.

35. Nct. An Efficacy and Safety Study of Vedolizumab Intravenous (IV) Compared to Adalimumab Subcutaneous (SC) in Participants With Ulcerative Colitis. <Https://clinicaltrials.gov/show/nct02497469>. 2015;(

36. Reinisch W, Sandborn WJ, Hommes DW, et al. Adalimumab for induction of clinical remission in moderately to severely active ulcerative colitis: results of a randomised controlled trial. Gut. 2011;(60):780-7.

37. Sandborn WJ, D'Haens GR, Colombel JF, et al. One-year response and remission rates in ulcerative colitis patients with week 8 response to adalimumab: subanalysis of ultra 2. Gastroenterology. 2012;(142):S565.

38. Sandborn WJ, van Assche G, Reinisch W, et al. Adalimumab induces and maintains clinical remission in patients with moderate-to-severe ulcerative colitis. Gastroenterology. 2012;(142):257-65.e1-3.

39. Sands BE, Peyrin-Biroulet L, Loftus EV, et al. Vedolizumab versus Adalimumab for Moderate-to-Severe Ulcerative Colitis. New England Journal of Medicine. 2019;(381):1215-1226.

40. Sandborn WJ, Feagan BG, Marano C, et al. Subcutaneous golimumab induces clinical response and remission in patients with moderate-to-severe ulcerative colitis. Gastroenterology. 2014;(146):85-95; quiz e14-5.

41. Sandborn WJ, Feagan BG, Marano C, et al. Subcutaneous golimumab maintains clinical response in patients with moderate-to-severe ulcerative colitis. Gastroenterology. 2014;(146):96-109.e1.

42. Rutgeerts P, Sandborn WJ, Feagan BG, et al. Infliximab for induction and maintenance therapy for ulcerative colitis. N Engl J Med. 2005;(353):2462-76.

43. Kobayashi T, Suzuki Y, Motoya S, et al. First trough level of infliximab at week 2 predicts future outcomes of induction therapy in ulcerative colitis-results from a multicenter prospective randomized controlled trial and its post hoc analysis. Journal of gastroenterology. 2016;(51):241‐251.

44. Jiang XL, Cui HF, Gao J, et al. Low-dose Infliximab for Induction and Maintenance Treatment in Chinese Patients With Moderate to Severe Active Ulcerative Colitis. Journal of clinical gastroenterology. 2015;(49):582‐588.

45. Nct. A Study to Evaluate the Effectiveness and Safety of Infliximab in Chinese Patients With Active Ulcerative Colitis. <Https://clinicaltrials.gov/show/nct01551290>. 2012;(

46. Sandborn WJ, Su C, Sands BE, et al. Tofacitinib as Induction and Maintenance Therapy for Ulcerative Colitis. N Engl J Med. 2017;(376):1723-1736.

47. Dubinsky MC, Clarke K, Klaus JG, et al. Time to loss of efficacy following tofacitinib interruption in patients with ulcerative colitis: Results from octave sustain. United European Gastroenterology Journal. 2018;(6):A121.

48. Sands BE, Peyrin-Biroulet L, Marano C, et al. P312 Efficacy in biologic failure and non-biologic-failure populations in a Phase 3 study of ustekinumab in moderate–severe ulcerative colitis: UNIFI. Journal of Crohn's and Colitis. 2019;(13):S256-S257.

49. Feagan BG, Rubin DT, Danese S, et al. Efficacy of Vedolizumab Induction and Maintenance Therapy in Patients With Ulcerative Colitis, Regardless of Prior Exposure to Tumor Necrosis Factor Antagonists. Clin Gastroenterol Hepatol. 2017;(15):229-239.e5.

50. Sandborn WJ, Baert F, Danese S, et al. Efficacy and Safety of Vedolizumab Subcutaneous Formulation in a Randomized Trial of Patients With Ulcerative Colitis. Gastroenterology. 2020;(158):562-572.e12.

51. Rutgeerts P, Sandborn WJ, Feagan BG, et al. Infliximab for induction and maintenance therapy for ulcerative colitis. N Engl J Med. 2005;(353):2462–76.

52. Feagan BG, Rubin DT, Danese S, et al. Efficacy of vedolizumab induction and maintenance therapy in patients with ulcerative colitis, regardless of prior exposure to tumor necrosis factor antagonists. Clin Gastroenterol Hepatol. 2017;(15):229–39 e5.

53. Jiang XL, Cui HF, Gao J, et al. Low-dose infliximab for induction and maintenance treatment in Chinese patients with moderate to severe active ulcerative colitis. J Clin Gastroenterol. 2015;(49):582–8.

54. Kobayashi T, Suzuki Y, Motoya S, et al. First trough level of infliximab at week 2 predicts future outcomes of induction therapy in ulcerative colitis-results from a multicenter prospective randomized controlled trial and its post hoc analysis. J Gastroenterol. 2016;(51):241–51.

55. ClinicalTrials.gov. A study to evaluate the effectiveness and safety of infliximab in Chinese patients with active ulcerative colitis. 2015. Available from: <https://clinicaltrials.gov/ct2/show/NCT01551290> (Accessed 20 September 2021).

56. Sandborn WJ, Su C, Sands BE, et al. Tofacitinib as induction and maintenance therapy for ulcerative colitis. N Engl J Med. 2017;(376):1723–1736.

57. Sandborn WJ, Feagan BG, Marano C, et al. Subcutaneous golimumab maintains clinical response in patients with moderate-to-severe ulcerative colitis. Gastroenterology. 2014;(146):96–109.e1.

58. Feagan BG, Danese S, Loftus EV, Jr., et al. Filgotinib as induction and maintenance therapy for ulcerative colitis (SELECTION): a phase 2b/3 double-blind, randomised, placebo-controlled trial. Lancet. 2021;(397):2372–2384.

59. Reinisch W, Sandborn WJ, Hommes DW, et al. Adalimumab for induction of clinical remission in moderately to severely active ulcerative colitis: results of a randomised controlled trial. Gut. 2011;(60):780–7.

60. Sandborn WJ, van Assche G, Reinisch W, et al. Adalimumab induces and maintains clinical remission in patients with moderate-to-severe ulcerative colitis. Gastroenterology. 2012;(142):257–65.e1–3.

61. Sands BE, Peyrin-Biroulet L, Loftus EV, et al. Vedolizumab versus adalimumab for moderate-to-severe ulcerative colitis. New Eng J Med. 2019;(381):1215–26.

62. Dubinsky MC, Clarke K, Klaus JG, et al. Time to loss of efficacy following tofacitinib interruption in patients with ulcerative colitis: results from octave sustain. J United Eur Gastroent. 2018;(6):A121.

63. Sands BE, Peyrin-Biroulet L, Marano C, et al. Efficacy in biologic failure and non-biologic-failure populations in a Phase 3 study of ustekinumab in moderate–severe ulcerative colitis: UNIFI. J Crohn's Colitis. 2019;(13):S256–7.

64. Sandborn WJ, Baert F, Danese S, et al. Efficacy and safety of vedolizumab subcutaneous formulation in a randomized trial of patients with ulcerative colitis. Gastroenterology. 2020;(158):562–72.e12.
